# The Demand Avoidance and Autonomy Questionnaire (DAAQ): Development of a new measure of demand avoidance

**DOI:** 10.1371/journal.pone.0345827

**Published:** 2026-11-11

**Authors:** Noelle E. Carlozzi, Jin-Shei Lai, Wendy L. Lombard, Jonathan P. Troost, Christopher M. Graves, Jennifer A. Miner, Madison Fansher, Casey Ehrlich

**Affiliations:** 1 Department of Physical Medicine and Rehabilitation, University of Michigan, Ann Arbor, Michigan United States of America; 2 Department of Medical Social Sciences, Northwestern University, Chicago, Illinois United States of America; 3 Michigan Institute for Clinical & Health Research, Ann Arbor, Michigan United States of America; 4 At Peace Parents, Chelsea, Michigan United States of America; Universitat Jaume 1, SPAIN

## Abstract

**Background:**

Pathological demand avoidance (PDA; also described as persistent/extreme demand avoidance or a pervasive drive for autonomy) is a complex, debated construct. It most often refers to a pattern of behavior characterized by intense distress and avoidance of both external and internal demands across home and community settings and is frequently discussed as a profile of autism spectrum disorder (ASD). Progress toward resolving the scientific controversy around PDA has been limited by the lack of consensus definitions and robust clinical measurement tools. Existing PDA measures (e.g., the Extreme Demand Avoidance Questionnaire; EDA-Q/EDA-8) have conceptual and psychometric limitations, including limited assessment of functional impact on basic needs (i.e., hygiene, eating, sleeping) and toileting. We developed the Demand Avoidance and Autonomy Questionnaire (DAAQ) to capture PDA-relevant behavioral expression and basic-needs impacts using contemporary measurement-development methods.

**Methods:**

Self-identified adult parents/legal guardians of children aged 4–17 with a suspected PDA profile (N = 707) completed the DAAQ 60-item pool and other parent proxy measures (EDA-8, CAST, SDQ, and PROMIS Parent Proxy–Global Health). Analyses included exploratory and confirmatory factor analyses and item response theory (graded response models), differential item functioning (DIF), as well as reliability, convergent/discriminant validity, and known-groups validity.

**Results:**

Factor analysis supported retention of 36 items across three factors in the DAAQ: Behavioral Expression (20 items), Basic Needs (10 items), and Toileting (6 items). Differential item functioning for child age, education, school type, and sex; and parent age and education did not result in any items being deleted. Convergent validity was supported via moderate correlations with the EDA-8, and discriminant validity was supported by generally negligible-to-small associations with prespecified measures. Known-groups comparisons differentiated groups as hypothesized (e.g., higher DAAQ scores among children with higher demand avoidance, greater basic needs difficulties, and co-occurring ASD).

**Conclusions:**

This paper describes a new, methodologically sound measurement tool to facilitate screening for and characterizing demand avoidance among children and adolescents in research and clinical settings. Given the controversy surrounding the existence of PDA and how to classify it, DAAQ scores should not be interpreted as diagnostic, and associations with autism-screening measures should be interpreted cautiously. The rigorous measurement development approach, strong preliminary reliability and validity, and inclusion of a general PDA behavior factor as well as basic needs and toileting impact factors are strengths of the DAAQ. Future work should evaluate performance in clinician-characterized samples, develop self-report versions, and further assess validity (e.g., predictive validity and sensitivity/specificity).

## Introduction

Pathological demand avoidance (PDA; also described as “persistent” or “extreme” demand avoidance or a pervasive drive for autonomy [1]) is a complex, debated construct. It most often refers to a pattern of behavior characterized by intense distress and avoidance of both external and internal demands across home and community settings, and is frequently discussed in relation to autism spectrum disorder (ASD). Progress toward resolving the scientific controversy around PDA has been limited by the lack of consensus and robust clinical measurement tools [2]. Terminology is also contested, as “Pathological Demand Avoidance” may be experienced as stigmatizing by some individuals and families, whereas autonomy-centered language may be perceived as less pathologizing; however, any label can contribute to stigma or prejudice if applied without careful clinical formulation and attention to the individual’s perspective.

When a demand cannot be avoided or managed, individuals with PDA may involuntarily experience intense physiological or emotional reactions, including meltdowns; fight, flight, freeze, or fawn responses; shutdown episodes; anger and aggressive outbursts; and/or panic [3–6].

Although PDA is frequently positioned as a profile of autism, its ontology remains contested. Some authors argue that extreme demand avoidance is not specific to autism and may be better understood in relation to interacting comorbidities (e.g., anxiety), trauma-related mechanisms, and contextual factors [7]. Still others believe that PDA is a form of Attachment Disorder [8,9] or Personality Disorder [9]. Clinically, we anecdotally observe that sustained nervous system activation (i.e., the frequent experience of fight, flight, freeze, or shutdown) may also interfere with fulfilling basic needs (i.e., hygiene, eating, sleeping and toileting). Regardless of etiology, PDA is associated with profound challenges for affected individuals and their families [5].

The construct of PDA was first described by Elizabeth Newson in the early 1980s based on a case series of referrals to a specialist clinic in Nottingham, UK, and was formally introduced in the peer-reviewed literature in 2003 [3]. Newson characterized PDA as “a distinct behavioral profile involving obsessional avoidance of the ordinary demands of life, coupled with a degree of sociability that allowed social manipulation as a primary avoidance strategy [3].” Her account used “ordinary demands” to encompass “normal expectations,” ordinary group demands in nursery school, educational demands, organizational rules, and adult-directed tasks. Newson also emphasized the impact of these behaviors on parents and teachers, describing the children as particularly difficult to manage and teach. However, Newson did not provide a discrete or culturally neutral operational definition of what makes a demand “ordinary.” What is regarded as an ordinary or developmentally typical expectation may vary across families, educational settings, social contexts, and cultures [10].

Autonomy-centered theories extend this idea by proposing that some ordinary demands may be experienced as disproportionately threatening to autonomy or control, although this proposed mechanism remains theoretical and requires further empirical evaluation. Building on this concept, researchers developed the 26-item parent-report Extreme Demand Avoidance Questionnaire (EDA-Q) in 2014 to measure PDA-related traits in children aged 5–17 [11]. This was later refined into an 8-item version (EDA-8) in 2021 [12]. Also using Newson’s original framing, other work has identified a PDA-related behavioral cluster within the Diagnostic Interview for Social and Communication Disorders (DISCO) [13], with higher scorers “characterized by lack of cooperation, use of apparently manipulative behavior, socially shocking behavior, difficulties with other people, anxiety, and sudden behavioral changes from loving to aggression.” [6]

Several PDA advocacy organizations have subsequently broadened Newson’s framing by shifting the focus from “ordinary” to “everyday” or “common” demands. For example, PDA North America describes PDA children and adults as individuals who “avoid everyday demands using ‘social strategies’ as part of the avoidance, have a strong need for autonomy and control, require unique supports and are socially oriented.” [14] The PDA Society in the United Kingdom similarly describes PDA as “a determined avoidance of so-called ‘common’ demands of life, including expectations and things the person enjoys doing.” [15] While these descriptions advance Newson’s original account by centering autonomy and emphasizing avoidance of even desired activities, they do not fully distinguish PDA from other behavioral profiles [16] or from “rational demand avoidance” [17] associated with autism and/or ADHD. Moreover, they do not specify a unique mechanism underlying perceptual or regulatory differences in PDA that is distinct from other pathways that can contribute to demand avoidance in neurodivergent individuals, such as executive functioning challenges [18,19] painful or threatening sensory input [19], monotropic focus [20–22], and/or anxiety [23].

Building on Newson’s original description and drawing on practitioner observations, lived-experience accounts [24-25], and Self-Determination Theory [26,27] which conceptualizes autonomy as a basic psychological need that supports self-motivation and healthy development, we developed an autonomy-centered, hypothesis-generating formulation of PDA. Because published empirical literature regarding the mechanisms underlying PDA remains limited, this formulation should be understood as a proposed model rather than an established explanation. We provisionally conceptualize PDA as a neurodivergent profile characterized by a pervasive drive for autonomy that may, in some individuals, include equalizing behaviors or efforts to restore a perceived sense of fairness, control, or relational balance. In this model, perceived threats to autonomy and associated distress may interfere with response to both external and internal demands as nervous system activation accumulates [28]. We further hypothesize that neuroception [29], the proposed process of detecting cues of safety, danger, or threat outside of conscious awareness, may contribute to cumulative nervous-system activation, progressively narrow an individual’s Window of Tolerance [30], and ultimately interfere with their ability to fulfill one or more basic needs (i.e., hygiene, eating, sleeping) or to toilet – either in the moment or over time. This model is intended to generate testable hypotheses about one possible pathway to demand avoidance, not to imply that autonomy threat is its sole cause or that these processes are unique to PDA. Demand avoidance may also be transactional and context-dependent, emerging through interactions among the individual, the nature and delivery of demands, prior experiences, relationships, and environmental responses. Other neurodevelopmental and clinical presentations (e.g., autism, attention deficit hyperactivity disorder, obsessive-compulsive disorder, bipolar disorder, oppositional defiant disorder, and trauma-related conditions) may also involve a strong drive for autonomy or demand avoidance arising through sensory, cognitive, emotional, interpersonal, or contextual pathways. The present cross-sectional study cannot determine when demand avoidance develops, whether it generalizes over time, or whether it represents a stable developmental trait.

Although recognition and discussion of PDA have increased in recent years among some practitioners and clinicians, in the emerging literature, and through online testimonies of adults with lived experiences of PDA [24,31,32], the validity of PDA as a distinct diagnosis remains widely debated [2]. For example, Woods [7] cautions that framing demand avoidance phenomena as inherently autistic risks a false equivalence, given the lack of consensus on defining features and the plausibility of alternative explanatory pathways (e.g., trauma and comorbidity interactions). While PDA is often described as a profile or subtype of autism, others conceptualize it as a distinct developmental disorder that is separate from autism or other neurotypes [33]. Still others suggest that PDA is better understood as a cluster of symptoms rather than a discrete condition [34]. In addition, autism researchers and autistic self-advocates have raised concerns that the concept of a PDA construct may undermine autistic agency and self-advocacy, arguing that many of the observed behaviors may instead reflect “rational” demand avoidance [10]. Similar critiques emphasize the risk of pathologizing resistance and highlight alternative explanatory frameworks (e.g., executive functioning, monotropism, autistic inertia, trauma-related processes) [22].

The limited scientific evidence supporting PDA’s existence has contributed to its exclusion from major diagnostic reference manuals, including the American *Diagnostic and Statistical Manual of Mental Disorders* (DSM; American Psychiatric Association) and the European *International Classification of Diseases* (ICD; World Health Organization) [35]. While this caution reflects the protective function of evidentiary standards for diagnostic categories and concerns about prematurely reifying a contested construct, it has also impeded progress in resolving ongoing controversy and has left many families of children with PDA-related traits feeling unseen and unable to access appropriate interventions and supports [36]. Consistent with these concerns, some authors have recommended formulating PDA-related presentations in terms of co-occurring or interacting difficulties rather than treating PDA as a discrete diagnostic entity [34]. The lack of PDA-related clinical guidance is especially problematic when providers default to treatment strategies designed for ASD or other neurodevelopmental presentations that may not address marked demand avoidance or autonomy-related distress; such strategies may be ineffective and may inadvertently intensify the fight-or-flight response commonly observed in these individuals with PDA.

A major barrier to progress in this area is the absence of a consensus approach to measuring PDA traits. To date, the only measure specifically designed to assess PDA traits is the Extreme Demand Avoidance Questionnaire (EDA-Q) and its abbreviated derivative, the EDA-8 [11,37,38] The original 26-item, parent-report EDA-Q (for parents of children ages 5–17) provided preliminary evidence for internal consistency, content validity, sensitivity, and specificity in the initial development work [37], but these psychometric properties have not been independently established. Conceptual and methodological concerns have also been raised, including potential ceiling effects at the more extreme end of demand avoidance, reliance on caregiver report (with attendant risks of common-rater and confirmation bias), and limited evidence that the measure reliably distinguishes PDA-like demand avoidance from demand avoidance observed in other clinical presentations.[2,7]

More recently, the developers of the EDA-Q used item response theory and differential item functioning analyses to refine the measure, resulting in an 8-item version (EDA-8) with evidence of good internal consistency, and convergent and discriminant validity. However, these findings also await independent replication, and key forms of validity (e.g., known groups validity, predictive validity, sensitivity and specificity) have not yet been established [38]. In addition, the IRT refinement sample (N = 334) is smaller than is often recommended for stable item parameter estimation [39]. Beyond these psychometric gaps, the EDA-8 has conceptual limitations: its brevity constrains content coverage of demand-avoidance behaviors, more internalized expressions of distress, and functional difficulties that are frequently reported by families (e.g., difficulties with hygiene, eating, sleeping, or toileting). Hygiene and toileting difficulties were not prominent features of Newson’s original account and are not systematically assessed in prior PDA instruments. Nevertheless, stakeholder input identified these as potentially important functional difficulties among some children with marked demand avoidance. They may have multiple developmental, medical, sensory, psychological, or contextual explanations and should not be assumed to be core or specific features of PDA. Rather, these domains represent clinically relevant areas of impairment that warrant systematic assessment. These are key gaps in measurement that we aim to address.

The ongoing debate about the PDA construct underscores the need for clearer operational definitions and measurement approaches that capture both behavioral expressions of demand avoidance and associated functional impacts [7].The purpose of this study was to develop the Demand Avoidance and Autonomy Questionnaire (DAAQ), a new caregiver-reported assessment designed to characterize demand-avoidance behaviors and potentially associated functional difficulties involving basic needs (e.g., hygiene, eating, sleeping, toileting) that emerged through stakeholder input. The DAAQ was developed using an autonomy-centered conceptual framework in which demands may be experienced as potentially threatening or overwhelming by some individuals. This framework does not assume that demands are objectively ordinary or benign, that all demand avoidance is caused by autonomy threat, or that autonomy is the only pathway to demand avoidance. Rather, the DAAQ operationalizes demand-avoidance-related traits as conceptualized in the present model, with autonomy serving as the primary theoretical lens.

We followed a rigorous development process in accordance with established measurement development standards [40]. Specifically, we applied both classical test theory and item response theory methods, and generated item content using input from key stakeholders (parents of PDA children/adolescents, adults with lived experience of PDA, treatment providers, and measurement development experts), as well as responses to a preliminary questionnaire that was completed by a purposeful sample of 15 parents of PDA children or teens between the ages of 3 and 17 on January 6^th^, 2022. Finally, we used an item-banking approach, calibrating items to a common latent trait metric, which creates a foundation for future refinement (e.g., adding, revising, or selecting subsets of items; developing short forms or computerized adaptive testing) while preserving score comparability over time through linking to the same underlying scale [41].

## Methods

### Study participants

This sample included 707 self-identified parents or legal guardians of children aged 4–17 with a suspected PDA profile. Respondents had to be at least 18 years old and be able to read/comprehend English. The term ‘suspected PDA profile’ reflected the terminology used in recruitment and by the community from which the sample was drawn. Its use should not be interpreted as establishing PDA as a validated diagnostic profile or as resolving competing conceptualizations of demand avoidance. Participants were recruited via email using a list of subscribers to At Peace Parents, a PDA parenting support listserv. An announcement for the study, along with a link to the survey, was delivered on November 6^th^, 2023.

### Study measures

#### The DAAQ item pool.

An initial pool of 67 questions was designed to evaluate the hypothesized key behavioral traits of PDA, including demand avoidance, autonomy-related distress, equalizing behavior, masking behavior (two versions of oneself), cumulative nervous system activation (fluctuating and unpredictable), and need for constant 1:1 undivided attention. Specific item content was based on literature review, lived experience, experience working with families with PDA, and surveys that were conducted with 15 parents of PDA children or teens. These data were used to inform the conceptual framework upon which this measure was designed (Fig 1). Items were further refined by expert review (n = 3 PhD-level scientists; 1 MA-level investigator; and n = 2 BA-level professionals), evaluation of item literacy level (to ensure ≤6^th^ grade reading level) [42], translatability review (to facilitate a future translation into Spanish), and participant cognitive review (n = 5) to ensure adequate content coverage and appropriate reading and comprehension levels. The final item pool was comprised of 60 items (Fig 2).

**Fig 1:**
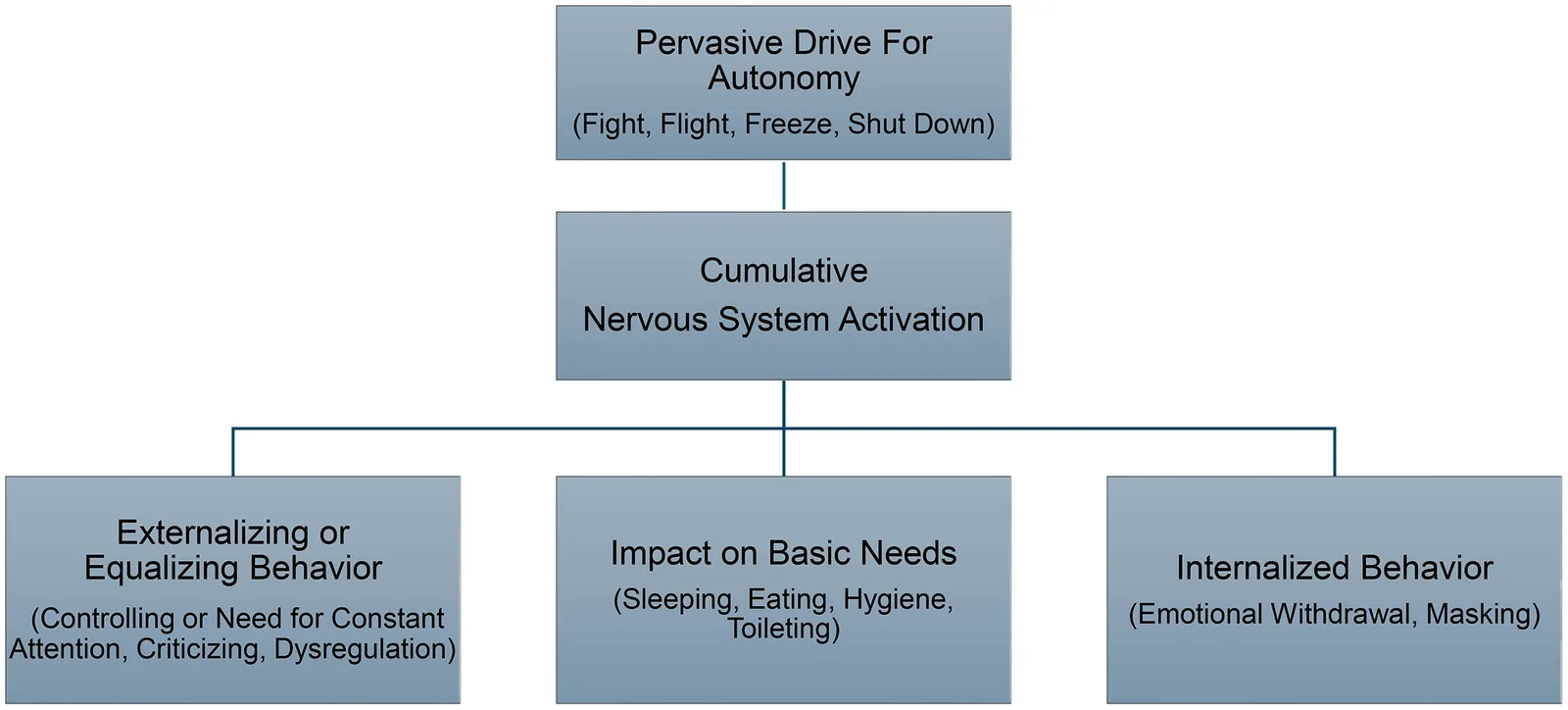
Conceptual Model. A survival-driven need for autonomy—expressed through fight, flight, freeze, or shutdown responses—contributes to cumulative nervous system activation. This activation may manifest as equalizing behaviors (e.g., controlling behavior, need for constant attention, criticism, or dysregulation), disruptions in basic needs (e.g., sleeping, eating, hygiene, or toileting), and internalized behaviors (e.g., emotional withdrawal or masking).

**Fig 2:**
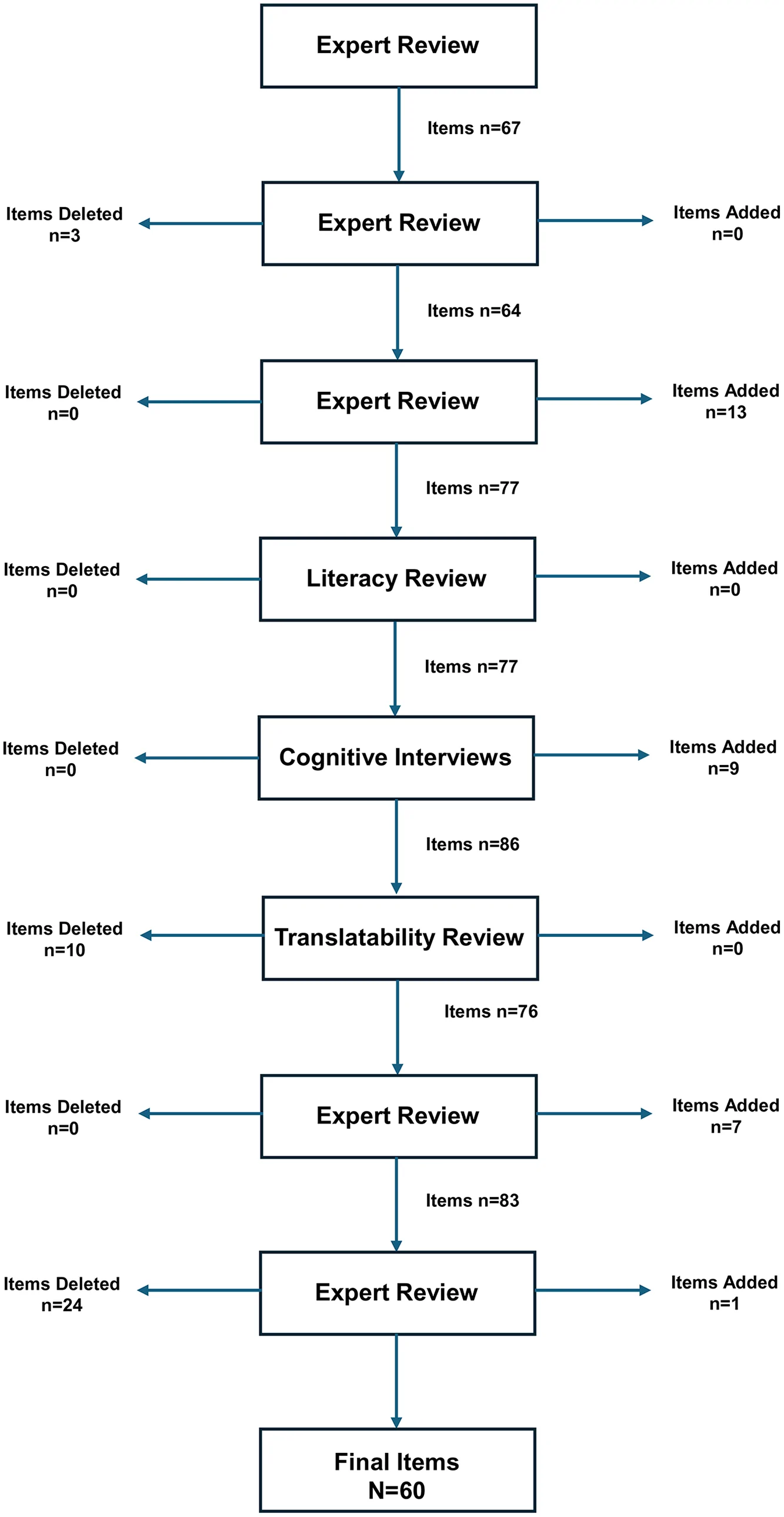
Item History. Candidate items underwent iterative expert review, literacy review, cognitive interviews, and translatability review. Items were added or deleted at each stage based on reviewer and participant feedback, resulting in a final 60-item measure. Numbers between stages indicate the total number of items entering the subsequent review stage; lateral annotations indicate the number added or deleted during each stage.

Participants also completed: 1) **EDA-8** [37,38], an 8-item parent proxy measure designed to assess the presence of PDA-related traits in children; 2) **Childhood Autism Spectrum Test** (CAST) [43], a 39-item parent proxy measure designed to assess the severity of autism symptoms in children (we note that individuals with high demand avoidance may endorse autism-screening items for reasons that differ from autistic individuals, which can complicate interpretation of associations with CAST scores [37]; 3) **Strengths and Difficulties Questionnaire** (SDQ) [44], a 25-item proxy-reported child behavioral screening questionnaire, 4) **PROMIS Parent Proxy Scale v1.0 – Global Health 7 + 2** [45], a 9-item parent proxy measure designed to assess a child’s physical, mental, and social health; and 5) demographic questions about themselves and the PDA child or teen.

### Study procedures

The local Institutional Review Board (IRB) determined that this study was exempt from IRB oversight. Participants completed surveys online via a study-specific Research Electronic Data Capture (REDCap) [46] web link. Informed consent was obtained online via the REDCap platform. Interested parents of children with a suspected PDA profile who responded to the study recruitment link were taken to a REDCap webpage and presented with the consent form. This page provided participants with information about the study, including the potential risks, benefits, and the voluntary nature of the survey. Participants were asked to contact the study team if they had questions, and the study team’s contact information was provided. Because the study was granted exemption from IRB review, formal written consent documentation was not required, however this consent page advised participants that if they would like to continue to the survey, to click a “submit” button at the bottom of the webpage. Participants who indicated their consent to participate in the study in this manner were taken to the first page of the survey. The date of consent was retained for documentation, but no identifying information was collected.

### Analysis plan

#### Survey development.

Analyses for the development of the DAAQ were conducted according to established standards and involved both classical test theory (CTT) and item response theory (IRT) [40]. We first evaluated the dimensionality of the item pool. The sample was randomly divided into two datasets: one for exploratory factor analysis (EFA) and the other for confirmatory factor analysis (CFA). EFA was used to explore potential factor structure among items. We explored the number of factors based on number of factors with eigenvalues > 1 and the scree plot (i.e., number of factors before the break in scree plot) [47]. Items with primary factor loadings of ≥0.30 were considered candidates for retention, particularly when they were conceptually important and supported by subsequent psychometric evaluation. CFA was conducted using the weighted least square-mean and variance adjusted estimator with polychoric correlations [48]. Item sets were considered unidimensional, with their items retained in the item pool, when the following criteria were met: comparative fit index (CFI) >0.9; root mean square error of approximation (RMSEA <0.15 [49]; R2 > 0.3; correlated error modification index <10; residual correlations <0.15 [50]). Samejima’s graded response model (GRM) was used to identify items with significant misfit (S-X2/ df effect size >3) [51] using IRTPRO (version 3.1.2) [52]. Items with weak primary loadings, cross-loadings, or poor performance in subsequent CFA/IRT analyses were considered for removal.

Differential item functioning (DIF) was investigated to identify potential item bias (e.g., to identify items that might unfairly discriminate for or against individuals in a given group) [53] using R package LORDIF Version 0.3–2 [54]. DIF was evaluated by child age, education, school type, and sex; and parent age and education. Items with DIF across more than one comparison (non-negligible DIF criterion: R^2^ > 0.016 and p < .01) were candidates for exclusion. Samejima’s graded response model was also used to establish the item parameters of the final item banks [55]. Scoring for the finalized item banks involved the conversion of theta scores to a standardized *t* score (mean = 50, SD = 10).

#### Preliminary reliability and validity data.

We estimated IRT-based reliabilities at individual *t* score levels, which is derived from standard error estimates (i.e., reciprocal of test information). IRT-based reliabilities vary across the measurement continuum. A reliability ≥0.70 was considered acceptable [56]. Convergent validity was evaluated by examining the correlations between the DAAQ scores and the EDA-8 (criterion *r*’s ≥0.36) [57] and discriminant validity was evaluated by examining the correlations between the DAAQ item banks and measures that should be largely unrelated (i.e., autism traits subscales from the SDQ and child global health; criterion <0.36) [57]. Known groups validity was examined by comparing groups that are expected to show differences on the DAAQ. Specifically, we expected those with more uncommon behaviors (relative to those with more common behaviors; SDQ scores >17 versus ≤17), those with high demand avoidance (relative to those with low demand avoidance; EDA-8 scores >19 versus ≤19), those with a co-occurring autism diagnosis (relative to those without), those who were unschooled (relative to those who were homeschooled, in public school, or in private school), and those with basic needs difficulties (relative to the sample mean), to have worse DAAQ scores than the specified comparator group; we also expected these groups to have elevated clinical impairment rates (i.e., scores that were >1 SD worse than the normative sample mean of 50) [58].

#### Sample size considerations.

Sample sizes of 200–1000 have been proposed when using GRM, with larger sample sizes yielding more stable item parameter estimates [59,60]. In addition, a minimum of 5 individuals is recommended for each item within an item pool [61–63]; with 60 items in our pool, a minimum of 300 participants would be needed. Furthermore, DIF analyses can be performed provided the overall sample size is ≥ 500 and there are at least 200 participants within each variable category tested [64].

## Results

A total of 707 parents of PDA children or teens participated in this study; see Table 1 for details.

**Table 1:** Sample characteristics (n = 707).

Variable	n (%)/ M (SD)
Parent Age	41.9 (6.9)
Parent Sex	
Female	681 (96)
Male	16 (2)
Genderqueer/gender nonconforming/transgender	7 (1)
Do Not Wish to Report	3 (1)
Parent Race	
More than one race	16 (2)
Asian	10 (1)
American Indian or Alaska Native	1 (0)
Black or African American	3 (0)
White or Caucasian	644 (91)
Other	13 (2)
Do not wish to report	20 (3)
*Parent Ethnicity (%)*	
Non-Hispanic	629 (89)
Hispanic	36 (5)
Do not wish to report	42 (6)
*Parent Marital Status (%)*	
Married or Cohabitating	597 (84)
Separated	18 (3)
Single & divorced	45 (6)
Single & never married	32 (5)
Single & widowed	5 (1)
Other	2 (0)
Do not wish to report	8 (1)
Country	
United States	445 (63)
United Kingdom	115 (16)
Germany	9 (1)
Ireland	14 (2)
Australia	26 (4)
Canada	58 (8)
Denmark	7 (1)
South Africa	7 (1)
Other	26 (4)
*Education*	
Grades 9–12, without graduating	4 (1)
High school graduate or GED	28 (4)
Some college credit or 2-year degree	123 (17)
4-year college degree	237 (34)
Master’s degree (MA, MS, MEng, MEd, MBA)	238 (34)
Professional degree (MD, DDS, DVM, LLB, JD)	45 (6)
Doctoral degree (PhD, EdD)	28 (4)
Do not wish to report	4 (1)
*Work Status (%)*	
Full-time (35–40 hours per week)	236 (33)
Part-time	192 (27)
Homemaker	157 (22)
Student	7 (1)
Unpaid family caregiver	64 (9)
Disabled	12 (2)
Unemployed	13 (2)
Retired	5 (1)
Other	15 (2)
Do not wish to report	6 (1)
*How your finances usually work out at the end of the month*	
I do not have enough money to make ends meet	109 (15)
I have enough money to make ends meet	324 (46)
I have money left over	244 (35)
Do not wish to report	30 (5)
*Parent Co-occurring conditions*	
Autism Spectrum Disorder	65 (9)
ADHD	187 (26)
Major Depressive Disorder	121 (17)
Bipolar Disorder	14 (2)
Generalized Anxiety Disorder	244 (35)
Obsessive Compulsive Disorder	31 (4)
Panic Disorder	20 (3)
Pathological Demand Avoidance	14 (2)
None of the above or do not wish to report	286 (40)
*PDA Child or Teen’s Grade in School*	
Never attended school	28 (4)
Preschool	87 (12)
Kindergarten	95 (13)
1st grade	93 (13)
2nd grade	71 (10)
3rd grade	79 (11)
4th grade	56 (8)
5th grade	54 (8)
6th grade	32 (5)
7th grade	33 (5)
8th grade	28 (4)
9th grade	16 (2)
10th grade	13 (2)
11th grade	6 (1)
12th grade, no diploma	2 (0)
High school graduate	2 (0)
Do not wish to report	12 (2)
*School Type*	
Public school	397 (56)
Private school	99 (14)
Homeschooling with curriculum	52 (7)
Unschooling (child-led or Self-Directed Education)	156 (22)
Do not wish to report	3 (1)
*Developmental delays for PDA child*	
Motor milestones	139 (20)
Language milestones	191 (27)
Cognitive milestones	99 (14)
Social delays	232 (33)
No known delays	314 (44)
Unknown/Not sure	29 (4)
*Age of PDA child or teen (years)*	8.8 (3.3) range 4–17
*Sex of PDA child or teen*	
Female	255 (36)
Male	449 (64)
Do not wish to report	3 (0)
*Child PDA Diagnosis*	
PDA (clinical diagnosis)*	119 (17)
PDA (no clinical diagnosis)*	523 (74)
Suspected PDA	65 (9)
*Child Co-Occurring Diagnoses*	
Attention Deficit Hyperactivity Disorder	318 (45)
Autism Spectrum Disorder	397 (56)
Obsessive Compulsive Disorder	37 (5)
Generalized Anxiety Disorder	267 (38)
Major Depressive Disorder	42 (6)
Disruptive Mood Dysregulation Disorder	37 (5)
Intermittent Explosive Disorder	12 (2)
Oppositional Defiant Disorder	68 (10)
PDA Expression	
Mostly Fight/Flight	392 (55)
Mostly Freeze	38 (5)
Mix of Fight Flight and Freeze	273 (39)
Unsure	4 (1)
Basic Needs Impact	
Eating	388 (55)
Sleeping	439 (62)
Toileting or diaper changes	266 (38)
Hygiene (bathing, brushing their teeth, brushing their hair or clipping their nails)	538 (76)
School	524 (74)
None of the above	27 (4)
I have sought professional help for my child’s problems	
Eating	80 (21)
Sleeping	61 (14)
Toileting	62 (23)
Hygiene	217 (41)

Note: * Given that this was an international study and that PDA is only recognized as a diagnosis in a limited number of countries, parents could either indicate whether or not their child was diagnosed by a professional

### DAAQ survey development

Initial EFA results suggested five factors (S1 Table). Factor 1 included 25 items that covered behavioral traits of PDA (pervasive drive for autonomy, equalizing, externalized or “fight” behavior; one item cross-loaded on Factor 2 and one item cross-loaded on Factor 4). Factor 2 included 19 items that were focused on basic needs (hygiene, eating, sleeping); one item cross-loaded on Factor 1). Factor 3 included 6 toileting items. Factor 4 included two sleep items, and two items focused on the cumulative impact of nervous system activation (one of which cross-loaded on Factor 1); given the small number of items and the fact that demand avoidance for sleep was also conceptually covered by Factor 2, this factor was not given additional consideration. Factor 5 only included 3 items that focused on a fight response; given that 3 items is insufficient for a scale, no additional consideration was given to this factor.

We conducted a second EFA including only the items from the initial EFA that loaded on either Factors 1, 2, or 3. This second EFA (see Table 2) had: 1) 25 items for Factor 1 (“Behavioral Expression”; again covering externalized behavior, demand avoidance, and co-regulation); 2) 18 items for Factor 2 (“Basic Needs”; one of which had a higher cross-loading on Factor 1); and 3) 6 items for Factor 3 (“Toileting”). None of the remaining items were flagged for DIF for child schooling type, parent age, or parent education. Although 4 items were flagged for potential DIF for child age, these items were retained given that they did not also get flagged based on other demographic variables. A summary of this iterative process is shown in S2 Table.

**Table 2:** Exploratory Factor Analysis for a 3 Factor Solution (Rotated Factor Pattern).

Item	Factor 1: Behavioral Expression	Factor 2: Basic Needs	Factor 3: Toileting
When playing games, my child becomes distressed (dysregulated/agitated) if they are not winning.	0.55		
When playing games, my child attempts to change the rules of the game, cheat, or control other people’s behavior in order to win.	0.66		
My child has difficulty with transitions to a preferred activity.	0.34		
If I try to encourage my child to participate in activities they dislike, my child will become distressed (dysregulated/agitated) and/or have a meltdown (panic attack/temper tantrum).	0.43		
If I try to incentivize (sticker charts, rewards) my child to participate in activities they dislike, my child will become distressed (dysregulated/agitated) and/or have a meltdown (panic attack/temper tantrum).	0.33	0.31	
When my child is in a situation they find stressful, my child shows flight behavior (running or climbing on something to get away, crawling underneath something, hiding).	0.36		
When my child is in a situation they find stressful, my child behaves inappropriately (laughs or says something inappropriate, removes their clothes, makes inappropriate sounds or noises, uses nonsense words, repeats words).	0.54		
When my child is in a situation they find stressful, my child shows fight behavior (kicking, hitting, biting, screaming, swearing, hissing, growling, throwing things, knocking things over, destroying things).	0.59		
When my child is in a situation they find stressful, my child shows freeze behavior (withdrawing, hiding, shutting down, disengaging, entire body going limp, not speaking/nonresponsive, blank stare or glazed eyes, not moving/saying they can’t move or their		0.33	
My child becomes distressed (dysregulated/agitated) when they are told that they have to stop doing a preferred activity (e.g., screen time).	0.43		
My child will refuse help, even after they have asked for it.	0.36		
When my child is told to do something, they will comply but do things in a different or more difficult way.	0.34		
When my child is told to do something, they will say, I can’t, I don’t feel well, ignore the request, act as if they do not understand (change the topic, pretend not to hear, start doing something unrelated), pretend to be an animal, or leave the room.	0.35		
My child destroys their own things.	0.53		
When my child is excited about something (e.g., a holiday), they become distressed (dysregulated/agitated).	0.38		
My child will show excitement about something only to refuse when it is time to participate (leave the house, go into building/event).	0.43		
My child will respond to being told No, Stop or Don’t by doing the opposite, even if it is dangerous (runs towards the street, touches a hot stove, climbs a high ledge).	0.64		
When my child does not get their way, they will respond by knocking something down, shouting, calling names, needing to have the last word, or destroying something.	0.65		
My child uses self-harm (cutting, picking, self-hitting, hair pulling, skin picking, running into things, doing things that are dangerous) when they are having a meltdown (panic attack/temper tantrum) or are distressed (dysregulated/agitated).		0.30	
My child likes to be physically above people (e.g., likes to sit or stand above other people, stand on chairs or the couch, climb onto windowsills, sit on the back of the couch).	0.47		
My child breaks/ignores rules but gets upset if others do not follow them.	0.53		
When I ask my child a direct question, they respond with a growl or hissing sound, an I don’t know, stop talking, silence, or a silly word like poop.	0.48		
When I tell my child to do something, they respond with a growl or hissing sound, an I don’t know, stop talking, silence, or a silly word like poop.	0.50		
My child needs constant attention, or they will become distressed (dysregulated/agitated) or have a meltdown (panic attack/temper tantrum).	0.48		
Unless my child is participating in a preferred activity (e.g., on a screen or a special interest), they become distressed (dysregulated/agitated) or have a meltdown (panic attack/temper tantrum) when I try to talk to another person.	0.59		
Unless my child is participating in a preferred activity (e.g., on a screen, or a special interest), or with someone they trust, he/she will become distressed (dysregulated/agitated) or have a meltdown (panic attack/temper tantrum) if I leave.	0.35		
My child wants me to stay nearby but does not want me to touch or interact with them.	0.38		
My child will only participate in fun activities (e.g., play/sports, drawing, or building) if someone they trust is also participating in the activity.		0.33	
My child focuses on one or two preferred topics/activities.		0.32	
My child is fine (behaves appropriately) in settings outside of the home (e.g., school/daycare, a grandparent’s house) but wants to be alone when they return home.		0.32	
My child needs to be in control of when, where and/or what they eat.		0.60	
My child will only eat preferred foods.		0.49	
My child refuses to eat food at a table.		0.36	
My child resists sleeping.		0.49	
My child needs to be in control of when and/or where they sleep.		0.66	
My child has an irregular sleep cycle (e.g., they go to sleep at different times and wake up at different times each night/day).		0.50	
My child resists going to the bathroom.			0.87
My child needs to be in control of when and/or where they go to the bathroom (or have diaper changes).			0.70
My child has lost skills related to toileting (needing to use a diaper again, toileting outside or in a particular location like the bathtub, or refusing all together).			0.79
My child has toileting accidents.			0.75
When my child becomes distressed (dysregulated/agitated), their ability to toilet independently decreases (wipe themselves, use the toilet alone).			0.76
My child withholds his/her urine (pee) or stool (poop).			0.78
My child resists bathing or showering.		0.60	
My child needs to be in control of when and/or where and/or how they bathe or shower.		0.82	
My child resists brushing their teeth.		0.60	
My child needs to be in control of when and/or where and/or how they brush their teeth.		0.81	
My child resists brushing their hair.		0.56	
My child needs to be in control of when and/or where and/or how they brush their hair.		0.78	

Several items in the EFA had primary loadings near the lower bound of the prespecified exploratory retention criterion (i.e., 0.30), and one item showed a notable cross-loading. Specifically, the item regarding use of incentives to participate in disliked activities loaded on Behavioral Expression and cross-loaded on Basic Needs. Items with modest EFA loadings were retained if they were conceptually relevant.

CFAs were then used to confirm the essential unidimensionality of the remaining 20 Factor-1, 10 Factor-2, and 6 Factor-3 items, respectively (Table 3). Next, IRT parameter estimates for the 20 items in Factor 1 (Behavioral Expression) indicated slopes ranging from 0.88 to 1.86 and thresholds ranging from −6.04 to +3.24; the 10 items in Factor 2 (Basic Needs) indicated slopes ranging from 0.92 to 2.61 and thresholds ranging from −5.04 to 2.46; and the 6 items in Factor 3 (Toileting) indicated slopes ranging from 1.82 to 3.29 and thresholds ranging from −1.86 to 2.52 (S3 Table). Scores were converted to a *t* score (mean = 50, SD = 10) relative to the sample means. The final measure and scoring instructions are provided in the appendix.

**Table 3:** Final Model Fit for the DAAQ and initial item performance.

Factor	T Scores M (SD)	CFI (criterion > .90)	TLI (criterion > .90)	RMSEA (criterion < .15)	Internal Consistency (criterion > .70)	Floor %	Ceiling %
Factor 1: Behavioral Expression	50.0 (9.4)	0.95	0.93	0.06	0.87	0.0	0.1
Factor 2: Basic Needs	50.0 (9.3)	0.96	0.93	0.109	0.84	0.0	0.0
Factor 3: Toileting	50.0 (9.4)	0.97	0.96	0.156	0.87	4.1	1.0

*Note.* Chi-square = chi-square test of model fit, CFI = Comparative Fit Index, TLI = Tucker-Lewis Index, RMSEA: Root Mean Square Error of Approximation.

Preliminary Reliability and Validity data. Based on expected IRT-based reliability, precise estimation (defined as reliability ≥ 0.7) could be produced between T-scores 20 and 80 for Factor 1 (Behavioral Expression), between 20 and 67 for Factor 2 (Basic Needs), and between 30 and 77 for Factor 3 (Toileting; see Fig 3 for the IRT-based reliability). The marginal reliabilities were 0.89, 0.86, and 0.89, for Factor 1, 2, and 3, respectively. Furthermore, the interrelationships among the DAAQ item bank scores and comparator measures were all consistent with the proposed hypotheses: there were moderate relationships between the DAAQ and EDA-8 scores supporting convergent validity, and there were generally negligible to small correlations between the DAAQ scores and the other prespecified discriminant validity comparators (Table 4); *note:* there were two exceptions for Behavioral Expression where there were low-moderate correlations with the SDQ Hyperactivity and Conduct Problems subscales. In addition, all groups expected to have higher (i.e., worse) DAAQ scores did (Table 5) with two exceptions (there was no difference for those who were in private school versus those who were unschooled for Toileting scores, as well as for those with less versus more uncommon behaviors for Toileting; both of these findings were in the expected direction, although they did not meet conventional levels of significance).

**Table 4:** Convergent and Discriminant Validity.

HRQOL Measure		Discriminant Validity							
	Convergent Validity EDA-8	*SDQ Prosocial Scale*	*SDQ Peer Problems Scale*	*SDQ Hyperactivity Scale*	*SDQ Conduct Problems*	*SDQ Externalizing*	*SDQ Emotional Problems*	*Global Health*	*CAST*
Factor 1: Behavioral Expression	0.64	−0.22	0.18	0.26	0.43	0.41	0.30	−0.15	0.32
Factor 2: Basic Needs	0.44	−0.09	0.21	0.09	0.25	0.19	0.35	−0.32	0.21
Factor 3: Toileting	0.24	−0.04	0.04	0.12	0.18	0.18	0.13	−0.07	0.16

**Table 5:** Known Groups Validity.

DAAQ Factor		Less uncommon behaviors (SDQ < 17) (n = 58)		More uncommon behaviors (SDQ ≥ 17) (n = 576)		*t*	*p*	*d*
		Mean (SD)	*%* *Impaired (>60)*	Mean (SD)	*%* *Impaired (>60)*			
Factor 1: Behavioral Expression		42.6 (7.0)	0.0	50.9 (9.1)	13.7	8.34	<0.0001	0.89
Factor 2: Basic Needs		43.5 (9.2)	6.9	50.8 (9.1)	16.2	5.82	<0.0001	0.78
Factor 3: Toileting		48.0 (10.9)	12.7	50.4 (9.3)	14.9	1.82	0.07	0.25
		Low demand avoidance (EDAQ ≤ 19) (n = 395)		High demand avoidance (EDAQ > 19) (n = 290)				
Factor 1: Behavioral Expression		46.2 (8.1)	3.3	55.4 (8.5)	24.5	14.2	<0.0001	0.97
Factor 2: Basic Needs		47.2 (8.7)	7.9	54.0 (8.7)	24.1	10.1	<0.0001	0.73
Factor 3: Toileting		48.6 (8.8)	7.6	52.1 (9.8)	22.8	4.92	<0.0001	0.38
		Co-occurring Diagnosis of Autism = No (n = 61)		Co-occurring Diagnosis of Autism = Yes (n = 397)				
Factor 1: Behavioral Expression		46.2 (8.0)	1.6	51.1 (9.6)	15.6	3.74	<0.0001	0.52
Factor 2: Basic Needs		45.4 (9.1)	3.3	51.6 (9.0)	19.4	4.98	<0.0001	0.67
Factor 3: Toileting		46.9 (9.4)	6.6	50.7 (9.5)	16.6	2.88	0.004	0.40
		Schooling		Unschooling (n = 156)				
Factor 1: Behavioral Expression	Public School (n = 397)	49.2 (9.0)	9.3	52.5 (9.9)	20.5	3.7	.0002	0.35
	Private School (n = 99)	49.6 (9.4)	13.1			2.30	0.02	0.30
	Home School (n = 52)	49.1 (10.6)	9.6			2.13	0.03	0.33
Factor 2: Basic Needs	Public School (n = 397)	48.6 (9.0)	10.3	54.5 (8.7)	28.9	6.91	<.0001	0.64
	Private School (n = 99)	48.9 (9.6)	10.1			4.77	<0.001	0.59
	Home School (n = 52)	49.4 (9.8)	13.5			3.63	0.0004	0.57
Factor 3: Toileting	Public School (n = 397)	49.5 (9.3)	11.8	51.7 (8.9)	17.3	2.58	0.01	0.24
	Private School (n = 99)	50.1 (9.9)	14.1			1.35	0.18	0.17
	Home School (n = 52)	47.8 (9.8)	13.5			2.71	0.007	0.42
		Basic Needs Difficulty = No (n = 29)		Basic Needs Difficulty = Yes (n = 678)				
Factor 1: Behavioral Expression		44.5 (7.1)	0	50.2 (9.5)	12.8	3.22	0.001	0.61
Factor 2: Basic Needs		39.8 (8.2)	0	50.4 (9.0)	15.2	6.23	<0.0001	1.40
Factor 3: Toileting		32.5 (7.4)	0	50.3 (9.4)	14.3	3.83	0.0001	0.72

*Note.* SDQ = Strengths and Difficulties Questionnaire; EDAQ = Extreme Demand Avoidance Questionnaire

**Fig 3:**
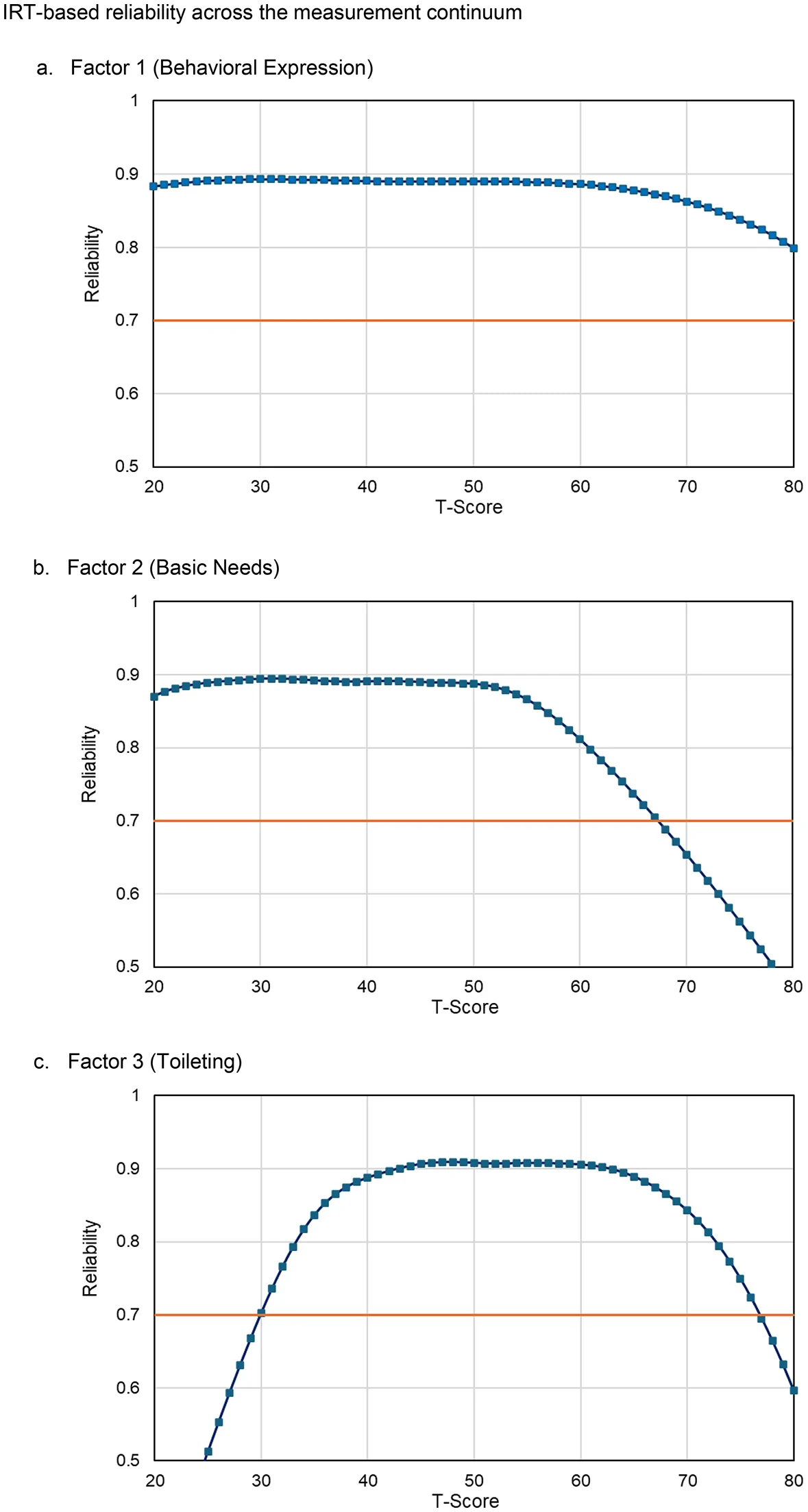
IRT-based reliability across the measurement continuum. X-axis represents the measurement continuum (in T-scores) and Y-axis represents the IRT-based reliability. The blue line represents the reliabilities across the continuum. The red line represents the reliability of 0.7, serving as a cut-off to show the range where precise estimates are expected to occur.

## Discussion

This paper describes the development of the DAAQ, a new 36-item measure of PDA-related behaviors and functional impacts on basic needs. It also provides preliminary reliability and validity data to support its clinical utility. This measure was developed in accordance with accepted measurement development standards, which included both CTT and IRT analytical approaches. Specifically, factor analyses supported three unidimensional sets of items that were devoid of item bias for child schooling, child sex, parent age, and parent education. In addition, psychometric analyses indicated that factor scores derived from the DAAQ exhibited very good internal consistency, were devoid of ceiling effects, and demonstrated convergent and discriminant validity as well as known groups validity.

The DAAQ includes three factors: Behavioral Expression, Basic Needs, and Toileting, all of which are scored on a *t* metric (*M* = 50, *SD* = 10), such that higher scores on each factor indicate more PDA-related behaviors and impacts on basic needs. Given that *t* scores are standardized, this type of scoring approach aids in clinical interpretation. For example, parents of children or teens with scores of 60 or above (those with scores that are ≥ one standard deviation above the mean) on any of the factors are reporting that their child has more significant behavioral expression, basic needs impact, or toileting impact (for Behavioral Expression, Basic Needs, or Toileting factors, respectively) that are worse than 83.9% of the total PDA children or teens in the sample. Furthermore, parents of PDA children or teens with scores of 70 or more (those with scores that are ≥ two standard deviations above the mean) are reporting behaviors, and basic needs, and toileting impacts that exceed 97.9% of their PDA-specific peers.

Convergent validity and discriminant validity for the three factors were supported. As expected, correlations were strongest between each DAAQ factor and the EDA-8, and weaker (generally negligible to small) with the discriminant validity measures. Two findings merit comment. First, Behavioral Expression correlated somewhat more strongly than hypothesized with hyperactivity and conduct problems (low-to-moderate correlations rather than negligible-to-low), which is unsurprising given item content overlap. This finding is consistent with prior PDA/demand-avoidance literature noting overlap with externalizing, oppositional, hyperactivity/inattention, and broader emotional/behavioral difficulties [33]. Second, although the Toileting factor correlated more strongly with the EDA-8 than with the other discriminant validity measures, the association was weaker than expected (i.e., less than 0.36). Consistent with this, inter-factor correlations indicated that Toileting was less strongly related to Behavioral Expression and Basic Needs (r = 0.36 and r = 0.24, respectively) than Behavioral Expression was to Basic Needs (r = 0.49), suggesting that toileting impact may reflect a partially distinct domain within the broader demand-avoidance profile assessed by the DAAQ.

Associations with autism screening measures also warrant caution. Although PDA is often discussed in relation to autism, and DAAQ factors showed small-to-moderate correlations with CAST scores, elevated demand avoidance may lead to endorsement of autism-screening items because of overlapping observable behaviors that reflect different motivations or mechanisms than those typically assumed in autism screening contexts. O’Nions et al. [37] similarly noted that individuals with marked demand avoidance may score comparably to autistic individuals on autism measures, but potentially for different reasons. Accordingly, CAST association should not be interpreted as the DAAQ measures autism severity or as confirmation that demand avoidance is necessarily an autism subtype/profile. Future studies should use clinician-characterized comparison groups and multi-method assessment (including analyses of item-level response patterns and differential item functioning) to separate behavioral overlap from underlying mechanisms.

Known groups validity was also supported; the DAAQ factor differentiated groups as hypothesized. Scores differed between children with more versus less frequent target behaviors (with a trend-level effect for Toileting, but with means in the expected direction), high versus low demand avoidance, and curriculum-based school versus unschooling. All three factors differentiated children with versus without co-occurring autism diagnoses (higher scores with autism) and those with and without basic needs difficulties (higher scores with reported difficulties). Notably, children with co-occurring autism had higher scores on all three DAAQ factors, but the reasons for this pattern are unclear and likely multifactorial. Higher scores may reflect a demand-avoidance profile that is more common within autism, a transdiagnostic dimension that is amplified by sensory, executive functioning, emotion regulation, and/or tolerance of uncertainty differences, and/or environmental and cultural contexts that are poorly adapted to autistic children’s sensory, communication, and autonomy needs. Unmet support, stigma, and trauma- or minority-stress-related processes may also contribute. In addition, because PDA has often been conceptualized, identified, and studied in relation to autism, autism-centered models may have influenced caregiver recognition of PDA and the item-development context. Therefore, higher DAAQ scores among children with co-occurring autism should not be interpreted as evidence that the DAAQ measures autism severity or that PDA is necessarily an autism subtype/profile. Clarifying these possibilities will require clinician-characterized comparison groups (e.g., autism without high demand avoidance; ADHD/anxiety with and without demand avoidance), multi-informant data, and longitudinal designs.

Taken together, the measurement development approach of the DAAQ, the preliminary reliability and validity of the scores from the DAAQ, and the inclusion of a general PDA behavior factor and basic needs and toileting impact factors, are strengths of the current measure. Regardless, there are several limitations to consider. Items intended to capture a more internalized expression of PDA did not survive the analysis, indicating a need for further development in this area. More broadly, proxy reports are limited for internal states that are difficult to observe, suggesting that these constructs may be better assessed via self-report in future work. In addition, the DAAQ is a parent-report measure; development of a child self-report version (ages 8+) is needed. Although the DAAQ was developed using an autonomy-centered model that included equalizing behaviors as potentially relevant to PDA-related presentations, the final factor structure did not support a distinct equality/equalizing domain. Accordingly, the current measure should be understood as primarily assessing demand-avoidance behavioral expression and basic-needs impact through an autonomy-centered lens, rather than as a comprehensive measure of autonomy and equality as separate constructs. In addition, demand avoidance may be transactional and context-dependent, and the present cross-sectional study cannot establish its etiology, age of onset, generalization, or developmental stability.

There is no consensus definition or identification approach for PDA. This contributes to heterogeneity in who is labeled (or self-identifies) as having PDA and to concerns about potential over-identification in some contexts [2]. The recruitment term “suspected PDA profile” may also have shaped who elected to participate and how respondents interpreted and reported their children’s behavior. As a result, our recruitment and item-development process may not capture experiences of individuals who frame their demand avoidance differently (e.g., as rational demand avoidance) or who do not identify with autonomy-focused conceptualizations. Relatedly, parents of children with stronger demand avoidance may be less likely to participate in questionnaire-based research, potentially biasing the item pool and calibration sample. Accordingly, the DAAQ should be interpreted as assessing demand-avoidance-related traits as operationalized here, rather than as a definitive measure of a universally agreed-upon PDA construct, and should be used alongside careful clinical formulation and attention to context and meaning-making. In addition, autonomy-centered models of PDA may represent a rhetorical and conceptual reframing of PDA from a descriptive behavioral profile toward a more explanatory formulation; this shift requires further empirical evaluation and should not be treated as settled by the current study. Because PDA is not a recognized diagnostic category in the United States, we could not evaluate sensitivity/specificity in this largely U.S.-based sample, and we necessarily relied on parent endorsement of a PDA profile rather than provider-confirmed diagnoses.

An additional limitation concerns the comparator measure: we used the brief EDA-8 rather than the full EDA-Q. Although the EDA-8 reduces respondent burden, it provides narrower content coverage and may be less sensitive to clinically meaningful heterogeneity, which could attenuate associations with external variables and constrain inferences about convergent validity. Replication using the full EDA-Q (and/or additional demand-avoidance measures) is needed.

Generalizability is also limited by sample characteristics; participants were highly educated and had greater socioeconomic means than the general population. Over half of parents reported an anxiety disorder, which may influence caregiver perceptions and questionnaire responding (and could contribute to rater effects); elevated parent stress has also been noted in prior work [65]. Future studies should examine the impact of caregiver mental health using multi-informant designs and tests of invariance/DIF by caregiver mental health status. Because participation was anonymous and the parent report was the sole data source, we could not verify child behaviors via independent informants or clinical assessment.

Furthermore, nearly all respondents were female, which is consistent with many caregiver-report studies [66], but limits our ability to determine whether findings would generalize to fathers, non-binary caregivers, or other legal guardians. The small number of non-female respondents precluded meaningful analyses of measurement invariance, differential item functioning, or score differences by parent sex/gender. Therefore, we cannot rule out potential rater-related effects associated with parent sex/gender. Future work should recruit more diverse caregiver samples and incorporate multi-informant approaches, including fathers, teachers, clinicians, and child self-report when developmentally appropriate.

In addition, some EFA loadings were modest and near the lower bound of the prespecified exploratory retention criterion. This may reflect the heterogeneity of demand-avoidance-related behaviors and the breadth of content intentionally sampled during item development. Although the final factors were supported by subsequent analyses, future studies are needed to evaluate the DAAQ factor structure in independent samples.

Finally, participants were recruited from an At Peace Parents email listserv, and parents’ prior beliefs about PDA may have shaped their responding; future work should use non-convenience recruitment and incorporate independent characterization of participants. Although the sample was international, it was largely drawn from English-speaking, Global North contexts; cross-cultural adaptation and validation are needed before use in non-English-speaking and Global Majority settings, where cultural norms may shape both expression and interpretation of demand avoidance. Longitudinal research is needed to examine whether and how demand-avoidance behaviors change as children approach adulthood. Although some prior work has reported decreases for some individuals, Newson’s follow-up sample showed heterogeneous trajectories, with avoidance decreasing in some participants, remaining stable in others, and increasing in a smaller number [3,33]. The present cross-sectional study cannot establish that the measured behaviors are developmentally stable or intrinsically pervasive.

## Conclusion

In summary, the DAAQ is the first measure of PDA using rigorous, contemporary measurement development standards and uniquely assesses how demand avoidance affects fulfillment of basic needs (e.g., hygiene, eating, sleeping and toileting), going beyond broad behavioral descriptors. DAAQ scores show strong preliminary psychometric properties. We recommend using the DAAQ as a research screening/phenotyping tool rather than a diagnostic instrument. It may help clinicians and researchers in better characterizing this profile, and, over time, inform evaluation of its potential relevance to DSM consideration. More immediately, it can support understanding of children with a PDA profile and evaluation of treatment outcomes.

**Key Points and Relevance**

- This study closes a key gap in the scientific literature by expanding on the existing pool of knowledge about PDA and how to measure PDA-related characteristics.
- The newly developed DAAQ is a comprehensive, psychometrically sound assessment tool that not only captures PDA-related traits and behaviors, but also encapsulates the impacts on basic needs (hygiene, eating, sleeping and toileting).
- The DAAQ is the first measure of PDA that follows existing measurement development standards and has clinical applicability.
- DAAQ captures a broader scope of PDA-related characteristics than currently available tools and demonstrates strong preliminary psychometric properties.
- This new measure will aid mental health practitioners in better understanding children with a PDA profile to help identify and evaluate effective treatment approaches.

## Supporting information

S1 Table Initial Exploratory Factor Analysis Results of the DAAQ Item Pool (Rotated Factor Pattern). The initial exploratory factor analysis identified five factors. Primary factor loadings and cross-loadings are shown for each item.
(DOCX)

S2 Table Unidimensional Modeling and Analyses for the DAAQ. The table summarizes item retention and removal following exploratory factor analysis and differential item functioning (DIF) analyses by child age, schooling type, parent age, and parent education.
(DOCX)

S3 Table DAAQ Item Parameters. Item discrimination (slope) and category threshold estimates are presented for the 20-item Behavioral Expression, 10-item Basic Needs, and 6-item Toileting scales.
(DOCX)

S1 File Demand Avoidance and Autonomy Questionnaire (DAAQ): Measure and Scoring Manual. The appendix contains the complete 36-item DAAQ, response options, administration and scoring instructions, and raw-score-to-T-score conversion tables for the Behavioral Expression, Basic Needs, and Toileting scales. ©2025 Noelle E. Carlozzi, Ph.D. and Casey Ehrlich, Ph.D. The Demand Avoidance and Autonomy Questionnaire (DAAQ), including its items, instructions, and scoring materials, is licensed under the Creative Commons Attribution 4.0 International License (CC BY 4.0).
(DOC)

S2 File STROBE Statement—checklist of items that should be included in reports of observational studies. (DOCX)

S1 Fig Item history. (PDF)

S2 Fig Conceptual Model. (PDF)

## Abbreviations

ADHD: Attention Deficit Hyperactivity Disorder
ASD: Autism Spectrum Disorder
CAST: Childhood Autism Spectrum Test
CFA: Confirmatory Factor Analysis
CTT: Classical Test theory
DAAQ: Demand Avoidance and Autonomy Questionnaire
EDA-Q: Extreme Demand Avoidance Questionnaire
EDA-8: Extreme Demand Avoidance Questionnaire – 8-item version
EFA: Exploratory Factor Analysis
IRT: Item Response Theory
PDA: Pathological Demand Avoidance
SDQ: Strengths and Difficulties Questionnaire
