# Supplementary material for: The Demand Avoidance and Autonomy Questionnaire (DAAQ): Development of a new measure of demand avoidance
Source: PLoS One. 2026 Nov 11;21(11):e0345827. doi: 10.1371/journal.pone.0345827 (PMC13754100; doi:10.1371/journal.pone.0345827)
Supplement: Supplementary file 1 [file pone.0345827.s006.pdf]

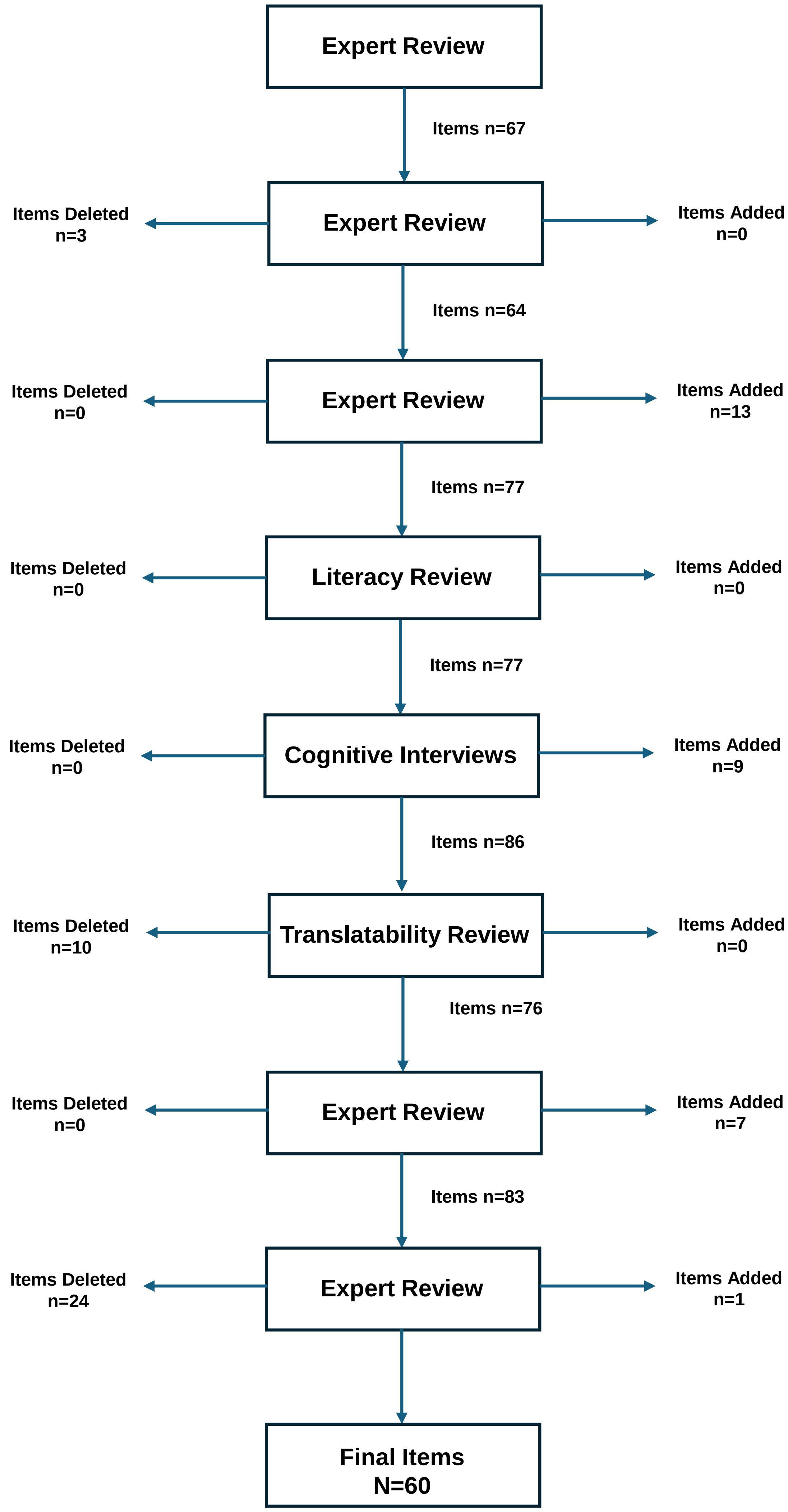
Expert Review
Items n=67
Items Deleted n=3
Expert Review
Items Added n=0
Items n=64
Items Deleted n=0
Expert Review
Items Added n=13
Items n=77
Items Deleted n=0
Literacy Review
Items Added n=0
Items n=77
Items Deleted n=0
Cognitive Interviews
Items Added n=9
Items n=86
Items Deleted n=10
Translatability Review
Items Added n=0
Items n=76
Items Deleted n=0
Expert Review
Items Added n=7
Items n=83
Items Deleted n=24
Expert Review
Items Added n=1
Final Items N=60
