# Supplementary material for: The Demand Avoidance and Autonomy Questionnaire (DAAQ): Development of a new measure of demand avoidance
Source: PLoS One. 2026 Nov 11;21(11):e0345827. doi: 10.1371/journal.pone.0345827 (PMC13754100; doi:10.1371/journal.pone.0345827)
Supplement: Supplementary file 2 [file pone.0345827.s007.pdf]

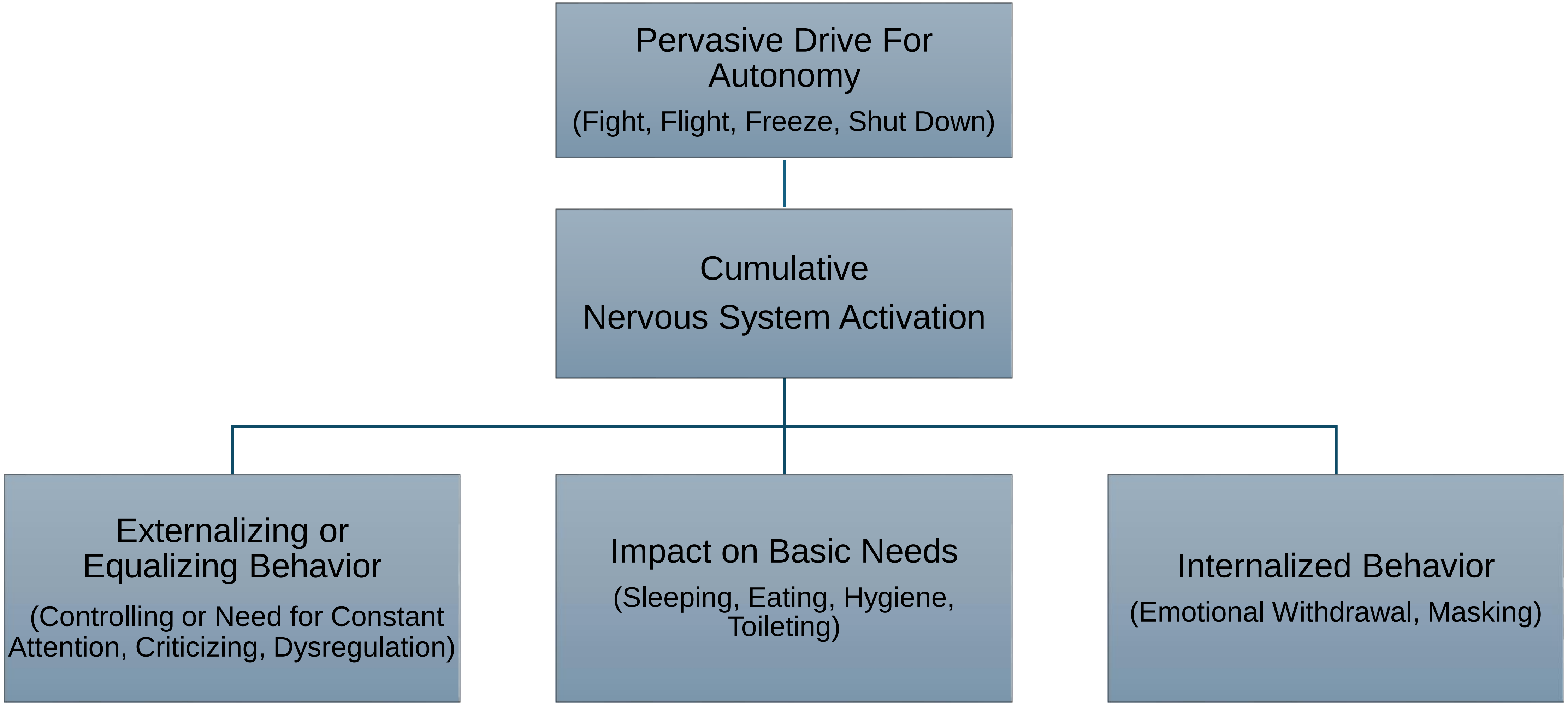
Pervasive Drive For Autonomy
(Fight, Flight, Freeze, Shut Down)
Cumulative
Nervous System Activation
Externalizing or Equalizing Behavior
(Controlling or Need for Constant Attention, Criticizing, Dysregulation)
Impact on Basic Needs
(Sleeping, Eating, Hygiene, Toileting)
Internalized Behavior
(Emotional Withdrawal, Masking)
