# Supplementary material for: The Demand Avoidance and Autonomy Questionnaire (DAAQ): Development of a new measure of demand avoidance
Source: PLoS One. 2026 Nov 11;21(11):e0345827. doi: 10.1371/journal.pone.0345827 (PMC13754100; doi:10.1371/journal.pone.0345827)
Supplement: Supplementary file 3 [file pone.0345827.s009.pdf]

Participant ID_______________ Date _______________

PDA items parent interview/ observational/ multi-informant rating scale

N.B. Ratings: 0= not affected 1=Minor: 2=Marked

Many items and example questions were based on those included in the "Diagnostic Interview for Social and Communication Disorders" (Wing et al,, 2002).

Background information:

Diagnosis:

Date that diagnosis was given:

School history (include number of schools attended, types of schools, exclusion from schools, outside help etc):

(A) Avoids demands using socially manipulative strategies

**1. Obsessively resists and avoids ordinary demands, led by need to control**	0 | 1 | 2
Everything must be on his or her own terms, unable to accept reasonable limits. If pressured to comply, may result in an extreme reaction, or a breakdown in the relationship. Parents shocked to discover how much they have adapted to handling the child with velvet gloves when they start school. S/he may also be indifferent to/ reject praise or react badly to it (e.g. tearing up his/her work).	
• How did things go when A started nursery/ school?<br>• Does A strongly resist attempts to make him or her do things, or change his or her behaviour? What sorts of requests does A avoid?<br>• Does everything have to be on his/ her terms? What happens if you push A to comply or accept limits?<br>• Does A dislike praise?	

**2. Socially manipulative behaviour to avoid demands**	0 | 1 | 2
The child may be generally described as "better than s/he should be" at getting round others, or good at "playing people off against each other". May be skilled at using charm or engaging the adult in such a way that they are genuinely distracted. Social manner may be very polite.	
• Would you describe A as good at getting round others and making them do as s/he wants, or playing people off against each other?<br>• What strategies does A use to get out of things? Are these strategies targeted at a particular person? *(can prompt examples)*<br>☐ Distracting (e.g. asking questions)<br>☐ Apologising and making excuses<br>☐ Withdrawing into role play or toy play<br>☐ Charm<br>☐ Passively (e.g. selective mutism)<br>☐ Other	

Participant ID______________ Date ______________

3. Takes control of the conversation through repetitive questioning/ interrupting	0 | 1 | 2
Tries to get attention and take control of conversation by breaking conversational rules (e.g. asking questions in a manner that feels controlling, with questions fired in rapid succession, possibly accompanied by over-strong eye contact; making statements "Shall I tell you something......" to monopolise the conversation; saying something to provoke the parent). Feels as though the aim is to control, rather than to further a special interest.	
• Does A interrupt conversations to get attention or to take control? If attention is not paid to A, how does s/he react?<br>• How does A do this? (e.g. repeatedly asking questions, make statements to provoke parents attention).	

(B) Surface sociability: apparent lack of social identity, pride, shame or responsibility.

4. Lacks awareness/ is not concerned about age group or social hierarchy	0 | 1 | 2
Child doesn't feel their place in the social hierarchy, and may identify him/herself in an adult role. May be described as "a little policeman" or "a little parent", e.g. stopping peers from going out to play when it is raining outside; taking the psychologist's hand and leading him or her to the assessment room, or taking on role of a teacher during sessions with the psychologist.	
• Does A seem to have a sense of the "pecking order" – that adults can tell children what to do? Or does A treat everyone the same, seeing no difference between himself and adults/ authority figures?<br>• Does A tell other children how they should behave, but does not feel these rules apply to him/herself?<br>• Does A see him/herself as a child?	

5. Difficulties interacting with peers- bossy and domineering	0 | 1 | 2
Dominating and controlling of peers, e.g. insist peers follow their rules in a game. May prefer to play with younger children as it is easier for them to keep control. Parents may need to "manage" friendships with peers.<br>May use peers for some purpose rather than playing with them (e.g. inciting them to throw objects over the wall at play-time).	
• Does A have friends who he/she enjoys spending time with?<br>• How does she interact with her friends/ peers? (e.g. bossy/ controlling, play with them or using them as aids in own activities)<br>• What do peers think of A?	

Participant ID_______________ Date _______________

6. Little shame/ embarrassment and bizarre/ embarrassing remarks in public	0 | 1 | 2
Must be inappropriate for someone of his/her age and developmental level.	
• Does A behave in ways that other people would find embarrassing or shameful in public? (e.g. wetting him/herself, removing clothes in public, screaming through a school play)?<br>• Does A make embarrassing personal remarks, or say bizarre things in public? (e.g. asking strangers inappropriate questions or swearing at them, commenting on others' physical peculiarities)<br>• Is A aware this behaviour is inappropriate or babyish?	

7. Sense of responsibility to others/ social obligation	0 | 1 | 2
Lacks a sense of responsibility to others. Parents know that they cannot appeal to the child's "better nature" to persuade them.	
• R Can you appeal to A's "better nature" to persuade him or her to do things?<br>• R Does A have a sense of obligation towards others (e.g. will stick to a bargain; is nice to friends who come to visit?)<br>• R Can you trust A to tell the truth?	

(C) Surface sociability: difficulties with other people.

8. Difficulties with peers and siblings	0 | 1 | 2
Makes trouble with other children. May severely bully younger siblings, to get a reaction, or be unable to tolerate them getting attention.	
• Does A frequently tease, bully, refuse to take turns, make trouble?	

9. Socially shocking behaviour with deliberate intent	0 | 1 | 2
Uses socially shocking behaviour to control situations, cause distress or get attention (e.g. threatening to dial 999; urinating on the floor; false allegations against other people; racist insults; publicly humiliating others; pinching another child etc.). May also engage in self-injurious behaviour / threaten suicide. **N.B. Behaviour should only be coded here if it appears to have been used purposefully to show they are in control, cause distress or get attention.**	
• Does A shock other people by unexpectedly inappropriate actions, for no apparent reason? What does A do?<br>• Does A ever threaten to hurt him/herself, or do things to hurt him/herself?<br>• Is this behaviour impulsive, or does A do it on purpose to show s/he is in control, cause distress or get attention?	

Participant ID_______________ Date _______________

10. Blames others inappropriately/ makes false accusations	0 | 1 | 2
Blames others inappropriately for own misdeeds, or when things don't turn out the way that they had hoped. Unable to accept responsibility. Blaming this may have an irrational or obsessional quality (e.g. one child blamed her grandmother because the tide came in and washed over her sand castle). May falsely accuse others of hurting them, and persist in making accusations even when the accusation is given no credence.	
• Does A blame other people when things go wrong?<br>• Does this have an irrational and obsessional quality?<br>• Does A accuse people of things they haven't done?	

11. Unkind to younger children and pets	0 | 1 | 2
Score 1 for "cannot be relied upon to be kind" and 2 for "is unkind on purpose for no apparent reason" (would not apply to the examples given above)"	
• R Can you rely on A always to be kind to younger children and pets?<br>• R Would you be happy to leave A alone with a younger child (e.g. let him/her babysit), knowing that s/he would be kind?	

12. Lacks emotional response to others' distress	0 | 1 | 2
The child may not react when others are upset, or may react paradoxically (e.g. displaying amusement or excitement). May feel no remorse for incidents, and may have a poor awareness of others' emotions.	
• Does A react inappropriately to others' emotions (e.g. becoming excited when someone is upset, failing to react at all?).	

(D) Extreme emotional lability

13. Changeable mood/ extreme emotional responses	0 | 1 | 2
Child displays frequent and sudden changes in mood, in response to small events (e.g. suddenly lashing out at others with no warning, or flying into a rage over something very minor). They may appear to be turned on and off so that they seem to lack an "emotional legacy".	
• Does A have frequent marked swings of mood for no obvious reason, or in response to minor things?<br>• Do switches in mood lack an "emotional legacy" – changing suddenly from one mood to the next?	

Participant ID_______________ Date _______________

14. Rapid, inexplicable changes from loving to aggression	0 | 1 | 2
Suddenly slips from loving or affectionate to aggressive behaviour with no warning.	
• Does A slip from loving to violent or aggressive behaviour (or vice versa) for no apparent reason?<br>• Does A seem to display affection in an aggressive way (e.g. squeezing someone too hard; digging their nails in whilst holding hands).	

15. Laughs or becomes "silly" or over-excited for no reason	0 | 1 | 2
Slips from calm to silly or over-excited for no reason. For example, one boy laughs or screams uncontrollably for no reason, and has to hit himself on the face to stop the laughing.	
• Does A ever laugh for no reason? Is this laughter unusual?<br>• Does A ever seem to get "high" or extremely over-excited, and if so, what brings this on?	

(E) Comfortable in fantasy and role play

16. Role play/ imaginative play – lives the part, not the usual pretence (not echolalia)	0 | 1 | 2
Engages in role play to the extent s/he seems to forget who s/he really is, and gets lost in the game. This must be a new or embellished role, rather than just echolalia. Alternatively, the child may engage in imaginative play that is surprising in intensity.	
• Does A act out the role of an object, animal, fictional person or real person, so that A seems to become the acted role- it is not just pretence? If not role play, imaginative play?<br>• Are these new or embellished stories, or just copied exactly from TV or films?<br>• Is this a major part of A's play/ unusual for someone of their age?<br>• Is it difficult for A to come back to reality?	

17. Social mimicry	0 | 1 | 2
Adapts others' voices, styles, body language and persona when interacting with others. For example, copies tone of teacher when in therapy sessions. This should be used creatively, and periods of echolalia don't count.	
• Does A mimic adult mannerisms and styles from people s/he knows, computer games or tv (e.g. uses phrases adopted from teacher/parent to tell other children off)?<br>• Uses over-animated facial expressions, possibly practising these in front of a mirror?	

Participant ID________________ Date ________________

18. Fantasises, tells tall tales	0 | 1 | 2
Tells tall tales in a way that is inappropriate for their age, and at times as if they believe them to be real (e.g. events that happened at the weekend).	
• Tells tall tales in a way that is inappropriate for their age<br>• Does A talk about fantasies as if real?	

19. Communicates indirectly (e.g. through doll, props, picture cards, puppet, toy animal, or as a persona)	0 | 1 | 2
Child seems more comfortable communicating at second hand. Child may also be only able to convey feelings in this manner. This should be beyond what is developmentally appropriate.	
• Does A use a doll, puppet or object to communicate needs?<br>• Does A seem more comfortable communicating indirectly (e.g. using picture cards instead of speaking; having adopted a role)?	

(F) Obsessions centred on people or their characteristics

20. Obsessed with a particular person, real or fictional	0 | 1 | 2
May be obsessed with a peer or well known person. May be very possessive and smother the peer. May take the form of stalking or harassing another person (e.g. writing threatening letters; untrue accusations)?	
• Is A obsessed with a person, real or fictional; or have over-intense friendships with others?<br>• Does A behave in inappropriate ways towards this person, perhaps causing them distress? (A may display love or hatred towards this person).	

Participant ID________________ Date ________________

(G) Early history/ language delay

21. Unusually quiet & passive in infancy; hands limp for unwelcome tasks	0 | 1 | 2
Describe the child as easy-going, or a very good baby. May be "too good", e.g. "You could have left him on a sofa indefinitely". Can be clingy, but may not seek out affection, though may accept it passively.<br>The child may drop things placed in his/her hands and doesn't bother picking them up, reluctant to crawl or walk. The child may even seem to be floppy. Becomes more "actively resistant" over time (e.g. hands seem limp and weak for unwelcome tasks; refuses to look at toys, or to join in with play).<br>**NB: If baby was the first, parents may not have recognised this behaviour as unusual early on.**	
∘ Was A an exceptionally good baby compared to others of the same age? Did you feel "I did not know I had a baby in the house"?<br>∘ Was A very passive (e.g. reluctant to crawl; dropped objects placed in his/her hands, and did not reach for them)? Did it seem as though nothing was worth the bother for A?<br>∘ Was A 'floppy' or limp as a baby?<br>∘ Was A content to watch other children play - observing rather than joining in?<br>∘ Did A become more actively resistant over time?	

22. Language delay	0 | 1 | 2
Parent was concerned about child's delay in talking. Often, once the child starts to speak, the catch up is rapid, as if not talking was due to passivity rather than a language delay.	
∘ Were you concerned about your child starting to talk later than others born around the same time?<br>∘ Once s/he had started talking, did s/he seem to catch up very rapidly?	
