# Supplementary material for: The Demand Avoidance and Autonomy Questionnaire (DAAQ): Development of a new measure of demand avoidance
Source: PLoS One. 2026 Nov 11;21(11):e0345827. doi: 10.1371/journal.pone.0345827 (PMC13754100; doi:10.1371/journal.pone.0345827)
Supplement: Supplementary file 4 [file pone.0345827.s001.docx]

| Supplemental Table 1  Initial Exploratory Factor Analysis Results of the DAAQ Item Pool (Rotated Factor Pattern) | | | | | | | | | |  |  |  |
| --- | --- | --- | --- | --- | --- | --- | --- | --- | --- | --- | --- | --- |
| Item | Factor 1 | Factor 2 | | | Factor 3 | | Factor 4 | | Factor 5 | | | |
| When playing games, my child becomes distressed (dysregulated/agitated) if they are not winning. | **0.52** | -0.01 | | | -0.03 | | 0.08 | | 0.03 | | | |
| When playing games, my child attempts to change the rules of the game, cheat, or control other people’s behavior in order to win. | **0.65** | -0.10 | | | -0.06 | | 0.10 | | -0.01 | | | |
| My child has difficulty with transitions to a preferred activity. | **0.33** | 0.22 | | | 0.01 | | 0.01 | | 0.06 | | | |
| If I try to encourage my child to participate in activities they dislike, my child will become distressed (dysregulated/agitated) and/or have a meltdown (panic attack/temper tantrum). | **0.42** | 0.27 | | | -0.08 | | 0.02 | | 0.06 | | | |
| If I try to incentivize (sticker charts, rewards) my child to participate in activities they dislike, my child will become distressed (dysregulated/agitated) and/or have a meltdown (panic attack/temper tantrum). | **0.30** | **0.31** | | | -0.02 | | 0.03 | | 0.11 | | | |
| When my child is in a situation they find stressful, my child shows flight behavior (running or climbing on something to get away, crawling underneath something, hiding). | **0.33** | 0.00 | | | 0.07 | | 0.08 | | 0.05 | | | |
| When my child is in a situation they find stressful, my child behaves inappropriately (laughs or says something inappropriate, removes their clothes, makes inappropriate sounds or noises, uses nonsense words, repeats words). | **0.54** | -0.13 | | | 0.04 | | 0.03 | | 0.01 | | | |
| When my child is in a situation they find stressful, my child shows fight behavior (kicking, hitting, biting, screaming, swearing, hissing, growling, throwing things, knocking things over, destroying things). | **0.59** | -0.18 | | | 0.03 | | 0.06 | | -0.02 | | | |
| When my child is in a situation they find stressful, my child shows freeze behavior (withdrawing, hiding, shutting down, disengaging, entire body going limp, not speaking/nonresponsive, blank stare or glazed eyes, not moving/saying they can't move or their | -0.16 | **0.35** | | | 0.13 | | -0.08 | | -0.02 | | | |
| My child becomes distressed (dysregulated/agitated) when they are told that they have to stop doing a preferred activity (e.g., screen time). | **0.44** | 0.28 | | | -0.06 | | -0.07 | | 0.06 | | | |
| My child will refuse help, even after they have asked for it. | **0.39** | 0.26 | | | -0.05 | | -0.10 | | -0.01 | | | |
| When my child is told to do something, they will comply but do things in a different or more difficult way. | **0.36** | 0.02 | | | 0.04 | | -0.07 | | -0.01 | | | |
| When my child is told to do something, they will say, I can't, I don't feel well, ignore the request, act as if they do not understand (change the topic, pretend not to hear, start doing something unrelated), pretend to be an animal, or leave the room. | **0.38** | 0.20 | | | 0.02 | | -0.14 | | 0.05 | | | |
| My child destroys their own things. | **0.57** | -0.04 | | | 0.08 | | -0.02 | | -0.07 | | | |
| My child uses body-focused repetitive behavior (picking skin, nail biting, pulling out hair, pulling out eyebrow hair, scratching, cutting). | 0.17 | **0.31** | | | -0.04 | | -0.07 | | 0.00 | | | |
| When my child is excited about something (e.g., a holiday), they become distressed (dysregulated/agitated). | **0.37** | 0.18 | | | 0.04 | | 0.08 | | 0.01 | | | |
| My child will show excitement about something only to refuse when it is time to participate (leave the house, go into building/event). | **0.42** | 0.23 | | | -0.05 | | 0.03 | | 0.00 | | | |
| My child will respond to being told No, Stop or Don't by doing the opposite, even if it is dangerous (runs towards the street, touches a hot stove, climbs a high ledge). | **0.65** | -0.13 | | | 0.11 | | 0.01 | | -0.02 | | | |
| When my child does not get their way, they will respond by knocking something down, shouting, calling names, needing to have the last word, or destroying something. | **0.69** | -0.04 | | | -0.10 | | -0.01 | | -0.06 | | | |
| My child uses self-harm (cutting, picking, self-hitting, hair pulling, skin picking, running into things, doing things that are dangerous) when they are having a meltdown (panic attack/temper tantrum) or are distressed (dysregulated/agitated). | 0.17 | 0.29 | | | -0.03 | | 0.05 | | -0.03 | | | |
| My child uses negative self-talk (I'm no good, I'm bad, I'm stupid, I want to die, I'm going to kill myself) when they are having a meltdown (panic attack/temper tantrum) or are distressed (dysregulated/agitated). | 0.28 | 0.21 | | | -0.18 | | -0.08 | | -0.01 | | | |
| My child likes to be physically above people (e.g., likes to sit or stand above other people, stand on chairs or the couch, climb onto windowsills, sit on the back of the couch). | **0.42** | 0.03 | | | 0.07 | | 0.12 | | 0.08 | | | |
| My child breaks/ignores rules but gets upset if others do not follow them. | **0.59** | 0.06 | | | -0.01 | | -0.11 | | -0.07 | | | |
| When I ask my child a direct question, they respond with a growl or hissing sound, an I don't know, stop talking, silence, or a silly word like poop. | **0.55** | 0.11 | | | 0.07 | | -0.18 | | 0.00 | | | |
| When I tell my child to do something, they respond with a growl or hissing sound, an I don't know, stop talking, silence, or a silly word like poop. | **0.56** | 0.15 | | | 0.03 | | -0.13 | | -0.02 | | | |
| My child needs constant attention, or they will become distressed (dysregulated/agitated) or have a meltdown (panic attack/temper tantrum). | **0.41** | -0.03 | | | 0.07 | | **0.35** | | -0.06 | | | |
| Unless my child is participating in a preferred activity (e.g., on a screen or a special interest), they become distressed (dysregulated/agitated) or have a meltdown (panic attack/temper tantrum) when I try to talk to another person. | **0.53** | 0.06 | | | 0.06 | | 0.22 | | 0.03 | | | |
| Unless my child is participating in a preferred activity (e.g., on a screen, or a special interest), or with someone they trust, he/she will become distressed (dysregulated/agitated) or have a meltdown (panic attack/temper tantrum) if I leave. | 0.25 | 0.17 | | | 0.07 | | **0.40** | | 0.00 | | | |
| My child wants me to stay nearby but does not want me to touch or interact with them. | **0.37** | 0.24 | | | 0.03 | | 0.05 | | 0.02 | | | |
| My child will only participate in fun activities (e.g., play/sports, drawing, or building) if someone they trust is also participating in the activity. | 0.13 | **0.30** | | | 0.05 | | 0.21 | | -0.01 | | | |
| My child focuses on one or two preferred topics/activities. | 0.10 | **0.33** | | | -0.06 | | -0.02 | | 0.11 | | | |
| My child appears like two different versions of themselves, one inside the home, and one outside the home. | 0.25 | 0.20 | | | 0.05 | | -0.06 | | -0.04 | | | |
| My child appears fine (behaves appropriately) in settings outside of the home (e.g., school/daycare, a grandparent's house) but is prone to meltdowns (panic attack/temper tantrum or misbehavior) when they return home. | **0.30** | 0.12 | | | 0.00 | | -0.03 | | -0.06 | | | |
| My child appears fine (behaves appropriately) at home but is prone to meltdowns (panic attack/temper tantrum or misbehavior) in settings outside of the home (e.g., school/daycare, a grandparent's house). | 0.21 | -0.07 | | | 0.01 | | 0.11 | | 0.01 | | | |
| My child is fine (behaves appropriately) in settings outside of the home (e.g., school/daycare, a grandparent's house) but wants to be alone when they return home. | -0.04 | **0.36** | | | -0.09 | | -0.24 | | 0.09 | | | |
| I can predict when my child will get angry. | 0.01 | -0.01 | | | -0.06 | | -0.02 | | **0.89** | | | |
| I can predict when my child will have a meltdown (panic attack/temper tantrum). | -0.03 | -0.02 | | | 0.09 | | -0.03 | | **0.93** | | | |
| I can predict when my child will have a good day vs. a bad day. | -0.01 | 0.03 | | | -0.02 | | 0.05 | | **0.57** | | | |
| My child will do something (e.g., putting on shoes, going to a store) without difficulty some days but not others. | 0.03 | 0.15 | | | 0.07 | | -0.05 | | 0.05 | | | |
| My child resists eating. | 0.04 | **0.30** | | | 0.11 | | 0.02 | | 0.04 | | | |
| My child needs to be in control of when, where and/or what they eat. | 0.02 | **0.60** | | | 0.06 | | 0.01 | | 0.09 | | | |
| My child will only eat preferred foods. | -0.03 | **0.48** | | | 0.02 | | 0.07 | | 0.07 | | | |
| My child refuses to eat food at a table. | 0.12 | **0.35** | | | 0.07 | | 0.11 | | 0.05 | | | |
| My child resists sleeping. | 0.00 | **0.46** | | | 0.06 | | 0.22 | | -0.11 | | | |
| My child needs to be in control of when and/or where they sleep. | 0.00 | **0.64** | | | -0.08 | | 0.15 | | -0.01 | | | |
| My child is only able to fall asleep if someone else is in the room with them. | -0.09 | -0.01 | | | -0.01 | | **0.95** | | 0.03 | | | |
| My child is only able to sleep through the night if someone else is in the same room. | -0.05 | 0.08 | | | -0.04 | | **0.90** | | 0.00 | | | |
| My child has an irregular sleep cycle (e.g., they go to sleep at different times and wake up at different times each night/day). | 0.00 | **0.48** | | | -0.03 | | 0.12 | | -0.05 | | | |
| My child resists going to the bathroom. | -0.06 | 0.09 | | | **0.90** | | -0.05 | | -0.04 | | | |
| My child needs to be in control of when and/or where they go to the bathroom (or have diaper changes). | -0.09 | 0.27 | | | **0.70** | | -0.01 | | 0.01 | | | |
| My child has lost skills related to toileting (needing to use a diaper again, toileting outside or in a particular location like the bathtub or refusing all together). | 0.05 | -0.01 | | | **0.75** | | 0.05 | | 0.02 | | | |
| My child has toileting accidents. | 0.09 | -0.17 | | | **0.73** | | 0.03 | | 0.03 | | | |
| When my child becomes distressed (dysregulated/agitated), their ability to toilet independently decreases (wipe themselves, use the toilet alone). | 0.11 | -0.07 | | | **0.69** | | 0.16 | | 0.03 | | | |
| My child withholds his/her urine (pee) or stool (poop). | 0.02 | 0.06 | | | **0.84** | | -0.14 | | -0.02 | | | |
| My child resists bathing or showering. | -0.01 | **0.60** | | | 0.09 | | -0.08 | | -0.09 | | | |
| My child needs to be in control of when and/or where and/or how they bathe or shower. | -0.06 | **0.80** | | | -0.01 | | 0.00 | | 0.02 | | | |
| My child resists brushing their teeth. | 0.09 | **0.58** | | | -0.04 | | -0.05 | | -0.04 | | | |
| My child needs to be in control of when and/or where and/or how they brush their teeth. | -0.01 | **0.79** | | | -0.05 | | 0.00 | | -0.02 | | | |
| My child resists brushing their hair. | 0.01 | **0.54** | | | 0.09 | | 0.00 | | -0.06 | | | |
| My child needs to be in control of when and/or where and/or how they brush their hair. | -0.05 | **0.76** | | | 0.01 | | 0.01 | | -0.04 | | | |
| % of variance explained | 11.25 | 2.30 | | | 3.78 | | 1.87 | | 2.75 | | | |
|  | | |  |  | |  | |  | | |  |  |
