# Supplementary material for: The Demand Avoidance and Autonomy Questionnaire (DAAQ): Development of a new measure of demand avoidance
Source: PLoS One. 2026 Nov 11;21(11):e0345827. doi: 10.1371/journal.pone.0345827 (PMC13754100; doi:10.1371/journal.pone.0345827)
Supplement: Supplementary file 5 [file pone.0345827.s002.docx]

Supplemental Table 2

Unidimensional Modeling and Analyses for the DAAQ

|  |  | CFA Modeling | | | IRT Analysis | | Final items |
| --- | --- | --- | --- | --- | --- | --- | --- |
| Factor | Item pool | R-squared or Bi-Factor Loading  (criterion >0.3) | Residual correlation, only  (criterion <.15) | Concept coverage | IRT Misfit | DIF Analysis |  |
| Factor 1: Behavioral Expression | 25 items | 2  items* | 4  items* | 0  items | 0 items | 0  items | 20  items |
| Factor 2:  Basic Needs | 17 items | 1  item | 1  item | 5  items | 0 items | 0  items | 10  items |
| Factor 3: Toileting | 6 items | 0 items | 0  items | 0  items | 0 items | 0  items | 6  items |

*Note.* CFA = Confirmatory Factor Analysis. * indicates that one item was flagged for exclusion for two separate criterion (low factor loading and a high residual correlation)
