# Supplementary material for: The Demand Avoidance and Autonomy Questionnaire (DAAQ): Development of a new measure of demand avoidance
Source: PLoS One. 2026 Nov 11;21(11):e0345827. doi: 10.1371/journal.pone.0345827 (PMC13754100; doi:10.1371/journal.pone.0345827)
Supplement: Supplementary file 6 [file pone.0345827.s003.docx]

| Supplemental Table 3  DAAQ Item Parameters | | | | | | |
| --- | --- | --- | --- | --- | --- | --- |
| Factor 1: Behavioral Expression | Slope | Threshold 1 | Threshold 2 | Threshold 3 | Threshold 4 | Threshold 5 |
| When playing games, my child becomes distressed (dysregulated/agitated) if they are not winning. | 1.02 | -5.17 | -3.29 | -1.26 | 0.91 |  |
| My child has difficulty with transitions to a preferred activity. | 0.93 | -4.88 | -2.90 | -0.55 | 1.45 |  |
| If I try to encourage my child to participate in activities they dislike, my child will become distressed (dysregulated/agitated) and/or have a meltdown (panic attack/temper tantrum). | 1.22 | -5.42 | -4.35 | -1.95 | 0.28 |  |
| When my child is in a situation they find stressful, my child behaves inappropriately (laughs or says something inappropriate, removes their clothes, makes inappropriate sounds or noises, uses nonsense words, repeats words). | 0.93 | -2.90 | -1.39 | 0.46 | 2.30 |  |
| My child becomes distressed (dysregulated/agitated) when they are told that they have to stop doing a preferred activity (e.g., screen time). | 1.17 | -4.37 | -1.50 | 0.68 |  |  |
| My child will refuse help, even after they have asked for it. | 0.99 | -4.49 | -2.24 | 0.57 | 3.17 |  |
| When my child is told to do something, they will say, “I can’t”, ”I don’t feel well”, ignore the request, act as if they do not understand (change the topic, pretend not to hear, start doing something unrelated), pretend to be an animal, or leave the room. | 0.88 | -6.04 | -4.10 | -1.17 | 1.87 |  |
| My child destroys their own things. | 1.14 | -2.59 | -0.95 | 1.51 | 3.24 |  |
| When my child is excited about something (e.g., a holiday), they become distressed (dysregulated/agitated). | 1.11 | -3.62 | -2.03 | 0.13 | 1.63 |  |
| My child will show excitement about something only to refuse when it is time to participate (leave the house, go into building/event). | 1.28 | -3.14 | -1.70 | 0.31 | 2.36 |  |
| My child will respond to being told “No,” “Stop” or “Don’t” by doing the opposite, even if it is dangerous (runs towards the street, touches a hot stove, climbs a high ledge). | 1.35 | -2.62 | -1.25 | 0.56 | 2.24 |  |
| When my child does not get their way, they will respond by knocking something down, shouting, calling names, needing to have the last word, or destroying something. | 1.24 | -3.74 | -2.62 | -0.89 | 0.97 |  |
| My child likes to be physically “above” people (e.g., likes to sit or stand above other people, stand on chairs or the couch, climb onto windowsills, sit on the back of the couch). | 1.02 | -3.37 | -1.31 | -0.60 | 0.83 | 2.32 |
| My child breaks/ignores rules but gets upset if others do not follow them. | 1.11 | -4.25 | -2.90 | -2.21 | -0.87 | 0.54 |
| When I ask my child a direct question, they respond with a growl or hissing sound, an “I don’t know”, “stop talking”, silence, or a silly word like “poop”. | 1.27 | -3.42 | -2.32 | 0.07 | 2.04 |  |
| When I tell my child to do something, they respond with a growl or hissing sound, an “I don’t know”, “stop talking”, silence, or a silly word like “poop”. | 1.37 | -3.24 | -1.97 | -0.05 | 1.91 |  |
| My child needs constant attention, or they will become distressed (dysregulated/agitated) or have a meltdown (panic attack/temper tantrum). | 1.26 | -3.09 | -2.08 | -1.53 | -0.28 | 1.02 |
| Unless my child is participating in a preferred activity (e.g., on a screen or a special interest), they become distressed (dysregulated/agitated) or have a meltdown (panic attack/temper tantrum) when I try to talk to another person. | 1.86 | -2.39 | -1.44 | -0.25 | 1.09 |  |
| Unless my child is participating in a preferred activity (e.g., on a screen, or a special interest), or with someone they trust, he/she will become distressed (dysregulated/agitated) or have a meltdown (panic attack/temper tantrum) if I leave. | 1.25 | -2.55 | -1.14 | 0.15 | 1.66 |  |
| My child wants me to stay nearby but does not want me to touch or interact with them. | 1.18 | -2.66 | -1.35 | 0.63 | 2.50 |  |
| Factor 2: Basic Needs |  |  |  |  |  |  |
| My child needs to be in control of when, where and/or what they eat. | 1.33 | -3.88 | -3.04 | -2.48 | -1.13 | 0.13 |
| My child will only eat preferred foods. | 0.92 | -5.04 | -3.32 | -2.63 | -1.27 | 0.09 |
| My child resists sleeping. | 1.08 | -3.12 | -1.53 | 0.26 | 2.12 |  |
| My child needs to be in control of when and/or where they sleep. | 1.50 | -2.99 | -2.03 | -1.45 | -0.38 | 0.76 |
| My child has an irregular sleep cycle (e.g., they go to sleep at different times and wake up at different times each night/day). | 0.96 | -1.65 | 0.01 | 1.22 | 2.46 |  |
| My child resists bathing or showering. | 1.30 | -2.78 | -1.60 | -0.33 | 1.23 |  |
| My child needs to be in control of when and/or where and/or how they bathe or shower. | 2.61 | -2.74 | -2.11 | -1.72 | -0.77 | 0.12 |
| My child resists brushing their teeth. | 1.42 | -3.21 | -1.83 | -0.52 | 0.77 |  |
| My child needs to be in control of when and/or where and/or how they brush their teeth. | 2.55 | -2.82 | -2.00 | -1.70 | -0.84 | 0.09 |
| My child needs to be in control of when and/or where and/or how they brush their hair. | 1.91 | -2.26 | -1.72 | -1.48 | -0.68 | 0.15 |
| Factor 3: Toileting |  |  |  |  |  |  |
| My child resists going to the bathroom. | 3.29 | -1.20 | -0.47 | -0.20 | 0.51 | 1.16 |
| My child needs to be in control of when and/or where they go to the bathroom (or have diaper changes). | 1.90 | -1.86 | -1.04 | -0.77 | -0.10 | 0.94 |
| My child has lost skills related to toileting (needing to use a diaper again, toileting outside or in a particular location like the bathtub, or refusing all together). | 2.25 | -0.40 | 0.36 | 0.62 | 1.19 | 1.78 |
| My child has toileting accidents. | 1.82 | -0.43 | 0.53 | 1.68 | 2.52 |  |
| When my child becomes distressed (dysregulated/agitated), their ability to toilet independently decreases (wipe themselves, use the toilet alone). | 2.03 | -0.54 | -0.09 | 0.53 | 1.30 |  |
| My child withholds his/her urine (pee) or stool (poop). | 2.48 | -0.72 | -0.16 | 0.80 | 1.77 |  |
