# Supplementary material for: The Demand Avoidance and Autonomy Questionnaire (DAAQ): Development of a new measure of demand avoidance
Source: PLoS One. 2026 Nov 11;21(11):e0345827. doi: 10.1371/journal.pone.0345827 (PMC13754100; doi:10.1371/journal.pone.0345827)
Supplement: Supplementary file 7 [file pone.0345827.s005.docx]

STROBE Statement—checklist of items that should be included in reports of observational studies

|  | Item No. | Recommendation | Page  No. | Relevant text from manuscript |
| --- | --- | --- | --- | --- |
| **Title and abstract** | 1 | (*a*) Indicate the study’s design with a commonly used term in the title or the abstract | 1 &3 | The Pathological Demand Avoidance Questionnaire (PDAQ):  Development of a new measure of demand avoidance  Our team expanded upon this previous work by developing a new measure according to established measurement development standards – the Pathological Demand Avoidance Questionnaire (PDAQ) – to capture PDA-specific traits, behaviors, and the impact of these traits in these families’ daily lives. |
|  |  | (*b*) Provide in the abstract an informative and balanced summary of what was done and what was found | 3 | Our team expanded upon this previous work by developing a new measure according to established measurement development standards – the Pathological Demand Avoidance Questionnaire (PDAQ) – to capture PDA-specific traits, behaviors, and the impact of these traits in these families’ daily lives. Factor analysis supported retention of a total of 36 items across three factors; Behavioral Expression (20 items), Basic Needs (10 items), and Toileting (6 items); these 36 items comprise the PDAQ. Differential item functioning for child age, child education, child school type, child sex, parent age, and parent education did not result in any items being deleted. Preliminary data for the PDAQ supported internal consistency, reliability’ and convergent, discriminant, and known-groups validity. |
| Introduction | | | |  |
| Background/rationale | 2 | Explain the scientific background and rationale for the investigation being reported | 5-7 | Pathological demand avoidance (PDA; also known as “persistent” or “extreme” demand avoidance and persistent drive for autonomy [1]) is a complex construct that is used to describe a range of unique behaviors observed in some children with autism spectrum disorder (ASD). PDA is characterized by an extreme need to maintain control in response to everyday demands (external as well as internal) and situations both within and outside the home. When they cannot avoid or cope with a demand or situation, they may involuntarily experience intense physical or emotional reactions (“meltdowns”); “freeze” or shutdown episodes; flight, fight, or fawn responses; anger and aggressive outbursts; and/or panic, among other things.[2-5] Anecdotally, we postulate that this heightened nervous system activation (i.e., the frequent experience of fight or flight) may also impact the ability for these children to access basic needs. Regardless, the PDA profile is associated with profound challenges for both the children living with these experiences and their families.[4]  While recognition and discussion of PDA has increased among some practitioners and clinicians in recent years, in newly published literature, and through online testimonies of adults with lived experiences of PDA[6-8], widespread debate exists over the validity of PDA as a diagnosis. While PDA has commonly been described as a profile or subtype of autism, some conceptualize PDA as a distinct developmental disorder that is separate from autism or other neurotypes such as attention deficit hyperactivity disorder (ADHD) [9]. Others have suggested that PDA may be better defined as a set of symptoms versus a unique condition [10]. Further, autism researchers and autistic self-advocates have expressed concerns that the concept of PDA undermines the agency and self-advocacy of individuals with autism and posit that the behaviors observed are better described as driven by “rational” demand avoidance [11].  The lack of sufficient scientific evidence to support inclusion of PDA in either the American *Diagnostic and Statistical Manual of Mental Disorders* (DSM; American Psychiatric Association) or the European *International Classification of Diseases* (ICD; World Health Organization) diagnostic reference manuals [12] has stymied our progress in addressing this ongoing controversy and left families of children with PDA traits feeling unseen and unable to access the interventions and support they need.[13] The absence of specific clinical guidance is especially problematic when providers default to using treatment strategies designed for general autism spectrum conditions with children who have PDA, as these may not only be counterproductive but also may inadvertently increase the fight or flight response commonly observed in these children.  A major factor that has significantly impeded research in this area is that no consensus approach for measuring PDA currently exists. To date, the only measure specifically designed to capture PDA traits is the Extreme Demand Avoidance Questionnaire (EDA-Q [14-16]) The development work supporting the original 26-item, parent-report questionnaire (for parents of children ages 5-17) indicates preliminary support for internal consistency, content validity, sensitivity and specificity [15]. However, the psychometric properties of this measure have not yet been established outside of this work. Finally, no previous PDA research (including the EDA-Q) has focused on or measured a potentially significant aspect of PDA that has been widely reported anecdotally – the inability of many children with PDA to access basic needs such as eating, sleeping, toileting, and hygiene. This is a key gap in the literature that this study will address.  More recently, the developers of the EDA-Q used item response theory and differential item functioning analyses to refine the original measure; the development work for this new 8-item version of the EDA-Q (EDA-8) indicates good internal consistency, and convergent and discriminant validity. This work has yet to be replicated outside of the development team, and known groups validity, predictive validity, sensitivity and specificity have not yet been established [16]. While results for this measure are promising, more work is needed to establish the psychometric properties of both measures (independent of the initial development work); especially given that the sample size for the refinement work (N=334) is lower than what is recommended to achieve stable item parameter information in item response theory models [17]. More importantly, there are several conceptual limitations for this measure, including that the item content does not account for demand avoidance that prevents access to basic needs or causes internalized expressions of PDA. Finally, although the brevity of the EDA-8 is a strength, the items are limited in terms of content coverage of PDA-specific behavioral traits. These factors, as well as the controversy supporting PDA, have limited the uptake of the EDA-Q/EDA-8 in the assessment literature. |
| Objectives | 3 | State specific objectives, including any prespecified hypotheses | 7 | The purpose of this study was to develop a new, psychometrically sound assessment – the Pathological Demand Avoidance Questionnaire (PDAQ) – that can more comprehensively capture PDA-specific traits, internalized and externalized behaviors, and impact that demand avoidance can have on basic needs such as eating, sleeping, toileting, and hygiene. To do this, we followed a rigorous process in accordance with established measurement development standards [18]. Specifically, we employed both classical test theory and item response theory for measurement development, and item content was derived based on information from key stakeholders (parents of PDA children or teens, PDA adults, treatment providers, and measurement development experts), as well as responses to a preliminary questionnaire that was completed by a purposeful sample of 15 parents of PDA children or teens between the ages of 3 and 17 on January 6^th^, 2022. |
| Methods | | | |  |
| Study design | 4 | Present key elements of study design early in the paper | 9 | Participants completed surveys online via a study-specific Research Electronic Data Capture (REDCap) [23] web link. |
| Setting | 5 | Describe the setting, locations, and relevant dates, including periods of recruitment, exposure, follow-up, and data collection | 8 | Participants were recruited using an email list of subscribers targeting a PDA audience. An announcement for the study, along with a link to the survey, was delivered to email subscribers of At Peace Parents on November 6^th^, 2023. |
| Participants | 6 | (*a*) *Cohort study*—Give the eligibility criteria, and the sources and methods of selection of participants. Describe methods of follow-up  *Case-control study*—Give the eligibility criteria, and the sources and methods of case ascertainment and control selection. Give the rationale for the choice of cases and controls  *Cross-sectional study*—Give the eligibility criteria, and the sources and methods of selection of participants | 8 | This sample included 707 self-identified parents or legal guardians of children aged 4-17 with a suspected PDA profile. Respondents had to be at least 18 years old and be able to read/comprehend English. |
|  |  | (*b*) *Cohort study*—For matched studies, give matching criteria and number of exposed and unexposed  *Case-control study*—For matched studies, give matching criteria and the number of controls per case |  | N/A |
| Variables | 7 | Clearly define all outcomes, exposures, predictors, potential confounders, and effect modifiers. Give diagnostic criteria, if applicable | 9-11 | Analysis Plan  *Survey Development*. Analyses for the development of the PDAQ were conducted according to established standards and involved both classical test theory (CTT) and item response theory (IRT) [18]. We first evaluated the dimensionality of the item pool. The sample was randomly divided into two datasets: one for exploratory factor analysis (EFA) and the other for confirmatory factor analysis (CFA). EFA was used to explore potential factor structure among items. We explored the number of factors based on number of factors with eigenvalue > 1 and the scree plot (i.e., number of factors before the break in scree plot). EFA with a promax rotation with polychoric correlations was then used to examine the association among factors and items and to examine item loadings (criterion: >0.3). CFA was conducted using the weighted least square-mean and variance adjusted estimator with polychoric correlations [24]. Item sets were considered unidimensional, with their items retained in the item pool, when the following criteria were met: comparative fit index (CFI) >0.9; root mean square error of approximation (RMSEA <0.1 [25]; R2 >0.3; correlated error modification index <10; residual correlations <0.15 [26]). Samejima’s graded response model (GRM) was used to identify items with significant misfit (S-X2 / df effect size >3) [27] using IRTPRO (version 3.1.2) [28]. Differential item functioning (DIF) was investigated to identify potential item bias (e.g., to identify items that might unfairly discriminate for or against individuals in a given group) [29] using R package LORDIF Version 0.3-2 [30]. DIF was evaluated by child age, child education, child school type, child sex, parent age, and parent education. Items with DIF across more than one comparison (non-negligible DIF criterion: R^2^>0.016 and p<.01) were candidates for exclusion. Samejima’s graded response model was also used to establish the item parameters of the final item banks [31]. Scoring for the finalized item banks involved the conversion of theta scores to a standardized *t* score (mean =50, SD =10).  *Preliminary Reliability and Validity Data.* We estimated IRT-based reliabilities at individual *t* score levels, which is derived from standard error estimates (i.e., reciprocal of test information). IRT-based reliabilities vary across the measurement continuum. A reliability ≥0.70 was considered acceptable [32]. Convergent validity was evaluated by examining the correlations between the PDAQ scores and the EDA-8 (criterion *r*’s ≥0.36) [33] and discriminant validity was evaluated by examining the correlations between the PDAQ item banks and measures that should be largely unrelated (i.e., autism traits subscales from the SDQ and child global health; criterion <0.36) [33]. Known groups validity was examined by comparing groups that are expected to show differences on the PDAQ. Specifically, we expected those with more uncommon behaviors (relative to those with more common behaviors; SDQ scores >17 versus ≤17), those with high demand avoidance (relative to those with low demand avoidance; EDA-Q scores >19 versus ≤19), those with a co-occurring autism diagnosis (relative to those without), those who were unschooled (relative to those who were homeschooled, in public school or in private school), and those with basic needs difficulties (relative to the sample mean), to have worse PDAQ scores than the specified comparator group; we also expected these groups to have elevated clinical impairment rates (i.e., scores that were >1 SD worse than the normative sample mean of 50) [34]. |
| Data sources/ measurement | 8* | For each variable of interest, give sources of data and details of methods of assessment (measurement). Describe comparability of assessment methods if there is more than one group | 8-9 | Study Measures  *The PDAQ Item Pool.* An initial pool of 67 questions was designed to evaluate the key behavioral traits of PDA including: survival drive for autonomy, equalizing behavior, masking behavior (two versions of oneself), cumulative nervous system activation (fluctuating and unpredictable), and need for constant 1:1 undivided attention. Specific item content was based on literature review, lived experience, experience working with families with PDA, and surveys that were conducted with 15 parents of PDA children or teens. These data were used to inform the conceptual framework upon which this measure was designed (Figure 1). Items were further refined by expert review (n=3 PhD-level scientists; 1 MA-level investigator; and n=2 BA-level professionals), evaluation of item literacy level (to ensure ≤6^th^ grade reading level),[19] translatability review (to facilitate a future translation into Spanish), and participant cognitive review (n=5) to ensure adequate content coverage and appropriate reading and comprehension levels. The final item pool was comprised of 60 items (Figure 2).  Participants also completed: 1) EDA-8 [15, 16], an 8-item parent proxy measure designed to assess the presence of PDA-specific traits in children; 2) the Childhood Autism Spectrum Test (CAST) [20], a 39-item parent proxy measure designed to assess the severity of autism symptoms in children; 3) the Strengths and Difficulties Questionnaire (SDQ) [21], a 25-item proxy-reported child behavioral screening questionnaire, 4) the PROMIS Parent Proxy Scale v1.0 – Global Health 7+2 [22], a 9-item parent proxy measure designed to assess a child’s physical, mental, and social health; and 5) demographic questions about themselves and PDA child or teen. |
| Bias | 9 | Describe any efforts to address potential sources of bias | 10 | Differential item functioning (DIF) was investigated to identify potential item bias (e.g., to identify items that might unfairly discriminate for or against individuals in a given group) [29] using R package LORDIF Version 0.3-2 [30]. DIF was evaluated by child age, child education, child school type, child sex, parent age, and parent education. Items with DIF across more than one comparison (non-negligible DIF criterion: R^2^>0.016 and p<.01) were candidates for exclusion. |
| Study size | 10 | Explain how the study size was arrived at | 11 | *Sample Size Considerations.* Sample sizes of 200-1000 have been proposed when using GRM, with larger sample sizes yielding more stable item parameter estimates [35, 36]. In addition, a minimum of 5 individuals are recommended for each item within an item pool [37-39]; with 60 items in our pool, a minimum of 300 participants would be needed. Furthermore, DIF analyses can be performed provided the overall sample size is ≥500 and there are at least 200 participants within each variable category tested.[40] |

Continued on next page

| Quantitative variables | 11 | Explain how quantitative variables were handled in the analyses. If applicable, describe which groupings were chosen and why | 9-11 | Analysis Plan  *Survey Development*. Analyses for the development of the PDAQ were conducted according to established standards and involved both classical test theory (CTT) and item response theory (IRT) [18]. We first evaluated the dimensionality of the item pool. The sample was randomly divided into two datasets: one for exploratory factor analysis (EFA) and the other for confirmatory factor analysis (CFA). EFA was used to explore potential factor structure among items. We explored the number of factors based on number of factors with eigenvalue > 1 and the scree plot (i.e., number of factors before the break in scree plot). EFA with a promax rotation with polychoric correlations was then used to examine the association among factors and items and to examine item loadings (criterion: >0.3). CFA was conducted using the weighted least square-mean and variance adjusted estimator with polychoric correlations [24]. Item sets were considered unidimensional, with their items retained in the item pool, when the following criteria were met: comparative fit index (CFI) >0.9; root mean square error of approximation (RMSEA <0.1 [25]; R2 >0.3; correlated error modification index <10; residual correlations <0.15 [26]). Samejima’s graded response model (GRM) was used to identify items with significant misfit (S-X2 / df effect size >3) [27] using IRTPRO (version 3.1.2) [28]. Differential item functioning (DIF) was investigated to identify potential item bias (e.g., to identify items that might unfairly discriminate for or against individuals in a given group) [29] using R package LORDIF Version 0.3-2 [30]. DIF was evaluated by child age, child education, child school type, child sex, parent age, and parent education. Items with DIF across more than one comparison (non-negligible DIF criterion: R^2^>0.016 and p<.01) were candidates for exclusion. Samejima’s graded response model was also used to establish the item parameters of the final item banks [31]. Scoring for the finalized item banks involved the conversion of theta scores to a standardized *t* score (mean =50, SD =10).  *Preliminary Reliability and Validity Data.* We estimated IRT-based reliabilities at individual *t* score levels, which is derived from standard error estimates (i.e., reciprocal of test information). IRT-based reliabilities vary across the measurement continuum. A reliability ≥0.70 was considered acceptable [32]. Convergent validity was evaluated by examining the correlations between the PDAQ scores and the EDA-8 (criterion *r*’s ≥0.36) [33] and discriminant validity was evaluated by examining the correlations between the PDAQ item banks and measures that should be largely unrelated (i.e., autism traits subscales from the SDQ and child global health; criterion <0.36) [33]. Known groups validity was examined by comparing groups that are expected to show differences on the PDAQ. Specifically, we expected those with more uncommon behaviors (relative to those with more common behaviors; SDQ scores >17 versus ≤17), those with high demand avoidance (relative to those with low demand avoidance; EDA-Q scores >19 versus ≤19), those with a co-occurring autism diagnosis (relative to those without), those who were unschooled (relative to those who were homeschooled, in public school or in private school), and those with basic needs difficulties (relative to the sample mean), to have worse PDAQ scores than the specified comparator group; we also expected these groups to have elevated clinical impairment rates (i.e., scores that were >1 SD worse than the normative sample mean of 50) [34]. |
| --- | --- | --- | --- | --- |
| Statistical methods | 12 | (*a*) Describe all statistical methods, including those used to control for confounding | 9-11 | Analysis Plan  *Survey Development*. Analyses for the development of the PDAQ were conducted according to established standards and involved both classical test theory (CTT) and item response theory (IRT) [18]. We first evaluated the dimensionality of the item pool. The sample was randomly divided into two datasets: one for exploratory factor analysis (EFA) and the other for confirmatory factor analysis (CFA). EFA was used to explore potential factor structure among items. We explored the number of factors based on number of factors with eigenvalue > 1 and the scree plot (i.e., number of factors before the break in scree plot). EFA with a promax rotation with polychoric correlations was then used to examine the association among factors and items and to examine item loadings (criterion: >0.3). CFA was conducted using the weighted least square-mean and variance adjusted estimator with polychoric correlations [24]. Item sets were considered unidimensional, with their items retained in the item pool, when the following criteria were met: comparative fit index (CFI) >0.9; root mean square error of approximation (RMSEA <0.1 [25]; R2 >0.3; correlated error modification index <10; residual correlations <0.15 [26]). Samejima’s graded response model (GRM) was used to identify items with significant misfit (S-X2 / df effect size >3) [27] using IRTPRO (version 3.1.2) [28]. Differential item functioning (DIF) was investigated to identify potential item bias (e.g., to identify items that might unfairly discriminate for or against individuals in a given group) [29] using R package LORDIF Version 0.3-2 [30]. DIF was evaluated by child age, child education, child school type, child sex, parent age, and parent education. Items with DIF across more than one comparison (non-negligible DIF criterion: R^2^>0.016 and p<.01) were candidates for exclusion. Samejima’s graded response model was also used to establish the item parameters of the final item banks [31]. Scoring for the finalized item banks involved the conversion of theta scores to a standardized *t* score (mean =50, SD =10).  *Preliminary Reliability and Validity Data.* We estimated IRT-based reliabilities at individual *t* score levels, which is derived from standard error estimates (i.e., reciprocal of test information). IRT-based reliabilities vary across the measurement continuum. A reliability ≥0.70 was considered acceptable [32]. Convergent validity was evaluated by examining the correlations between the PDAQ scores and the EDA-8 (criterion *r*’s ≥0.36) [33] and discriminant validity was evaluated by examining the correlations between the PDAQ item banks and measures that should be largely unrelated (i.e., autism traits subscales from the SDQ and child global health; criterion <0.36) [33]. Known groups validity was examined by comparing groups that are expected to show differences on the PDAQ. Specifically, we expected those with more uncommon behaviors (relative to those with more common behaviors; SDQ scores >17 versus ≤17), those with high demand avoidance (relative to those with low demand avoidance; EDA-Q scores >19 versus ≤19), those with a co-occurring autism diagnosis (relative to those without), those who were unschooled (relative to those who were homeschooled, in public school or in private school), and those with basic needs difficulties (relative to the sample mean), to have worse PDAQ scores than the specified comparator group; we also expected these groups to have elevated clinical impairment rates (i.e., scores that were >1 SD worse than the normative sample mean of 50) [34]. |
|  |  | (*b*) Describe any methods used to examine subgroups and interactions | 10 & 11 | Differential item functioning (DIF) was investigated to identify potential item bias (e.g., to identify items that might unfairly discriminate for or against individuals in a given group) [29] using R package LORDIF Version 0.3-2 [30]. DIF was evaluated by child age, child education, child school type, child sex, parent age, and parent education. Items with DIF across more than one comparison (non-negligible DIF criterion: R^2^>0.016 and p<.01) were candidates for exclusion.  Known groups validity was examined by comparing groups that are expected to show differences on the PDAQ. Specifically, we expected those with more uncommon behaviors (relative to those with more common behaviors; SDQ scores >17 versus ≤17), those with high demand avoidance (relative to those with low demand avoidance; EDA-Q scores >19 versus ≤19), those with a co-occurring autism diagnosis (relative to those without), those who were unschooled (relative to those who were homeschooled, in public school or in private school), and those with basic needs difficulties (relative to the sample mean), to have worse PDAQ scores than the specified comparator group; we also expected these groups to have elevated clinical impairment rates (i.e., scores that were >1 SD worse than the normative sample mean of 50) [34]. |
|  |  | (*c*) Explain how missing data were addressed |  | N/A |
|  |  | (*d*) *Cohort study*—If applicable, explain how loss to follow-up was addressed  *Case-control study*—If applicable, explain how matching of cases and controls was addressed  *Cross-sectional study*—If applicable, describe analytical methods taking account of sampling strategy | N/A |  |
|  |  | (*e*) Describe any sensitivity analyses | N/A |  |
| Results | | | | |
| Participants | 13* | (a) Report numbers of individuals at each stage of study—eg numbers potentially eligible, examined for eligibility, confirmed eligible, included in the study, completing follow-up, and analysed | 11 & Table 1 | A total of 707 parents of PDA children or teens participated in this study; see Table 1 for details. |
|  |  | (b) Give reasons for non-participation at each stage | N/A |  |
|  |  | (c) Consider use of a flow diagram | N/A |  |
| Descriptive data | 14* | (a) Give characteristics of study participants (eg demographic, clinical, social) and information on exposures and potential confounders | Table 1 |  |
|  |  | (b) Indicate number of participants with missing data for each variable of interest | N/A |  |
|  |  | (c) *Cohort study*—Summarise follow-up time (eg, average and total amount) | N/A |  |
| Outcome data | 15* | *Cohort study*—Report numbers of outcome events or summary measures over time | *N/A* |  |
|  |  | *Case-control study—*Report numbers in each exposure category, or summary measures of exposure |  |  |
|  |  | *Cross-sectional study—*Report numbers of outcome events or summary measures |  |  |
| Main results | 16 | (*a*) Give unadjusted estimates and, if applicable, confounder-adjusted estimates and their precision (eg, 95% confidence interval). Make clear which confounders were adjusted for and why they were included | 11-12 | PDAQ Survey Development.  Initial EFA results suggested five factors (Supplemental Table 1). Factor 1 included 25 items that covered behavioral traits of PDA (survival drive for autonomy, equalizing, externalized or “fight” behavior; one item cross-loaded on Factor 2 and one item cross-loaded on Factor 4). Factor 2 included 19 items that were focused on a child or teen’s access to basic needs (eating, sleeping, and hygiene; one item cross-loaded on Factor 1). Factor 3 included 6 toileting items. Factor 4 included two sleep items, and two items focused on the cumulative impact of nervous system activation (one of which cross-loaded on Factor 1); given the small number of items and the fact that demand avoidance for sleep was also conceptually covered by Factor 2, this factor was not given additional consideration. Factor 5 only included 3 items which focused on a fight response; given that 3 items is insufficient for a scale, no additional consideration was given to this factor.  We conducted a second EFA including only the items from the initial EFA that loaded on either Factors 1, 2, or 3. This second EFA (see Table 2) had: 1) 25 items for Factor 1 (“Behavioral Expression”; again covering externalized behavior, demand avoidance, and co-regulation); 2) 18 items for Factor 2 (“Basic Needs”; one of which had a higher cross-loading on Factor 1); and 3) 6 items for Factor 3 (“Toileting”). None of the remaining items were flagged for DIF for child schooling type, parent age, or parent education. Although 4 items were flagged for potential DIF for child age, these items were retained given that they did not also get flagged based on other demographic variables. A summary of this iterative process is shown in Supplemental Table 2.  CFAs were then used to confirm the essential unidimensionality of the remaining 20 Factor-1, 10 Factor-2, and 6 Factor-3 items, respectively (Table 3). Next, IRT parameter estimates for the 20 items in Factor 1 (Behavioral Expression) indicated slopes ranging from 0.88 to 1.86 and thresholds ranging from -6.04 to +3.24;the 10 items in Factor 2 (Basic Needs) indicated slopes ranging from 0.92 to 2.61 and thresholds ranging from -5.04 to 2.46; and the 6 items in Factor 3 (Toileting) indicated slopes ranging from 1.82 to 3.29 and thresholds ranging from -1.86 to 2.52 (Supplemental Table 3). Scores were converted to a *t* score (mean = 50, SD = 10) relative to the sample means. The final measure and scoring instructions are provided in the appendix. |
|  |  | (*b*) Report category boundaries when continuous variables were categorized | N/A |  |
|  |  | (*c*) If relevant, consider translating estimates of relative risk into absolute risk for a meaningful time period | N/A |  |

Continued on next page

| Other analyses | 17 | Report other analyses done—eg analyses of subgroups and interactions, and sensitivity analyses | 13 | Preliminary Reliability and Validity data. Based on expected IRT-based reliability, precise estimation (defined as reliability ≥ 0.7) could be produced between T-scores 20 – 80 for Factor 1 (Behavioral Expression), between 20 and 67 for Factor 2 (Basic Needs), and between 30 and 77 for Factor 3 (Toileting; see Figure 3 for the IRT-based reliability). The marginal reliabilities were 0.89, 0.86, and 0.89, for Factor 1, 2, and 3, respectively. Furthermore, the interrelationships among the PDAQ item bank scores and comparator measures were all consistent with the proposed hypotheses: there were moderate relationships between the PDAQ and EDA-Q scores supporting convergent validity, and there were generally negligible to small correlations between the PDAQ scores and the other prespecified discriminant validity comparators (Table 4); *note:* there were two exceptions for Behavioral Expression where there were low-moderate correlations with the SDQ Hyperactivity and Conduct Problems subscales. In addition, all groups expected to have higher (i.e., worse) PDAQ scores did (Table 5) with two exceptions (there was no difference for those that were in private school versus those that were unschooled for Toileting scores, as well as for those with less versus more uncommon behaviors for Toileting; both of these findings were in the expected direction although they did not meet conventional levels of significance). |
| --- | --- | --- | --- | --- |
| Discussion | | | | |
| Key results | 18 | Summarise key results with reference to study objectives | 13-15 | This paper describes the development of the PDAQ, a new 36-item measure of PDA-specific behaviors and basic needs impacts. It also provides preliminary reliability and validity data to support its clinical utility. This measure was developed in accordance with accepted measurement development standards, which included both CTT and IRT analytical approaches. Specifically, factor analyses supported three unidimensional sets of items that were devoid of item bias for child schooling, child sex, parent age, and parent education. In addition, psychometric analyses indicated that factor scores derived from the PDAQ exhibit very good internal consistency, are devoid of ceiling effects, demonstrated convergent and discriminant validity, and known groups validity.  The PDAQ includes three factors: Behavioral Expression, Basic Needs, and Toileting, all of which are scored on a *t* metric (*M* = 50, *SD* = 10), such that higher scores on each factor indicate more PDA-specific behaviors and impacts on basic needs. Given that *t* scores are standardized, this type of scoring approach aids in clinical interpretation. For example, parents of children with scores of 60 or above (those with scores that are ≥ one standard deviation above the mean) on any of the factors, are reporting that their child has more significant behavioral expression, basic needs impact or toileting impact (for Behavioral Expression, Basic Needs, or Toileting factors, respectively) that is worse than 83.9% of PDA children or teens. Furthermore, parents of PDA children or teens with scores of 70 or more (those with scores that are ≥ two standard deviations above the mean) are reporting behaviors, and basic needs impacts that exceed 97.9% of their PDA-specific peers.  Convergent validity and discriminant validity for the three factors was supported. As expected, the correlations were highest between each of the associated factors and the EDA-Q, and there were lower correlations (that were generally negligible to small) between the factor scores and the discriminant validity measures. Of note, although the Behavioral Expression score showed slightly higher correlations than hypothesized (there were low moderate correlations with hyperactivity and conduct problems as opposed to the proposed negligible to low correlations); this is not altogether surprising given that Behavioral Expression included items that overlap with both of those concepts. In addition, although the relationship between the toileting factor score and the EDAQ was higher than the correlations with the discriminant validity measures, it was weaker than expected (i.e., less than 0.36). In hindsight, this is not especially surprising given that none of the content on the EDAQ focuses on issues of toileting. In fact, further examination of the correlations among the three factors of the PDAQ (r = 0.49 between behavioral expression and basic needs, and r = 0.36 between behavioral expression and toileting, and r = 0.24 between basic needs and toileting) supports this premise. Specifically, the Behavioral expression of PDA, which is measured both by the PDAQ Behavioral Expression factor, and by the 8-items of the EDA-Q, each only have small correlations with the Toileting factor.  Findings also supported known groups validity; the PDAQ factor scores differentiated between groups as hypothesized. Specifically, scores were differentiated between those with more versus less common behaviors (note, there was only a trend for a significant direction for Toileting, but the means were in the correct direction), high versus low demand avoidance, and children with curriculum-based school versus unschooling. In addition, all three factors were able to differentiate between those with and without co-occurring autism diagnoses, such that those children with an autism diagnosis exhibited more demand avoidance behaviors than those without. Finally, PDAQ scores also differentiated between those with and without basic needs difficulty; those with reported difficulty had worse PDAQ scores for all three factors than those that did not. |
| Limitations | 19 | Discuss limitations of the study, taking into account sources of potential bias or imprecision. Discuss both direction and magnitude of any potential bias | 15&16 | Regardless, there are several limitations to consider. First, conceptually, the items that were designed to capture a more internalized expression of PDA did not survive the analysis, thus additional measurement development work targeting this behavioral profile is warranted. Even so, proxy assessments are not recommended for more internal states that are difficult to observe empirically, and thus this may be more appropriate for inclusion in a self-report measure with PDA individuals in a future study. This also raises a second limitation; the PDAQ was designed as a proxy, parent-reported measure. Future work is needed to develop a self-report option that can be used with children aged 8 and older. In addition, PDA is not a recognized diagnostic category in the United States, which precludes our ability to examine the sensitivity and specificity of this group in our largely U.S.-based sample. It also limits our sample to parents who *believe* that their child is neurodivergent with a PDA profile rather than parents whose children have been *diagnosed* by a provider as either having PDA or ASD. Furthermore, our sample was highly educated and had greater socioeconomic means than the general population; thus, generalizability to those with lower education and income levels is limited. Also, over half of the parents in this sample reported having an anxiety disorder themselves, a contextual factor that warrants additional study. Furthermore, because the parents participated anonymously and were the only ones to provide reports of PDA in their children, there is no way to verify the accuracy of the self-reported PDA behaviors of the children. Also, social media was used as the primary recruitment source for this study. These factors call for future research that relies on sources of recruitment that are not convenience based. |
| Interpretation | 20 | Give a cautious overall interpretation of results considering objectives, limitations, multiplicity of analyses, results from similar studies, and other relevant evidence | 15 | Taken together, the measurement development approach of the PDAQ, the preliminary reliability and validity of the scores from the PDAQ, and the inclusion of both a general PDA behavior factor and a basic needs impact factor, are strengths of the current measure. |
| Generalisability | 21 | Discuss the generalisability (external validity) of the study results | 16 | Conclusion  In summary, the PDAQ is the first measure of PDA using rigorous, contemporary measurement development standards and includes a focus on how PDA impacts a child’s access to basic needs, such as eating, sleeping, toileting, and hygiene, by capturing more than simple broad behavioral traits of this profile. The scores for this measure demonstrate strong preliminary psychometric properties. Ultimately, this new tool might be used to help aid clinicians and researchers in better understanding this profile in their consideration as a candidate for recognition in the DSM. Regardless, it can be used to better understand children with a PDA profile and can be used to evaluate treatment outcomes for these children. |
| Other information | |  | | |
| Funding | 22 | Give the source of funding and the role of the funders for the present study and, if applicable, for the original study on which the present article is based |  | N/A |

*Give information separately for cases and controls in case-control studies and, if applicable, for exposed and unexposed groups in cohort and cross-sectional studies.

**Note:** An Explanation and Elaboration article discusses each checklist item and gives methodological background and published examples of transparent reporting. The STROBE checklist is best used in conjunction with this article (freely available on the Web sites of PLoS Medicine at http://www.plosmedicine.org/, Annals of Internal Medicine at http://www.annals.org/, and Epidemiology at http://www.epidem.com/). Information on the STROBE Initiative is available at www.strobe-statement.org.
