# Supplementary material for: The Demand Avoidance and Autonomy Questionnaire (DAAQ): Development of a new measure of demand avoidance
Source: PLoS One. 2026 Nov 11;21(11):e0345827. doi: 10.1371/journal.pone.0345827 (PMC13754100; doi:10.1371/journal.pone.0345827)
Supplement: Supplementary file 9 [file pone.0345827.s011.docx]

**JUNE 2026 PLOS ONE PEER REVIEW.**

**Foreword.**

Thank you for the opportunity to complete this peer review. It is matter I have been reflecting upon for some time. Those reflections contributed to other factors exacerbating the amount of demand-avoidance I was expressing towards this peer review. I apologise for it taking so long. One of the ways, my demand-avoidance upsets me is how often impacts working relationships.

I acknowledge that a substantial amount of effort and attention has gone into the revised manuscript. I am grateful for that. I still have a number of concerns, which have been raised by the revisions. The standard I am holding the manuscript to, is comparable to recent systematic reviews (Company and Rostella 2026; Kildahl et al 2021), on raising concerns over PDA’s evidence base.

I have given this one proof read. I apologise, if anything is unclear. If any point needs clarification, please liaise through the journal.

I have quoted a case study “Kieran” from Soppitt (2021, pp300-302) at the end of this review for everyone’s ease of reference. Kerian was put forward by Soppitt as an example of what PDA looks like. This case study is pertinent to many of my concerns of the manuscript. Soppitt discusses how they model as intersection of various co-occurring difficulties, see this diagram from Soppitt (2021, p299):

Soppitt identifies PDA due to it describing a person’s behaviours. PDA itself is not the explanatory factor for Kieran’s behaviours, for at least some of them, are clearly trauma related. I searched for “*toilet*”, “*clean*”, and “*hygiene*”, and found no results. It is reasonable to question if Kieran would meet threshold for PDA, especially if one applies a heightened drive for autonomy, as a explanation for their behaviour. Furthermore, the items used to identify PDA include features which contradict typical autism understandings, such as “*He is socially manipulative to avoid demands*”, when Autistics are meant to lack the empathy to do this. This outlook is also shown in more recent literature, e.g., Chetcuti et al (2026). The point here, is that one can reasonably question if autism has expanded to include PDA features, or if PDA represents features assessed for in autism (reviewer finds it ironic O’Nions needed to create PDA items for the ADOS-1, as ADOS does not assess for features described by PDA, see O’Nions 2013, chapter 8). Reflective questions: How does Kieran’s PDA compare to PDA’s model in the manuscript? How is Kieran likely to interact with the tool’s items?

These points will matter to the review.

**Notes on the manuscript.**

1) I have searched for it, and I apologise if I missed it. The hypothesis of the significantly higher threshold from avoidance to “*ordinary*” demands, instead of “*everyday*” demands (Company and Rostella 2026), needs to be mentioned when discussing how heightened drive for autonomy has become proposed to explain PDA.
2) The discussion on a heightened drive for autonomy needs to mention the hypothesis heightened drive for autonomy is due to a rhetorical repositioning of PDA as an ASD profile (Company and Rostella 2026).
3) Most predictions on PDA populations are based on Newson’s descriptions, such as (Gillberg 2014). This needs to be explicitly stated and considered when justifying the tool’s underpinning model. I apologise, if I missed this, but the similarities, and differences of the tool’s underpinning model, to Newson’s descriptions need to be mentioned for ease of reference.
4) Company and Rostella (2026) critique of the EDA-Q should be included in the literature review.
5) The shift using a formulation, instead of a descriptive diagnosis for PDA needs to be mentioned. See the Kieran case study as example. Phil Christie, 5≥ years ago (e.g., see their talks in 2016-2019), in their various conference talks, (accurately) mentioned how psychiatric categories are based on reliability over validity, and proceeded onto reasons for using a diagnosis in research and practice settings. The hypothesis the change to a PDA formulation, from a diagnosis is due to a rhetorical repositioning of PDA as an ASD profile (Company and Rostella 2026). This matters as using a heightened drive for autonomy and equality, is used as an explanation for a person’s behaviours, instead of describing them. Changing PDA from a descriptive category to an explanatory one is MASSIVE change in PDA’s ontology, when there is insufficient evidence to warrant it. This topic needs to be discussed, even if manuscript disagrees with my views on the topic.
6) A appreciate changing the tool’s name. I am struggling to see how the current name is accurate, or helpful. This logic is suitable A+B≠A. I say this because it is called demand avoidance and autonomy questionnaire. Yet, the heightened drive for autonomy and equality is meant to be model underpinning the tool, how it can it be both (i.e., demand-avoidance itself is broader than a heightened drive for autonomy, or a heightened drive for equality. Furthermore, a heightened drive for autonomy, is only meant to be part of the causes of PDA’s demand-avoidance, according to the tool’s underpinning model)? I am not entirely surely that the model, is a model for heightened drive for autonomy and equality. The manuscript describes a drive for autonomy more than a heightened drive for equality. How well is a heightened drive for equality captured in the tool’s items? If the tool significantly assesses for a heightened drive for equality, then the tool’s name should be changed to reflect this. If the tool does not, then conceptual model underlying the tool should be updated to reflect it is not sufficiently capturing a heightened drive for equality, or be this should be noted in the limitations.
7) Equality, as a concept is highly subjective. Values behind equality can rapidly change, and are transient; one only has to look at debates around right to vote, and/ or slavery, for different values for equality. If a heightened drive for equality conceptually underpins this tool, then the subjective nature of equality needs to be addressed.
8) I presented an open ended question, for potential causes of Autistics scoring higher for the tool’s 3 subscales, to see if manuscript may consider environmental and cultural factors might contribute to these results. How poorly treated Autistics are by Global North cultures, is a large factor behind the atrocious mental health issues found in the Autistic population, and suicide related problems (Autistic scholars discuss these points, arguably Monique Botha’s work is amongst most important). Most people, who are as distressed and traumatised as many Autistics are, are likely to score highly on at least 1 of the tool domains. Additionally, it seems reasonable to question, if the result is at least partly caused by how PDA is often modelled as a “*Profile of ASD*”, identified in Autistics, and researched in Autistics, that this can potentially bias the tool towards Autistics. It is worth noting the hypothesis heightened drive for autonomy is due to a rhetorical repositioning of PDA as an ASD profile (Company and Rostella 2026).
9) Unless I am missing the citations in the paragraph discussing the study’s hyperactivity and conduct issues results, they need to be situation in broader literature on the topic.
10) I apologise, if I missed it. May the words “*prejudice*”, and “*stigma*” be used in the manuscript? Even it they are not used to discuss potential risks of attributing PDA to an individual, some have argued a heightened drive for autonomy, is less stigmatising than using “*Pathological Demand Avoidance*” term.
11) One of the most replicated results in PDA studies, is how behaviours assessed for by PDA tools decrease as a CYP grows into adulthood. How does this study’s results compare to those results?
12) Thank you mentioning the Attachment disorder hypothesis.
13) Sample size is an indication of the passion for the PDA out there. Also reflects many other PDA studies.
14) L187-189: It is obvious, that researchers are going to continue to hold PDA to typical standards, which are there to protect people (Company and Rostella 2026). An alternative outlook to families feeling unseen etc, is to point to those exposing the vulnerable persons to PDA’s controversies, by prematurely reifying PDA. This is a factor why Green et al (2018), suggest utilising features from co-occurring difficulties to model PDA.
15) L191. “*broader autism spectrum presentations,*”. This is a contested opinion. Yes, many view PDA to be a “profile of ASD”, but this could be due to a hypothesised rhetorical repositioning of PDA as an ASD profile. At least before 2015, PDA’s literature tended to explicitly note which features make it problematic features contradict autism understandings. That phrasing needs to be changed.
16) L214-218. This is conjecture. As pointed out in the first review. There are good grounds to question in PDA’s demand-avoidance is intrinsically to “*everyday*” demands, such as those associated with toileting etc. Again good reasons to question, if avoidance to demands, like hygiene matters should be associated with PDA. It is possible to gain statistics of use of EDA-Q. The manuscript needs to provide the numbers/ percentages of studies utilising EDA-Q, in Kildahl et al (2021), Company and Rostella (2026), and Haire et al (2024) respective reviews. It might also be worth noting that not all datasets including the EDA-Q have been analysed; from my understanding Help4Psychology have not had their EDA-Q data analysed for potential correlations with any tools they used as part of their assessments, such as the Short Sensory Profile. It might be worth contacting Help4Psychology, if this is still the case, and possibly comment on the topic in the manuscript.
17) L265. Notes purpose of the study was for the tool to capture PDA specific features. Considering the literature generally notes PDA lacks any specific features; this objective needs to be explicitly addressed in the literature review. It is worth stating PDA does not need to have any specific features for it to be included in the psychiatric manuals, as most categories and characteristics operate as continua, and overlapping each other. Most categories in the DSM lack specific causal mechanisms.
18) L292. “*An initial pool of 67 questions was designed to evaluate the key*”, implies those 67 items describe PDA, which as the manuscript notes, may not be the case. Please could it be changed to something like “*An initial pool of 67 questions was designed to evaluate the hypothesised key*”.

**Kieran PDA case study from Soppitt (2021, pp 300-302).**

Assessment and detection of PDA

Case Study—1

**Kieran (anonymised name)**

Kieran was born uneventfully and was deemed a ‘model child’. He struggled with weaning but otherwise seemed quite content. However, there was a dramatic change and Kieran was on the go all the time from his first steps at the age of 15 months. He seemed to change, becoming irritable and started to demonstrate challenging behaviour from the age of 18 months. Kieran displayed major behavioural meltdowns. He was physically aggressive to his parents requiring restraint on occasions to prevent harm. He was into everything and did not sleep properly through the night until he was nearly five. He required a hospital admission for several months which meant he spent less time with certain family members and some attachment disruption, aged three. He has been very clingy ever since.

Kieran was assessed as having a high verbal IQ of 140 and at least a high average performance IQ. Kieran was late to speak, putting two words together at 30 months. Kieran spoke fluently by the time he went to Reception, aged four. His parents were exhausted and found that their extended family were not keen to look after him. By the start of primary school, Kieran had fractured his arm from jumping off trees and heights. At the age of six, after some time on a waiting list, Kieran was assessed by his community paediatric ASD pathway and found to meet DSM-5 criteria for ASD with impaired social communication and interaction and rigidity and repetitive patterns of behaviour. They later diagnosed ADHD with school and home observation.

The school observation indicated (his class teacher was off sick, so the class TA was leading the class) that he was extremely anxious; he struggled to organise himself. Kieran hummed loudly to assist his focus in a noisy class; he did not appear to hear questions or instructions in class but did so in a 1:1 setting. He was seen to become more restless the longer he had to sit. He was rigid with the rules and had to engage in pushing games or firm pressure through leaning whilst waiting in the queue. He was often fiddling with the pen lid whilst he worked and was constantly seeking movement through fidgeting, fiddling, jumping and spinning when he was up. He was vestibular seeking. In a 1:1 setting, Kieran was very attentive. He can be aggressive when anxious and he will kick, punch, bite and run away, use weapons like sticks and he can run in front of cars with no appreciation of danger. The Specialist Teacher reported that Kieran has severely inflexible thinking due to ASD. He struggles with perspective and dwells on the negative. **His strategies for dealing with emotions are limited to fight or flight reactions.**

When Kieran is anxious, he freezes and is not capable of showing his potential or even learn. Triggers for meltdowns include some trauma reminders. Trains are a trigger for him since he was separated in the Underground and made it to the next stop aged eight. This fear has been very prominent since then. He dislikes even the word ‘train’ being said. He rarely cries, maybe several times a year. He managed to hold it together until the train going home, whereby he sobbed for ages. He says he will never go on a train again. Perhaps he will manage this on his own when he is older as there will be no separation anxiety from a key carer.

We discussed Wing and Gould's criteria as follows (DISCO 11) Yes/No (Y/N).

1 **Unusually quiet and passive in infancy** (Y). He did not talk until he was aged two and a half but then managed complete sentences and could name every breed of dinosaurs.

2 **Gross and fine motor clumsiness** (Y). He avoided buttons before. Laces are tricky. His fine motor skills are poor. He is accident prone.

3 **Communicates through a doll, puppet, toy animal etc.** (Y). Kieran has communicated through different voices; he uses different voices. One is a baby voice when anxious and a demon voice - through gritted teeth; it is a totally different voice for a couple of sentences and then he will break something.

4 **Lacks awareness of social hierarchy** (Y). He treats everyone the same regardless of position.

5 **Rapid changes from loving to aggressive** (Y). He presents with a 'Jekyll and Hyde' presentation and can turn apparently for minor reason.

6 **Bossy and domineering** (Y).

7 **Repetitive role play more than usual pretence** (Y). It was more than usual pretence. He often role played being a cat and would drink milk from a saucer and communicate for hours like a dog with parents and brothers. He will spend time with his figures, and he re-enacts his day using his figures.

8 **His hands seem limp for unwelcome tasks** (Y). He uses every trick in the book to avoid complying with requests not of his choosing.

9 **Repetitive questioning -** about anything (Y). He will repeat the same question for an hour if it is the answer he does not want.

10 **Obsessed with a person real or fictional** (Y). E.g. one person at a time, real and fictional. He will constantly watch a particular You Tuber and try to mimic them and collects things and knows everything about this one person. He would like to stalk them if he knew where they lived.

11 **Blames others for his own misdeed** (Y). He blames others in the heat of the moment, but later can be full of remorse and he punishes himself so badly.

12 **Harassing others** (Y). He will try and bully and will try and find someone and will wreak revenge rather than tell an adult about his perceived injustice. If someone breaks the rules, he will target them when he is more reflective. He told a teacher he has four children on rotation that he will keep punishing as they have broken the rules in a game where they cheated.

13 **He is socially manipulative to avoid demands** (Y).

14 **He engages in socially shocking behaviour** (Y). He can press buttons to get a reaction; he will try and pick on a weak point of a person, then he may resort to shove or take something of theirs or hide it or run away with it. He will pick on people in sport and he lacks patience - he dislikes standing around.

15 **Has his own moral code and seems above the rules** (N). He consistently applies rules to himself - the letter of the law and to others unless very upset. He may borrow through manipulating - he will be cute, so he is given what he wants. He does not steal. Kieran has 14/15 PDA features according to Wing and Gould within his ASD diagnosis.

**Reference.**

Soppitt, R. (2021). Pathological/ Extreme Demand Avoidance (PDA/ EDA). In: Peer, L., & Reid, G (Eds.), *Special Educational Needs: A Guide for Inclusive Practice* (pp. 296-314). London, Sage Publications Limited.
