# Supplementary material for: The Demand Avoidance and Autonomy Questionnaire (DAAQ): Development of a new measure of demand avoidance
Source: PLoS One. 2026 Nov 11;21(11):e0345827. doi: 10.1371/journal.pone.0345827 (PMC13754100; doi:10.1371/journal.pone.0345827)
Supplement: Supplementary file 10 [file pone.0345827.s012.docx]

**Response to Reviewers**

**Key points.**

This manuscript should be published, if it acts upon the feedback provided.

There seems to be nothing technically wrong with the manuscript in its present iteration. It is an interesting and novel study. I major concerns over the potential harm this manuscript could do, if published in its present state.

**Thank you for this positive feedback.**

**Observations on the Manuscript.**

The study was an international study, in terms of focusing in English speaking Global North, and representing the views of some PDAers. It remains to be seen if this tool represents to views of PDAers from non-English speaking countries, in particularly countries from the Global Majority, which have diverse cultural values and expectations.

**We agree with this point and now include this as a study limitation.**

It is unclear if tool represents the views of both non-speaking PDAers, and PDAers with co-occurring Intellectual Disability (I could not find any suitable mention on this topic, or indicators for this population of PDAers in the sample).

**Thank you for this feedback. We did collect parent-reported developmental delays for the child with PDA. We have now added this information to Table 1.**

There is no consensus over how to identify PDA, and so there is diverse variation in the presentations of PDA identified. Some PDA advocates claim PDA is over identified (Who is to say, which approach to PDA has any more merit than another?) Ironically, some PDAers avoid the demands “Pervasive Drive for Autonomy” community, how well are their views captured unlikely to be captured by the methods of this study. Final point, is that some PDAers, such as Damian Milton, and Richard Woods, disagree with the “Pervasive Drive for Autonomy” conceptualisation of PDA, and instead prefer other terms, such as “Rational Demand Avoidance”. This should be reflected in potential limitations of the study, and reflected in suggestions for the tool’s use.

**We have now added discussion about the fact that there is no consensus definition or consistent terminology for referencing PDA to the introduction section.**

I am unsure if the construct of PDA in this manuscript is the same put forward by Newson, and later knowledge derived from Newson’s work. For instance, The study states its deliberately focused on toileting and hygiene difficulties of PDAers. I accept that this a problem for some PDAers. Toileting and hygiene issues discussed in the manuscript are sufficient that they should have been picked up key previous PDA scholarship before this. These issues should be problematic enough to be brought up by caregivers, and others around the PDAers during the clinical assessment process. The toileting and other issues should be collected as part of a comprehensive assessment for autism, or other Neurodevelopmental category, if only for excluding other explanations for an assessee’s behaviour.

**Thank you for requesting this clarification. Our conceptualization builds upon Newson’s work, but it is not fully aligned with it. We have added text in the introduction that highlights the ways in which our conceptualization overlaps and the ways in which our conceptualization differs from Newson’s original framework.**

Kildahl et al (2021) noted that Newson’s PDA conceptualisation is a mess, with many assumptions. It was this as Newson and colleagues including any significant features they observed in their PDAers. Searching Newson et al (2003) and supplementary notes using: “clean”, “hygiene”, & “toilet”, only yielded 1 instance, relation to obsessions on “over cleanliness/slovenliness” (Newson et al 2003, 598). The same 3 search terms were used on O’Nions et (2016a) DISCO paper, and its supplementary files and produced 0 results. This result was replicated in O’Nions et al (2014a) EDA-Q development paper, and O’Nions et al (2018) qualitative examination of ASD+PDA. A 22 item semi-structured interview used to assess for PDA from before 2015, does includes mentions of urinating in public in terms embarrassing and socially shocking behaviour. Considering the tools assesses for serious features, including self-injury. If toileting issues were present, they should be included in the tool. Should toileting issues be associated with PDA? Is it reflective of PDA? Are associating toileting and hygiene issues with PDA, likely to contribute to stigma and/ or prejudice experiencing by PDAers?

**This is an excellent point. You are correct in that these factors were not a part of Newson’s original framework, and this is one way in which our conceptualization differs from hers. As noted above, we now clarify how our theory compares to Newson’s and provide support for including this additional component in our framework. We are also working on a separate manuscript that further elucidates this component in our framing of PDA.**

Another reason to question if how PDA is conceptualised in this manuscript is significantly similar to Newson’s, is its relationship to hyperactivity and conduct problems. Previous research indicates that PDA features are predicted by hyperactivity and conduct problems (e.g., Egan et al 2020, Green et al 2018a pp.6-8). Anna Goodson 2018’s doctoral study indicated that about a third of PDA features are correlated with Executive Functioning issues (which are typically associated with ADHD). The study should have replicated other previous results. I suspect that if the full EDA-Q was used, instead of the EDA-8, at least for the EDA-Q, the predictive relationship between hyperactivity and conduct problems, and PDA, would been replicated. There should be stronger correlations between the tool and the hyperactivity and conduct scales on Strengths and Difficulties Questionnaire (SDQ), if how PDA is conceptualised in the manuscript is significantly similar to previous conceptualisations of PDA. Please can the authors reflect upon and discuss why there is a difference between the tools correlation with hyperactivity and conduct scales, which is different to previous PDA research.

**We again agree with your point, and we believe that differences in our framing of PDA may account for these discrepancies. We now raise this issue in the introduction section and note our use of the EDA-8 as a study limitation.**

A third factor consider is, the wording used to describe the nature of the demand-avoidance. Newson and most later PDA research, describe the avoidance as “obsessive” in nature, and to “ordinary” demands, not “everyday” demands, such as basic needs described in the manuscript. One can argue that the difference between “ordinary” demands, and “everyday” demands is crucial, with “ordinary” demands, include things like going to work, to change activity at school, which can be perceived as a level of functioning above toileting, and hygiene. It seems reasonable to question, if PDA described in this manuscript has a higher threshold, and thus excludes more cases than other PDA conceptualisations.

**As mentioned above, we have reframed the introduction and now explicitly discuss these points.**

Beyond these concerns over how representative is the tool, for most PDAers, and it’s similarities to other PDA models. There is substantial concerns over the prejudice and stigma caused by attributing PDA to individuals. Particularly, notable concerns over the use of the “pathological” descriptor with PDA. Elizabeth Newson disliked the term, and others have repeated such views, like Judy Eaton said “pathological” descriptor is derogatory, and instead proposed “Extreme Anxiety” (Eaton 2018). The depth of unease with the “pathological” descriptor is represented by there being about 20 alternative names in print for PDA. The prejudice and stigma originating from language around PDA matter, for example, who would wish to provide an educational placement to child, or young person, described as having an avoidance “ordinary”/ “everyday” demands? There accounts of CYP being attributed with PDA being denied educational placements due to their PDA attributions. The issues surrounding the “pathological” descriptor need to discussed in the manuscript, and its use in the tools name needs to be justified. If it cannot be, the tool’s name should be changed.

**We fully agree with these points, but we elected to use “PDA” in this manuscript to align with how it is referenced in the published literature. That said, we have revised the name of our new measure to the Demand Avoidance and Autonomy Questionnaire (DAAQ) to better reflect our conceptual framing of PDA and remove reference to pathological.**

I have significant concern over the potential stigma and prejudice this tool can add to PDAers, and Autistics through the addition of the 2 new scales, and nature of the difficulties being described in its items (similar concerns are expressed elsewhere over other PDA tools, due to assessing for problematic features such as, lying, stealing, bullying, and various violent acts etc – see Woods 2024). While the manuscript notes, there is disagreement over if PDA is a “Profile of ASD”, it is often portrayed as such by many of its advocates. There are risks of associating the toileting, hygiene, and other difficulties with autism, can contribute to the stigma and prejudice associated for all Autistics. This matters as DSM-4 autism subtypes of Asperger’s Syndrome, Autistic Disorder, and Pervasive Developmental Disorder-Not Otherwise Specified (PDD-NOS), were partly excluded from the DSM-5, to reduce stigma for all Autistics (Happe 2011; Kapp & Ne’eman 2019). By creating a PDA tool to assess for basic needs such as eating, sleeping, toileting, and hygiene, one can argue that how PDA is conceptualised in this manuscript, that PDA is being modelled as a functioning category. There extant debates over the use of functioning categories in autism, with the use proposed subgroup of “Profound Autism”. The manuscript describes PDA’s difficulties as “profound”. These debates need to be directly acknowledged in the manuscript, and how they relate to how PDA is conceptualised in the manuscript should be discussed, especially relating to prejudice and stigma.

**Thank you for this feedback. We appreciate the concerns raised, but we also believe that these difficulties—regardless of whether they are framed as ASD-related—do appear to present in these children. We hope that renaming our measure to be more symptom-specific rather than “syndrome”-specific partially addresses these concerns and helps mitigate potential downstream stigma. That said, whether these difficulties persist into adulthood is an empirical question; accordingly, we now note this in the Discussion in the hope that it further addresses these concerns.**

It is a choice to conceptualise PDA as a “Pervasive Drive for Autonomy”, and to aim for PDA to have specific features. Generally, PDA literature acknowledges there are no specific features to PDA. DSM-5 acknowledges that most features represented by diagnostic categories described within lack specific features, and they present as continua within human population. This results in most diagnostic categories overlapping, with other categories in the DSM-5. It should be possible to conceptualise PDA as a Neurodevelopmental disorder at lower diagnostic thresholds, like ADHD, which only needs to be expressed in at least 2 settings (one in social interactions), and be consistently expressed by the age of 12. Some PDA advocates claim the EDA-Q can provide false positive results (see PDA Society’s 2022 document on assessing for PDA). Requiring PDAers to conform to “Pervasive Drive for Autonomy”, risks stripping support packages and possible sense of self understanding, from people who have been attributed with PDA that do not conform to “Pervasive Drive for Autonomy”. Some PDA advocates claim PDA is over diagnosed. It becomes a self-fulfilling prophecy, if one chooses to model PDA with a high exclusion rate. One could argue it, can be seen as an act of gatekeeping.

**Although this is not a focus of the present article, one of our broader aims is to encourage further study of this symptom profile, including consideration by the DSM. DSM proposals require a clear rationale and historical context for the change, as well as discussion of potential negative consequences and arguments against the change, and an overview of significant controversies or disagreements among researchers and clinicians. In addition, for a topic to be considered, it is important to document inconsistent clinical terminology (e.g., “demand avoidant profile,” “ASD with extreme anxiety-driven avoidance,” “low tolerance for demands”) and to explain how adopting a more consensus-based term (whether PDA or another label) could standardize language and reduce diagnostic ambiguity. These considerations are central to our work as part of a broader research program aimed at clarifying core symptoms and delineating how this profile overlaps with, and/or is distinct from, autism—prerequisites for evaluating whether it is best conceptualized as a standalone diagnosis or as an autism-related profile.**

I cannot support the use of this tool in assessing for PDA for the reasons outlined in the above paragraphs. considering questions over how representative it is of majority of the PDAer population. Questions of it is similar construct, as described in earlier PDA scholarship. Potential harm it can cause from extra prejudice and stigma. EDA-Q which was developed in similar circumstances to the tool in this manuscript, and is advised against using the EDA-Q in PDA assessments. Additionally, we need to be sure that knowledge derived from PDA research is applicable to all PDAers. Please can the authors reflect upon these points, and discuss it in the manuscript.

**Thank you for these thoughtful comments. We have now incorporated these points into the manuscript. We reference the ongoing debate in the field and explain why we believe this new measure can help move the literature forward. Specifically, we describe the measure as one step in a longer process to evaluate how well these domains represent PDA as we currently conceptualize it, while also generating data that may identify where our conceptualization is not supported. We also note that we intentionally selected an item-banking approach because it enables the addition or removal of items and subsequent recalibration, allowing earlier versions of the measure to be directly mapped onto later versions. Given the lack of consensus regarding PDA, we position the measure as a research tool: it may yield clinically meaningful information, but it is not intended to function as a diagnostic instrument. We have clarified this in the manuscript as well.**

Ironically, the paper notes a lack of use of the EDA-Q in PDA studies, and this study only uses the EDA-8. There is at least one instance of the EDA-Q being referred to as the EDA-8 on L351. It is unclear why the full EDA-Q was not used, when only the EDA-8 was. The full rationale for using the EDA-8, instead of the EDA-Q should be provided in the manuscript. Typos of the EDA-Q, should be changed to the EDA-8, and vice versa.

**We now include this rationale as a study limitation and have corrected the discrepancies in the manuscript.**

The manuscript should have a more in depth discussion of the EDA-Q, including the limitations observed in the PDA literature (Woods 2020, commenting on Stuart et al 2020, collated some of the critique of EDA-Q). Why not revise the EDA-Q to assess for access basic needs, such as eating, sleeping, toileting, and hygiene? Why not adapt the EDA-Q for self-assessment for under-18s, particularly as it should be less of a demand than a tool with 36 items in this tool? Please can the authors reflect upon, and discuss these points in the manuscript.

**Thank you for this feedback. The EDAQ was developed using classical test theory, whereas our tool was developed using item response theory, which offers advantages in administration, adaptability, and measurement precision. We now include additional discussion of the EDAQ’s strengths and limitations, as well as the differences between these development approaches. We hope these additions—along with our expanded discussion of how our theoretical framework differs from the EDAQ—address these points.**

In my view it should be interesting if the full 30 candidate items of the EDA-Q was used in this study as it includes items to assess for other reported aspects of PDA, like developmental features and 2 items indicative of social communication issues. For reference these are the 4 items removed in O’Nions et al (2014a, supplementary file):

- Has difficulty reading body language, facial expression
- Slow to process or respond to questions/ comments
- Able to take responsibility for misbehaviour
- Floppy as an infant

Please could the authors reflect upon, and discuss if future research should be conducted investigating potential relationships between the tool, and the 30 candidate items of the EDA-Q?

**Thank you for this suggestion. We used the EDAQ-8 to obtain preliminary convergent validity evidence without unduly burdening participants. We agree that future work should compare EDA-Q with the DAAQ, and we now note this as a limitation and a direction for future research in the manuscript.**

For purposes of the literature review, off the top of my head, these are the other extant tools used in PDA research/ assessment.

- Autism Diagnostic Observation Schedule-1 PDA items (O’Nions 2013).
- Autism Diagnostic Observation Schedule-2 Suggested Scoring (Eaton & Weaver 2020).
- Autism Spectrum Screening Questionnaire (ASSQ)-Revised Extended Version (ASSQ-REV) (Kopp & Gillberg 2011).
- Coventry Grid Interview plus Pathological Demand Avoidance (CGI-PDA) (Eaton et al 2018).
- Extreme Demand Avoidance-Questionnaire (EDA-Q) (O’Nions et al 2014a).
- Extreme Demand Avoidance Questionnaire for Adults (Egan et al 2018).
- Original 15 Diagnostic Interview for Social and Communication Disorders (DISCO) 15 (Gillberg et al 2015).
- Revised Diagnostic Interview for Social and Communication Disorders (DISCO) PDA 11 items.
- Semi-structured 22 Item semi-structured interview (O’Nions 2013; O’Nions et al 2018). I have scoring for rules for this tool from Elizabeth Newson Centre. If possible, the tool should be attached for authors own reference.

The ASSQ-REV was also used to study children. Haire et al (2024) scoping review for investigating PDA in children and adolescents seems relevant to the manuscript. The manuscript should be updated to reflect this.

**Thank you for this feedback. Because our goal is to develop a parent-report questionnaire rather than an interview-based assessment, we have focused our literature review on existing parent-report survey measures (rather than diagnostic interviews). That said, we also note how the DISCO has been used to evaluate a PDA-related behavioral cluster.**

Other alternative hypotheses for PDA include, PDA is a form of Attachment Disorder (Christie 2007 editorial; Flackhill et al 2017), & Personality Disorder (Christie 2007 editorial). There are PDA cases diagnosed independent of autism. Liz O’Nions PhD thesis has a few. Chapter 8 has 3 diagnosed under Conduct Problems, and the Qualitative study contains one diagnosed with Attachment disorder and scored 1 on the ADOS. The manuscript’s alternative hypotheses for PDA, should be updated to include as a form of Attachment Disorder, and Personality Disorder.

**Thank you. We have added these points as requested.**

Final specific comments.

L372-375. Why are Autistics expressing more demand-avoidance features than others?

**We do not yet know. We hypothesize that PDA may represent a profile within autism. Alternatively, it may be that demand avoidance is a broader symptom that is exacerbated in neurodivergent individuals due to processing differences and related challenges. Future work is needed to clarify these possibilities. We have added these points to the discussion.**

L262+L3673. If autism a co-occurring construct to PDA, then logically PDA cannot be a “Profile of autism” (A+B≠A). Perhaps, the manuscript can be amended to reflect this fallacy in the logic of PDA?

**We have revised the text as requested to note that this presentation may reflect a profile within autism, or alternatively, a distinct profile.**

L391-393. Originally, PDA was attributed as a standalone category. From around 2015, PDA was mainly attributed as dual ASD+PDA. Perhaps, it a more accurate phrasing is autism with, and without PDA formulations. The “PDA Profile of ASD” lobby tends to describe attributions as “formulations”, not “diagnoses”. This may be different outside of the UK. Please can the authors reflect upon this, and a suitable note to the manuscript on this point.

**As mentioned above, we have reframed the text as requested to introduce the possibility that this may be a profile of autism, or that it may be unique and have included discussion around how future work will be needed to better understand this.**

Also likely that the caregivers in the sample had predefined notions of PDA could be, and may have biased the study’s results (Kildahl et al 2021). Similar risks of bias, as other PDA studies seem to be present. Please can this be reflected upon, and discussed in the manuscript.

**As noted above, we have reframed the text as requested to introduce the possibility that this may reflect a profile within autism, or alternatively, a distinct profile, and we have added discussion highlighting the need for future work to clarify this.**

L396-397. Lauren Good’s (2016) Doctoral thesis may be of assistance here.

**We now reference her doctoral thesis in the discussion.**

PDAers might respond to CAST, comparable to Autistics, but for different reasons than Autistics do (O’Nions et al 2014b, p541). This needs to be reflected upon and discussed in the manuscript.

**We agree and have adjusted the text to clarify this point.**

Critique of PDA by both Milton and Woods are alluded to in the manuscript through citation of Moore (2020). They should be explicitly recognised as PDAers in the manuscript and cited, like their PDAer peers.

**Thank you. We have added these citations.**
