# Supplementary material for: The Demand Avoidance and Autonomy Questionnaire (DAAQ): Development of a new measure of demand avoidance
Source: PLoS One. 2026 Nov 11;21(11):e0345827. doi: 10.1371/journal.pone.0345827 (PMC13754100; doi:10.1371/journal.pone.0345827)
Supplement: Supplementary file 11 [file pone.0345827.s013.docx]

**Response to Reviewers**

**Reviewer #1:**

1) I have searched for it, and I apologise if I missed it. The hypothesis of the significantly higher threshold from avoidance to “*ordinary*” demands, instead of “*everyday*” demands (Company and Rostella 2026), needs to be mentioned when discussing how heightened drive for autonomy has become proposed to explain PDA.

Thank you for referencing the Company and Rotella article and noting the shift from “ordinary” to “everyday” demands. We have added text acknowledging Newson’s original use of the phrase “ordinary demands” and how several organizations have extended this framing to encompass “everyday” or “common” demands, including basic self-care activities (i.e., hygiene, eating, sleeping, toileting).

We further emphasize that avoidance of these demands is a cornerstone of our proposed definition of PDA that differentiates the new DAAQ measure from existing measures that do not assess functional impact on basic needs.

We also now include discussion of how PDA has come to be associated with heightened drive for autonomy, and clarify that autonomy-centered PDA accounts propose – but have not established – that ordinary demands may be experienced as disproportionately threatening to autonomy or control.

2) The discussion on a heightened drive for autonomy needs to mention the hypothesis heightened drive for autonomy is due to a rhetorical repositioning of PDA as an ASD profile (Company and Rostella 2026).

We appreciate this point and have added Company and Rostella (2026) to acknowledge the hypothesis that autonomy-centered accounts may represent a rhetorical repositioning of PDA as an autism profile. We also clarified our own position. The autonomy-centered formulation used in developing the DAAQ was not derived from an established empirical literature, which remains limited, but was developed as a hypothesis-generating framework informed by lived-experience accounts, practitioner observation, Self-Determination Theory, and preliminary stakeholder input. We agree that reframing a descriptive behavioral profile as a “drive for autonomy” moves toward an explanatory formulation and therefore requires direct empirical testing. The present study evaluates the DAAQ’s factor structure and preliminary psychometric properties; it does not establish perceived autonomy threat as the cause of demand avoidance or determine whether PDA is an autism profile.

3) Most predictions on PDA populations are based on Newson’s descriptions, such as (Gillberg 2014). This needs to be explicitly stated and considered when justifying the tool’s underpinning model. I apologise, if I missed this, but the similarities, and differences of the tool’s underpinning model, to Newson’s descriptions need to be mentioned for ease of reference.

Thank you for this helpful comment. We agree that Newson’s original clinical description has served as the foundation for much of the subsequent empirical and theoretical work on PDA. We have revised the Introduction to make this lineage more explicit.

4) Company and Rostella (2026) critique of the EDA-Q should be included in the literature review.

This review publication is now cited in several places in the manuscript.

5) The shift using a formulation, instead of a descriptive diagnosis for PDA needs to be mentioned. See the Kieran case study as example. Phil Christie, 5≥ years ago (e.g., see their talks in 2016-2019), in their various conference talks, (accurately) mentioned how psychiatric categories are based on reliability over validity, and proceeded onto reasons for using a diagnosis in research and practice settings. The hypothesis the change to a PDA formulation, from a diagnosis is due to a rhetorical repositioning of PDA as an ASD profile (Company and Rostella 2026). This matters as using a heightened drive for autonomy and equality, is used as an explanation for a person’s behaviours, instead of describing them. Changing PDA from a descriptive category to an explanatory one is MASSIVE change in PDA’s ontology, when there is insufficient evidence to warrant it. This topic needs to be discussed, even if manuscript disagrees with my views on the topic.

The Introduction now explicitly describes the autonomy-centered account as a provisional, hypothesis-generating formulation, not an established explanation, and acknowledges the limited empirical literature regarding PDA mechanisms. The limitations further recognize that moving from a descriptive behavioral profile to an autonomy-centered explanation represents a rhetorical and conceptual reframing that requires empirical evaluation, with Company and Rostella (2026) cited in support of this point. We also clarify that the DAAQ assesses behavioral expression and functional impact rather than the reasons or mechanisms underlying demand avoidance.

6) I appreciate changing the tool’s name. I am struggling to see how the current name is accurate, or helpful. This logic is suitable A+B≠A. I say this because it is called demand avoidance and autonomy questionnaire. Yet, the heightened drive for autonomy and equality is meant to be model underpinning the tool, how it can it be both (i.e., demand-avoidance itself is broader than a heightened drive for autonomy, or a heightened drive for equality. Furthermore, a heightened drive for autonomy, is only meant to be part of the causes of PDA’s demand-avoidance, according to the tool’s underpinning model)? I am not entirely surely that the model, is a model for heightened drive for autonomy and equality. The manuscript describes a drive for autonomy more than a heightened drive for equality. How well is a heightened drive for equality captured in the tool’s items? If the tool significantly assesses for a heightened drive for equality, then the tool’s name should be changed to reflect this. If the tool does not, then conceptual model underlying the tool should be updated to reflect it is not sufficiently capturing a heightened drive for equality, or be this should be noted in the limitations.

Thank you for this comment. We agree that the prior wording may have blurred the distinction between demand avoidance as the behavioral phenomenon being measured, autonomy as the primary theoretical lens guiding item development, and equality/equalizing behavior as a related but not independently scored construct. We have retained the name Demand Avoidance and Autonomy Questionnaire because it reflects the measure’s focus on observable demand-avoidance-related behaviors and an autonomy-centered conceptual framework. However, we revised the manuscript to clarify that the DAAQ does not assess all possible causes of demand avoidance and does not provide a distinct measure of heightened drive for equality. We also softened language implying that autonomy and equality were measured equivalently and added a limitation noting that equalizing behavior is represented only within the broader Behavioral Expression domain. We also now clarify the relationship among demand avoidance, autonomy, and equality.

7) Equality, as a concept is highly subjective. Values behind equality can rapidly change, and are transient; one only has to look at debates around right to vote, and/ or slavery, for different values for equality. If a heightened drive for equality conceptually underpins this tool, then the subjective nature of equality needs to be addressed.

We agree that “equality” is a subjective and culturally embedded concept, and that the manuscript needed to avoid implying a fixed or universal definition of equality. We have revised the text to clarify that equality is not treated as a standalone construct underpinning the DAAQ, nor does the measure attempt to assess values-based beliefs about equality. Specifically, we indicate that this measure assesses perceived control, fairness, or relational balance.

8) I presented an open ended question, for potential causes of Autistics scoring higher for the tool’s 3 subscales, to see if manuscript may consider environmental and cultural factors might contribute to these results. How poorly treated Autistics are by Global North cultures, is a large factor behind the atrocious mental health issues found in the Autistic population, and suicide related problems (Autistic scholars discuss these points, arguably Monique Botha’s work is amongst most important). Most people, who are as distressed and traumatised as many Autistics are, are likely to score highly on at least 1 of the tool domains. Additionally, it seems reasonable to question, if the result is at least partly caused by how PDA is often modelled as a “*Profile of ASD*”, identified in Autistics, and researched in Autistics, that this can potentially bias the tool towards Autistics. It is worth noting the hypothesis heightened drive for autonomy is due to a rhetorical repositioning of PDA as an ASD profile (Company and Rostella 2026).

Thank you for clarifying this point. We agree that higher DAAQ scores among children with co-occurring autism may reflect multiple influences and should not be interpreted as evidence that PDA is necessarily an autism profile. We revised the Discussion to acknowledge that environmental and cultural factors, including poorly adapted contexts, unmet support needs, stigma, and trauma/minority-stress-related processes, may contribute to elevated scores among autistic children. We also note that because PDA has often been conceptualized and studied in relation to autism, this may have influenced caregiver recognition and item development.

9) Unless I am missing the citations in the paragraph discussing the study’s hyperactivity and conduct issues results, they need to be situation in broader literature on the topic.

Thank you for noting this. We revised the Discussion to situate the observed associations between the DAAQ Behavioral Expression factor and SDQ Hyperactivity and Conduct Problems within the broader literature.

10) I apologise, if I missed it. May the words “*prejudice*”, and “*stigma*” be used in the manuscript? Even it they are not used to discuss potential risks of attributing PDA to an individual, some have argued a heightened drive for autonomy, is less stigmatising than using “*Pathological Demand Avoidance*” term.

Thank you for this suggestion. We agree that the language used to describe PDA/demand avoidance has implications for stigma and potential prejudice and have added this to the Introduction.

11) One of the most replicated results in PDA studies, is how behaviours assessed for by PDA tools decrease as a CYP grows into adulthood. How does this study’s results compare to those results?

Thank you. Given that the current study was cross-sectional and limited to parent reports for children/adolescents ages 4–17 (i.e., it was not designed to evaluate developmental trajectories or compare child/adolescent scores with adult presentations), we have added this as an area of future research.

12) Thank you mentioning the Attachment disorder hypothesis.

Thank you for this feedback.

13) Sample size is an indication of the passion for the PDA out there. Also reflects many other PDA studies.

Thank you for this observation. We agree.

14) L187-189: It is obvious, that researchers are going to continue to hold PDA to typical standards, which are there to protect people (Company and Rostella 2026). An alternative outlook to families feeling unseen etc, is to point to those exposing the vulnerable persons to PDA’s controversies, by prematurely reifying PDA. This is a factor why Green et al (2018), suggest utilising features from co-occurring difficulties to model PDA.

We revised the introduction to address these concerns.

15) L191. “*broader autism spectrum presentations,*”. This is a contested opinion. Yes, many view PDA to be a “profile of ASD”, but this could be due to a hypothesised rhetorical repositioning of PDA as an ASD profile. At least before 2015, PDA’s literature tended to explicitly note which features make it problematic features contradict autism understandings. That phrasing needs to be changed.

We revised this wording to be more neutral and to avoid implying that PDA is established as a subtype or profile of autism.

16) L214-218. This is conjecture. As pointed out in the first review. There are good grounds to question in PDA’s demand-avoidance is intrinsically to “*everyday*” demands, such as those associated with toileting etc. Again good reasons to question, if avoidance to demands, like hygiene matters should be associated with PDA. It is possible to gain statistics of use of EDA-Q. The manuscript needs to provide the numbers/ percentages of studies utilising EDA-Q, in Kildahl et al (2021), Company and Rostella (2026), and Haire et al (2024) respective reviews. It might also be worth noting that not all datasets including the EDA-Q have been analysed; from my understanding Help4Psychology have not had their EDA-Q data analysed for potential correlations with any tools they used as part of their assessments, such as the Short Sensory Profile. It might be worth contacting Help4Psychology, if this is still the case, and possibly comment on the topic in the manuscript.

We have revised the text to clarify that the DAAQ assesses functional impact on basic-needs because these domains are frequently reported by families and were part of our stakeholder-informed item-development process, not because we assume that avoidance of eating, sleeping, toileting, or hygiene is uniquely or necessarily specific to PDA. We also removed/softened language suggesting “limited uptake” of the EDA-Q/EDA-8 to avoid making a claim that would require a systematic accounting of measure use across reviews. We did not add analyses of external datasets, as this was beyond the scope of the present measure-development study.

17) L265. Notes purpose of the study was for the tool to capture PDA specific features. Considering the literature generally notes PDA lacks any specific features; this objective needs to be explicitly addressed in the literature review. It is worth stating PDA does not need to have any specific features for it to be included in the psychiatric manuals, as most categories and characteristics operate as continua, and overlapping each other. Most categories in the DSM lack specific causal mechanisms.

Thank you for this point. We agree that the term “PDA-specific” could imply that the literature has established features unique to PDA, which was not our intent. We revised the manuscript to use “PDA-related” throughout.

18) L292. “*An initial pool of 67 questions was designed to evaluate the key*”, implies those 67 items describe PDA, which as the manuscript notes, may not be the case. Please could it be changed to something like “*An initial pool of 67 questions was designed to evaluate the hypothesised key*”.

We have made this change as requested.

Reviewer #2:

The revised version of the study is methodologically sound, and I have no major reservations regarding the overall methodological rigour. I would like to raise two observations that I believe warrant further explanation or discussion:

1. Sample composition skew towards female parents. The majority of participants (681) identified as female parents, while the remaining participants represented other categories. Could the authors comment on whether this imbalance may have introduced any confounding influence on the results, and whether this limitation is acknowledged in the manuscript?

Thank you for raising this point. We have revised the Discussion to explicitly acknowledge this limitation and to note that DAAQ scores should be interpreted relative to the predominantly female caregiver calibration sample. We also added language recommending future validation studies with more diverse caregiver samples and multi-informant designs.

2. Factor loading magnitudes in the EFA. Several of the factor loadings in the EFA solution are close to the conventional minimum threshold (≥ .33), rather than reflecting a higher level of loading strength. I would encourage the authors to briefly discuss this in the text — for example, by clarifying the cutoff criterion used, noting any items with weaker or cross-loadings, and commenting on the implications this may have for the robustness of the factor structure.

We now clarify the cutoff criterion used (≥ .30); this criterion is consistent with the early-stage measure-development context and our goal of preserving conceptually important content for subsequent evaluation. We also now explicitly note that several items had relatively modest loadings and that one item showed cross-loading. Importantly, EFA was not used as the sole basis for final item retention. We also added language to the Discussion acknowledging that modest EFA loadings and cross-loadings suggest that some PDA-related behaviors may be heterogeneous and that future replication in independent, more diverse samples is needed.
