# Supplementary material for: The Demand Avoidance and Autonomy Questionnaire (DAAQ): Development of a new measure of demand avoidance
Source: PLoS One. 2026 Nov 11;21(11):e0345827. doi: 10.1371/journal.pone.0345827 (PMC13754100; doi:10.1371/journal.pone.0345827)
Supplement: Supplementary file 12 [file pone.0345827.s014.docx]

**Response to Reviewers**

**Reviewer #1:**

1) The manuscript is significantly strengthened over previous iterations. I appreciate the time and effort put in to do this.

Thank you for this positive feedback.

2) I am not entirely sure the manuscript feedback was necessarily acted upon. There are a couple of points of I think the manuscript should address. I am not entirely sure that Newson’s portrayal of “ordinary” demands reflects that in the paper. Newson’s account also seems focus on the impact of the demand-avoidance and other behaviours on the caregivers, and others around the PDAer.

Thank you for raising this point. We revised the Introduction to more precisely represent Newson’s original account. Specifically, we now clarify that Newson used “ordinary demands” to encompass what she described as “normal expectations,” ordinary group demands in nursery or school, educational demands, organizational rules, and adult-directed tasks. We also acknowledge her emphasis on the impact of these behaviors on parents and teachers. Finally, we distinguish Newson’s account from our observation that what is considered an ordinary or developmentally typical demand may vary across families, educational settings, social contexts, and cultures. The manuscript now states:

*“Newson characterized PDA as ‘a distinct behavioral profile involving obsessional avoidance of the ordinary demands of life, coupled with a degree of sociability that allowed social manipulation as a primary avoidance strategy.’[3] Her account used ‘ordinary demands’ to encompass ‘normal expectations,’ ordinary group demands in nursery or school, educational demands, organizational rules, and adult-directed tasks. Newson also emphasized the impact of these behaviors on parents and teachers, describing the children as particularly difficult for them to manage and teach. However, Newson did not provide a discrete or culturally neutral operational definition of what makes a demand ‘ordinary.’ What is regarded as an ordinary or developmentally typical expectation may vary across families, educational settings, social contexts, and cultures.[10]”*

3) I think it is worth quoting this part from the first peer review.:

   “I am unsure if the construct of PDA in this manuscript is the same put forward by Newson, and later knowledge derived from Newson’s work. For instance, The study states its deliberately focused on toileting and hygiene difficulties of PDAers. I accept that this a problem for some PDAers. Toileting and hygiene issues discussed in the manuscript are sufficient that they should have been picked up key previous PDA scholarship before this. These issues should be problematic enough to be brought up by caregivers, and others around the PDAers during the clinical assessment process. The toileting and other issues should be collected as part of a comprehensive assessment for autism, or other Neurodevelopmental category, if only for excluding other explanations for an assessee’s behaviour.”

We clarified that hygiene and toileting are not established as defining or PDA-specific features. Although these domains were not prominent in Newson’s original account or systematically assessed in prior PDA measures, stakeholder input identified them as potentially important functional difficulties among some children with marked demand avoidance. We also note that they may have multiple developmental, medical, sensory, psychological, or contextual explanations and require comprehensive assessment. The manuscript now states:

*“Hygiene and toileting difficulties were not prominent features of Newson’s original account and are not systematically assessed in prior PDA instruments. Nevertheless, stakeholder input identified these as potentially important functional difficulties among some children with marked demand avoidance. They may have multiple developmental, medical, sensory, psychological, or contextual explanations and should not be assumed to be core or specific features of PDA.”*

When introducing the DAAQ, we further characterize these domains as *“potentially associated functional difficulties involving basic needs … that emerged through stakeholder input.”*

4) Kildahl et al (2021) noted that Newson’s PDA conceptualisation is a mess, with many assumptions. It was this as Newson and colleagues including any significant features they observed in their PDAers. Searching Newson et al (2003) and supplementary notes using: “clean”, “hygiene”, & “toilet”, only yielded 1 instance, relation to obsessions on “over cleanliness/slovenliness” (Newson et al 2003, 598). The same 3 search terms were used on O’Nions et (2016a) DISCO paper, and its supplementary files and produced 0 results. This result was replicated in O’Nions et al (2014a) EDA-Q development paper, and O’Nions et al (2018) qualitative examination of ASD+PDA. A 22 item semi-structured interview used to assess for PDA from before 2015, does includes mentions of urinating in public in terms embarrassing and socially shocking behaviour. Considering the tools assesses for serious features, including self-injury. If toileting issues were present, they should be included in the tool. Should toileting issues be associated with PDA? Is it reflective of PDA? Are associating toileting and hygiene issues with PDA, likely to contribute to stigma and/ or prejudice experiencing by PDAers?”

As noted above, we now clarify that existing literature has not established hygiene and toileting as core or PDA-specific features. Their omission from earlier measures does not preclude assessing them as potentially associated functional difficulties identified through stakeholder input. We also emphasize that these difficulties may have multiple explanations, require contextual interpretation, and should not be used diagnostically. We agree that attributing intimate functional difficulties to PDA without appropriate context could contribute to stigma; accordingly, we emphasize contextual and non-diagnostic interpretation.

5) Even, if this how “ordinary” demands are accurately portrayed in the manuscript, others would view avoidance of “ordinary” demands to be avoidance typical Global North demands, such as in the Keiran case study from Soppitt (2021), or as argued by Critical Autism Studies scholars. In particular Allison Moore argues a person is more likely to be attributed with PDA, when the contravene cultural norms associated with autism, childhood, and female gender. This is reflected in how some have suggested PDA might be a female presentation of autism (Eaton 2018; Gould and Ashton-Smith 2011). Furthermore, there is wide variation in how PDA is identified in research and clinical settings, with some “PDA Profile of ASD” advocates claiming PDA is over identified, e.g., PDA Society. There is no consensus that “ordinary” demands as described in PDA, is what is meant as representative of PDA. Newson meant “ordinary” demands as described in the manuscript, the tool should not need to be developed, as those features would already be assessed for previous PDA tools. The manuscript needs to address how broader and diverse interprets what “ordinary” demands means.

We agree that “ordinary demands” is neither standardized nor culturally neutral, and we revised the manuscript to acknowledge variation across families, developmental and educational contexts, and cultures. We also clarified in the study-purpose paragraph that the DAAQ does not assume that such demands are objectively ordinary or benign. However, we respectfully disagree that overlap with Newson’s terminology removes the need for a new measure. The DAAQ expands stakeholder-informed content coverage and applies contemporary psychometric methods not used in developing earlier measures.

6) The underlying model for the tool needs to address how demand-avoidance is a transactional process, and that it should it be possible for a person to develop PDA after early infancy, such as being distressed and/ or traumatised into expressed demand-avoidance behaviours for sufficient period of time, that these behaviours generalise (Kildahl et al 2021). Furthermore, the model needs to account for one of most replicated results in PDA studies in CYP, that PDA behaviours tend to significantly decrease a CYP as the CYP approaches adulthood (this rate is tends to substantially higher than seen in autism, and in some studies exceeding the rates observed in ADHD). Stuart et al (2020) hypotheses this apparent feature of PDA is due to PDAers developing better coping mechanisms. This replicated feature raises reasonable doubts that PDA is intrinsically pervasive, or developmental in nature. This feature needs to be accounted for in the manuscript’s model underlying the tool.

We agree that demand avoidance may be transactional and context-dependent, and we revised the conceptual model accordingly. The manuscript now states:

*“This model is intended to generate testable hypotheses about one possible pathway to demand avoidance, not to imply that autonomy threat is its sole cause or that these processes are unique to PDA. Demand avoidance may also be transactional and context-dependent, emerging through interactions among the individual, the nature and delivery of demands, prior experiences, relationships, and environmental responses. Other neurodevelopmental and clinical presentations (e.g., autism, attention deficit hyperactivity disorder, obsessive-compulsive disorder, bipolar disorder, oppositional defiant disorder, and trauma-related conditions) may also involve a strong drive for autonomy or demand avoidance arising through sensory, cognitive, emotional, interpersonal, or contextual pathways. The present cross-sectional study cannot determine when demand avoidance develops, whether it generalizes over time, or whether it represents a stable developmental trait.”*

Although sustained distress or trauma may be one pathway, current evidence does not support adopting a single causal model. We also expanded the Limitations:

*“Longitudinal research is needed to examine whether and how demand-avoidance behaviors change as children approach adulthood. Although some prior work has reported decreases for some individuals, Newson’s follow-up sample showed heterogeneous trajectories, with avoidance decreasing in some participants, remaining stable in others, and increasing in a smaller number[3, 33]. The present cross-sectional study cannot establish that the measured behaviors are developmentally stable or intrinsically pervasive.”*

7) L328. “Interested parents of children with a suspected PDA profile”, the change in wording to add “Profile” might be pertinent. It is a choice to frame PDA as a “Profile”, it suggests a level of preconceived notions over what PDA could be and how it is modelled. This is addressed as a potential limitation.

We agree that “suspected PDA profile” reflects a particular framing. This terminology was used in recruitment and by the participating community; it was not intended to establish PDA as a validated profile, autism subtype, or diagnosis. We clarified this in the Methods:

*“The term ‘suspected PDA profile’ reflected the terminology used in recruitment and by the community from which the sample was drawn. Its use should not be interpreted as establishing PDA as a validated diagnostic profile or as resolving competing conceptualizations of demand avoidance.”*

We also acknowledge in the Limitations that this terminology may have influenced participation and caregivers’ interpretation and reporting of their children’s behavior.

Reviewer #2:

1) The authors have satisfactorily addressed the comments and concerns raised by the reviewers. The revisions have improved the clarity, methodological presentation, interpretation of the findings, and overall quality of the manuscript. The manuscript is suitable for publication in its revised form.

Thank you for this positive feedback.
