# Supplementary material for: The Demand Avoidance and Autonomy Questionnaire (DAAQ): Development of a new measure of demand avoidance
Source: PLoS One. 2026 Nov 11;21(11):e0345827. doi: 10.1371/journal.pone.0345827 (PMC13754100; doi:10.1371/journal.pone.0345827)
Supplement: Supplementary file 13 [file pone.0345827.s004.doc]

**The Demand Avoidance and Autonomy Questionnaire (DAAQ)**

**Please respond to each question or statement by marking one box per row.**

**“Child” refers to your child with demand avoidance.**

**Behavioral Expression**

|  |  | **Never** | **Rarely** | **Sometimes** | **Usually** | **Always** |
| --- | --- | --- | --- | --- | --- | --- |
| DAAQ_BE01 | When playing games, my child becomes distressed (dysregulated/agitated) if they are not winning. | 🞎  1 | 🞎  2 | 🞎  3 | 🞎  4 | 🞎  5 |
|  |  |  |  |  |  |  |
| DAAQ_BE02 | My child has difficulty with transitions to a preferred activity. | 🞎  1 | 🞎  2 | 🞎  3 | 🞎  4 | 🞎  5 |
|  |  |  |  |  |  |  |
| DAAQ_BE03 | If I try to encourage my child to participate in activities they dislike, my child will become distressed (dysregulated/agitated) and/or have a meltdown (panic attack/temper tantrum). | 🞎  1 | 🞎  2 | 🞎  3 | 🞎  4 | 🞎  5 |
|  |  |  |  |  |  |  |
| DAAQ_BE04 | When my child is in a situation they find stressful, my child behaves inappropriately (laughs or says something inappropriate, removes their clothes, makes inappropriate sounds or noises, uses nonsense words, repeats words). | 🞎  1 | 🞎  2 | 🞎  3 | 🞎  4 | 🞎  5 |
|  |  |  |  |  |  |  |
| DAAQ_BE05 | My child becomes distressed (dysregulated/agitated) when they are told that they have to stop doing a preferred activity (e.g., screen time). | 🞎  1 | 🞎  2 | 🞎  3 | 🞎  4 | 🞎  4 |
|  |  |  |  |  |  |  |
| DAAQ_BE06 | My child will refuse help, even after they have asked for it. | 🞎  1 | 🞎  2 | 🞎  3 | 🞎  4 | 🞎  5 |
|  |  |  |  |  |  |  |
| DAAQ_BE07 | My child destroys their own things. | 🞎  1 | 🞎  2 | 🞎  3 | 🞎  4 | 🞎  5 |

|  |  | **Never** | **Rarely** | **Sometimes** | **Usually** | **Always** |
| --- | --- | --- | --- | --- | --- | --- |
| DAAQ_BE08 | When my child is told to do something, they will say, “I can't”, “I don't feel well”, ignore the request, act as if they do not understand (change the topic, pretend not to hear, start doing something unrelated), pretend to be an animal, or leave the room. | 🞎  1 | 🞎  2 | 🞎  3 | 🞎  4 | 🞎  5 |
|  |  |  |  |  |  |  |
| DAAQ_BE09 | When my child is excited about something (e.g., a holiday), they become distressed (dysregulated/agitated). | 🞎  1 | 🞎  2 | 🞎  3 | 🞎  4 | 🞎  5 |
|  |  |  |  |  |  |  |
| DAAQ_BE10 | My child will show excitement about something only to refuse when it is time to participate (leave the house, go into building/event). | 🞎  1 | 🞎  2 | 🞎  3 | 🞎  4 | 🞎  5 |
|  |  |  |  |  |  |  |
| DAAQ_BE11 | My child will respond to being told “No”, “Stop” or “Don't” by doing the opposite, even if it is dangerous (runs towards the street, touches a hot stove, climbs a high ledge). | 🞎  1 | 🞎  2 | 🞎  3 | 🞎  4 | 🞎  5 |
|  |  |  |  |  |  |  |
| DAAQ_BE12 | When my child does not get their way, they will respond by knocking something down, shouting, calling names, needing to have the last word, or destroying something. | 🞎  1 | 🞎  2 | 🞎  3 | 🞎  4 | 🞎  5 |
|  |  |  |  |  |  |  |
| DAAQ_BE13 | When I ask my child a direct question, they respond with a growl or hissing sound, an "I don't know", "stop talking", silence, or a silly word like “poop”. | 🞎  1 | 🞎  2 | 🞎  3 | 🞎  4 | 🞎  5 |
|  |  |  |  |  |  |  |
| DAAQ_BE14 | When I tell my child to do something, they respond with a growl or hissing sound, an “I don't know”, “stop talking”, silence, or a silly word like “poop”. | 🞎  1 | 🞎  2 | 🞎  3 | 🞎  4 | 🞎  5 |

|  |  | **Never** | **Rarely** | **Sometimes** | **Usually** | **Always** |
| --- | --- | --- | --- | --- | --- | --- |
| DAAQ_BE15 | Unless my child is participating in a preferred activity (e.g., on a screen or a special interest), they become distressed (dysregulated/agitated), or have a meltdown (panic attack/temper tantrum) when I try to talk to another person. | 🞎  1 | 🞎  2 | 🞎  3 | 🞎  4 | 🞎  5 |
|  |  |  |  |  |  |  |
| DAAQ_BE16 | Unless my child is participating in a preferred activity (e.g., on a screen, or a special interest), or with someone they trust, he/she will become distressed (dysregulated/agitated) or have a meltdown (panic attack/temper tantrum) if I leave. | 🞎  1 | 🞎  2 | 🞎  3 | 🞎  4 | 🞎  5 |
|  |  |  |  |  |  |  |
| DAAQ_BE17 | My child wants me to stay nearby, but does not want me to touch or interact with them. | 🞎  1 | 🞎  2 | 🞎  3 | 🞎  4 | 🞎  5 |

|  |  | **Strongly disagree** | **Disagree** | **Somewhat disagree** | **Somewhat agree** | **Agree** | **Strongly agree** |
| --- | --- | --- | --- | --- | --- | --- | --- |
| DAAQ_BE18 | My child likes to be physically "above" people (e.g., likes to sit or stand above other people, stand on chairs or the couch, climb onto windowsills, sit on the back of the couch). | 🞎  1 | 🞎  2 | 🞎  3 | 🞎  4 | 🞎  5 | 🞎  6 |
|  |  |  |  |  |  |  |  |
| DAAQ_BE19 | My child breaks/ignores rules, but gets upset if others do not follow them. | 🞎  1 | 🞎  2 | 🞎  3 | 🞎  4 | 🞎  5 | 🞎  6 |
|  |  |  |  |  |  |  |  |
| DAAQ_BE20 | My child needs constant attention or they will become distressed (dysregulated/agitated) or have a meltdown (panic attack/temper tantrum). | 🞎  1 | 🞎  2 | 🞎  3 | 🞎  4 | 🞎  5 | 🞎  6 |

**Basic Needs**

|  |  | **Never** | **Rarely** | **Sometimes** | **Usually** | **Always** |
| --- | --- | --- | --- | --- | --- | --- |
| DAAQ_BN01 | My child resists sleeping. | 🞎  1 | 🞎  2 | 🞎  3 | 🞎  4 | 🞎  5 |
|  |  |  |  |  |  |  |
| DAAQ_BN02 | My child has an irregular sleep cycle (e.g., they go to sleep at different times and wake up at different times each night/day). | 🞎  1 | 🞎  2 | 🞎  3 | 🞎  4 | 🞎  5 |
|  |  |  |  |  |  |  |
| DAAQ_BN03 | My child resists bathing or showering. | 🞎  1 | 🞎  2 | 🞎  3 | 🞎  4 | 🞎  5 |
|  |  |  |  |  |  |  |
| DAAQ_BN04 | My child resists brushing their teeth. | 🞎  1 | 🞎  2 | 🞎  3 | 🞎  4 | 🞎  5 |

|  |  | **Strongly disagree** | **Disagree** | **Somewhat disagree** | **Somewhat agree** | **Agree** | **Strongly agree** |
| --- | --- | --- | --- | --- | --- | --- | --- |
| DAAQ_BN05 | My child needs to be in control of when, where and/or what they eat. | 🞎  1 | 🞎  2 | 🞎  3 | 🞎  4 | 🞎  5 | 🞎  6 |
|  |  |  |  |  |  |  |  |
| DAAQ_BN06 | My child will only eat preferred foods. | 🞎  1 | 🞎  2 | 🞎  3 | 🞎  4 | 🞎  5 | 🞎  6 |
|  |  |  |  |  |  |  |  |
| DAAQ_BN07 | My child needs to be in control of when and/or where they sleep. | 🞎  1 | 🞎  2 | 🞎  3 | 🞎  4 | 🞎  5 | 🞎  6 |
|  |  |  |  |  |  |  |  |
| DAAQ_BN08 | My child needs to be in control of when and/or where and/or how they bathe or shower. | 🞎  1 | 🞎  2 | 🞎  3 | 🞎  4 | 🞎  5 | 🞎  6 |
|  |  |  |  |  |  |  |  |
| DAAQ_BN09 | My child needs to be in control of when and/or where and/or how they brush their teeth. | 🞎  1 | 🞎  2 | 🞎  3 | 🞎  4 | 🞎  5 | 🞎  6 |
|  |  |  |  |  |  |  |  |
| DAAQ_BN10 | My child needs to be in control of when and/or where and/or how they brush their hair. | 🞎  1 | 🞎  2 | 🞎  3 | 🞎  4 | 🞎  5 | 🞎  6 |

**Toileting**

|  |  | **Never** | **Rarely** | **Sometimes** | **Usually** | **Always** |
| --- | --- | --- | --- | --- | --- | --- |
| DAAQ_TL01 | My child has toileting accidents. | 🞎  1 | 🞎  2 | 🞎  3 | 🞎  4 | 🞎  5 |
|  |  |  |  |  |  |  |
| DAAQ_TL02 | When my child becomes distressed (dysregulated/agitated), their ability to toilet independently decreases (wipe themselves, use the toilet alone). | 🞎  1 | 🞎  2 | 🞎  3 | 🞎  4 | 🞎  5 |
|  |  |  |  |  |  |  |
| DAAQ_TL03 | My child withholds his/her urine (pee) or stool (poop). | 🞎  1 | 🞎  2 | 🞎  3 | 🞎  4 | 🞎  5 |

|  |  | **Strongly disagree** | **Disagree** | **Somewhat disagree** | **Somewhat agree** | **Agree** | **Strongly agree** |
| --- | --- | --- | --- | --- | --- | --- | --- |
| DAAQ_TL04 | My child resists going to the bathroom. | 🞎  1 | 🞎  2 | 🞎  3 | 🞎  4 | 🞎  5 | 🞎  6 |
|  |  |  |  |  |  |  |  |
| DAAQ_TL05 | My child needs to be in control of when and/or where they go to the bathroom (or have diaper changes). | 🞎  1 | 🞎  2 | 🞎  3 | 🞎  4 | 🞎  5 | 🞎  6 |
|  |  |  |  |  |  |  |  |
| DAAQ_TL06 | My child has lost skills related to toileting (needing to use a diaper again, toileting outside or in a particular location like the bathtub, or refusing all together). | 🞎  1 | 🞎  2 | 🞎  3 | 🞎  4 | 🞎  5 | 🞎  6 |

**Instructions for Scoring**

This Demand Avoidance and Autonomy Questionnaire (DAAQ) is 36-item questionnaire comprised of three factors, each represented by an individual item bank with their own scoring table.

**Behavioral Expression:** Items DAAQ_BE01 to DAAQ_BE20

**Basic Needs:** Items DAAQ_BN01 to DAAQ_BN10

**Toileting:** Items DAAQ_TL01 to DAAQ_TL06

Each response option is assigned a value (e.g., 1=Never for DAAQ_BE01). For each factor, you need to calculate a total summed raw score for each respondent. To find the total raw score for each factor, sum the values of the response to each question in that factor. Use the conversion tables below to translate the total raw score into an IRT-based T-score for each factor.

**Important:** Please note that both “Usually” and “Always” responses for item BE05 in Behavioral Expression is scored as a “4.”

**Meaning of T-Scores**

T-score distributions rescale raw scores into standardized scores with a mean of 50 and a standard deviation (SD) of 10. Thus, a T-score of 60 is one SD above the average of the referenced population (parent proxy of neurodivergent children with a suspected or confirmed pathological demand avoidance [PDA] profile).

**Scoring Tables**

**Behavioral Expression**

A higher Behavioral Expression T-score represents more negative behavioral expression, so a T-score of 60 is one SD worse than the averaged reference population (parent proxy of neurodivergent children with a suspected or confirmed PDA profile).

| DAAQ – Behavioral Expression  *Conversion Table* | | |
| --- | --- | --- |
| Raw Score | T- Score | SE |
| 20 | 11.5 | 1.4 |
| 21 | 11.6 | 1.5 |
| 22 | 11.8 | 1.6 |
| 23 | 12.0 | 1.7 |
| 24 | 12.3 | 1.9 |
| 25 | 12.6 | 2.1 |
| 26 | 12.9 | 2.3 |
| 27 | 13.3 | 2.4 |
| 28 | 13.8 | 2.6 |
| 29 | 14.4 | 2.8 |
| 30 | 15.1 | 3.0 |
| 31 | 15.8 | 3.2 |
| 32 | 16.6 | 3.3 |
| 33 | 17.4 | 3.4 |
| 34 | 18.3 | 3.5 |
| 35 | 19.2 | 3.5 |
| 36 | 20.1 | 3.5 |
| 37 | 21.0 | 3.5 |
| 38 | 21.9 | 3.5 |
| 39 | 22.7 | 3.5 |
| 40 | 23.6 | 3.5 |
| 41 | 24.4 | 3.5 |
| 42 | 25.3 | 3.5 |
| 43 | 26.1 | 3.5 |
| 44 | 26.9 | 3.5 |
| 45 | 27.7 | 3.4 |
| 46 | 28.5 | 3.4 |
| 47 | 29.3 | 3.4 |
| 48 | 30.1 | 3.4 |
| 49 | 30.9 | 3.4 |
| 50 | 31.7 | 3.4 |
| 51 | 32.5 | 3.4 |
| 52 | 33.3 | 3.4 |
| 53 | 34.1 | 3.4 |
| 54 | 34.8 | 3.4 |
| 55 | 35.6 | 3.5 |
| 56 | 36.4 | 3.5 |
| 57 | 37.2 | 3.5 |
| 58 | 38.0 | 3.5 |
| 59 | 38.8 | 3.5 |
| 60 | 39.5 | 3.5 |
| 61 | 40.3 | 3.5 |
| 62 | 41.1 | 3.5 |
| 63 | 41.9 | 3.5 |
| 64 | 42.7 | 3.5 |
| 65 | 43.5 | 3.5 |
| 66 | 44.3 | 3.5 |
| 67 | 45.1 | 3.5 |
| 68 | 45.9 | 3.5 |
| 69 | 46.7 | 3.5 |
| 70 | 47.5 | 3.5 |
| 71 | 48.4 | 3.5 |
| 72 | 49.2 | 3.5 |
| 73 | 50.0 | 3.5 |
| 74 | 50.9 | 3.5 |
| 75 | 51.7 | 3.5 |
| 76 | 52.6 | 3.5 |
| 77 | 53.4 | 3.5 |
| 78 | 54.3 | 3.5 |
| 79 | 55.2 | 3.5 |
| 80 | 56.1 | 3.5 |
| 81 | 57.0 | 3.5 |
| 82 | 57.9 | 3.5 |
| 83 | 58.8 | 3.6 |
| 84 | 59.8 | 3.6 |
| 85 | 60.8 | 3.6 |
| 86 | 61.7 | 3.6 |
| 87 | 62.7 | 3.6 |
| 88 | 63.8 | 3.6 |
| 89 | 64.8 | 3.7 |
| 90 | 65.9 | 3.7 |
| 91 | 67.0 | 3.8 |
| 92 | 68.2 | 3.8 |
| 93 | 69.4 | 3.8 |
| 94 | 70.7 | 3.9 |
| 95 | 72.0 | 4.0 |
| 96 | 73.4 | 4.1 |
| 97 | 74.9 | 4.1 |
| 98 | 76.5 | 4.2 |
| 99 | 78.2 | 4.3 |
| 100 | 79.9 | 4.3 |
| 101 | 81.7 | 4.2 |
| 102 | 83.4 | 3.9 |

**Basic Needs**

A higher Basic Needs T-score represents more problems with basic needs, so a T-score of 60 is one SD worse than the averaged reference population (parent proxy of neurodivergent children with a suspected or confirmed PDA profile).

| DAAQ – Basic Needs  *Conversion Table* | | |
| --- | --- | --- |
| Raw Score | T- Score | SE |
| 10 | 14.8 | 3.1 |
| 11 | 15.8 | 3.4 |
| 12 | 17.0 | 3.7 |
| 13 | 18.2 | 3.8 |
| 14 | 19.4 | 3.9 |
| 15 | 20.6 | 3.9 |
| 16 | 21.8 | 3.9 |
| 17 | 22.9 | 3.9 |
| 18 | 24.0 | 3.8 |
| 19 | 25.1 | 3.8 |
| 20 | 26.1 | 3.7 |
| 21 | 27.2 | 3.7 |
| 22 | 28.1 | 3.6 |
| 23 | 29.1 | 3.6 |
| 24 | 30.1 | 3.6 |
| 25 | 31.0 | 3.6 |
| 26 | 31.9 | 3.6 |
| 27 | 32.9 | 3.6 |
| 28 | 33.8 | 3.6 |
| 29 | 34.7 | 3.6 |
| 30 | 35.6 | 3.6 |
| 31 | 36.6 | 3.6 |
| 32 | 37.5 | 3.6 |
| 33 | 38.4 | 3.7 |
| 34 | 39.4 | 3.7 |
| 35 | 40.3 | 3.7 |
| 36 | 41.3 | 3.7 |
| 37 | 42.3 | 3.7 |
| 38 | 43.3 | 3.7 |
| 39 | 44.3 | 3.8 |
| 40 | 45.3 | 3.8 |
| 41 | 46.4 | 3.8 |
| 42 | 47.5 | 3.9 |
| 43 | 48.7 | 3.9 |
| 44 | 49.9 | 4.0 |
| 45 | 51.1 | 4.0 |
| 46 | 52.4 | 4.1 |
| 47 | 53.8 | 4.2 |
| 48 | 55.2 | 4.3 |
| 49 | 56.7 | 4.5 |
| 50 | 58.3 | 4.6 |
| 51 | 60.0 | 4.8 |
| 52 | 61.8 | 5.0 |
| 53 | 63.7 | 5.2 |
| 54 | 65.9 | 5.4 |
| 55 | 68.4 | 5.6 |
| 56 | 71.7 | 6.0 |

**Toileting**

A higher Toileting T-score represents more problems with toileting, so a T-score of 60 is one SD worse than the averaged reference population (parent proxy of neurodivergent children with a suspected or confirmed PDA profile).

| DAAQ – Toileting  *Conversion Table* | | |
| --- | --- | --- |
| Raw Score | T- Score | SE |
| 6 | 30.3 | 5.3 |
| 7 | 34.6 | 4.4 |
| 8 | 37.4 | 4.0 |
| 9 | 38.8 | 4.1 |
| 10 | 40.3 | 3.9 |
| 11 | 41.9 | 3.7 |
| 12 | 43.3 | 3.5 |
| 13 | 44.7 | 3.4 |
| 14 | 45.9 | 3.3 |
| 15 | 47.1 | 3.3 |
| 16 | 48.3 | 3.3 |
| 17 | 49.5 | 3.2 |
| 18 | 50.6 | 3.2 |
| 19 | 51.7 | 3.2 |
| 20 | 52.8 | 3.2 |
| 21 | 54.0 | 3.3 |
| 22 | 55.1 | 3.3 |
| 23 | 56.3 | 3.3 |
| 24 | 57.5 | 3.3 |
| 25 | 58.8 | 3.4 |
| 26 | 60.1 | 3.4 |
| 27 | 61.5 | 3.4 |
| 28 | 63.0 | 3.5 |
| 29 | 64.6 | 3.6 |
| 30 | 66.4 | 3.7 |
| 31 | 68.6 | 4.0 |
| 32 | 71.0 | 4.3 |
| 33 | 74.6 | 4.9 |
