# Supplementary material for: The Demand Avoidance and Autonomy Questionnaire (DAAQ): Development of a new measure of demand avoidance
Source: PLoS One. 2026 Nov 11;21(11):e0345827. doi: 10.1371/journal.pone.0345827 (PMC13754100; doi:10.1371/journal.pone.0345827)
Supplement: Peer review history

# Peer review: Decision Letter 0

**DOI:** 10.1371/journal.pone.0345827.r001

**Authors:** Steve Zimmerman

3 Apr 2026

Dear Dr. Carlozzi,

Thank you for submitting your manuscript to PLOS ONE. After careful consideration, we feel that it has merit but does not fully meet PLOS ONE’s publication criteria as it currently stands. Therefore, we invite you to submit a revised version of the manuscript that addresses the points raised during the review process.

-->Please find the reviewer's comments attached. Could you please revise the manuscript to carefully address the concerns raised?

Please submit your revised manuscript by May 17 2026 11:59PM. If you will need more time than this to complete your revisions, please reply to this message or contact the journal office at plosone@plos.org. When you're ready to submit your revision, log on to https://www.editorialmanager.com/pone/ and select the 'Submissions Needing Revision' folder to locate your manuscript file.

- A letter that responds to each point raised by the academic editor and reviewer(s). You should upload this letter as a separate file labeled 'Response to Reviewers'.
- A marked-up copy of your manuscript that highlights changes made to the original version. You should upload this as a separate file labeled 'Revised Manuscript with Track Changes'.
- An unmarked version of your revised paper without tracked changes. You should upload this as a separate file labeled 'Manuscript'.

If you would like to make changes to your financial disclosure, please include your updated statement in your cover letter. Guidelines for resubmitting your figure files are available below the reviewer comments at the end of this letter.

If applicable, we recommend that you deposit your laboratory protocols in protocols.io to enhance the reproducibility of your results. Protocols.io assigns your protocol its own identifier (DOI) so that it can be cited independently in the future. For instructions see: https://journals.plos.org/plosone/s/submission-guidelines#loc-laboratory-protocols. Additionally, PLOS ONE offers an option for publishing peer-reviewed Lab Protocol articles, which describe protocols hosted on protocols.io. Read more information on sharing protocols at https://plos.org/protocols?utm_medium=editorial-email&utm_source=authorletters&utm_campaign=protocols.

As the corresponding author, your ORCID iD is verified in the submission system and will appear in the published article. PLOS supports the use of ORCID, and we encourage all coauthors to register for an ORCID iD and use it as well. Please encourage your coauthors to verify their ORCID iD within the submission system before final acceptance, as unverified ORCID iDs will not appear in the published article. *Only*  the individual author can complete the verification step; PLOS staff *cannot*  verify ORCID iDs on behalf of authors.

We look forward to receiving your revised manuscript.

Kind regards,

Steve Zimmerman, PhD

Senior Editor, PLOS One

Journal Requirements:

When submitting your revision, we need you to address these additional requirements.

1. Please ensure that your manuscript meets PLOS ONE's style requirements, including those for file naming. The PLOS ONE style templates can be found at

https://journals.plos.org/plosone/s/file?id=wjVg/PLOSOne_formatting_sample_main_body.pdf and

https://journals.plos.org/plosone/s/file?id=ba62/PLOSOne_formatting_sample_title_authors_affiliations.pdf

2. In the online submission form you indicate that your data is not available for proprietary reasons and have provided a contact point for accessing this data. Please note that your current contact point is a co-author on this manuscript. According to our Data Policy, the contact point must not be an author on the manuscript and must be an institutional contact, ideally not an individual. Please revise your data statement to a non-author institutional point of contact, such as a data access or ethics committee, and send this to us via return email. Please also include contact information for the third party organization, and please include the full citation of where the data can be found.

3. Please amend either the title on the online submission form (via Edit Submission) or the title in the manuscript so that they are identical.

4. Please include your full ethics statement in the ‘Methods’ section of your manuscript file. In your statement, please include the full name of the IRB or ethics committee who approved or waived your study, as well as whether or not you obtained informed written or verbal consent. If consent was waived for your study, please include this information in your statement as well.

5. We notice that your supplementary tables are included in the manuscript file. Please remove them and upload them with the file type 'Supporting Information'. Please ensure that each Supporting Information file has a legend listed in the manuscript after the references list.

6. If the reviewer comments include a recommendation to cite specific previously published works, please review and evaluate these publications to determine whether they are relevant and should be cited. There is no requirement to cite these works unless the editor has indicated otherwise.

[Note: HTML markup is below. Please do not edit.]

Reviewers' comments:

Reviewer's Responses to Questions

**Comments to the Author**

1. Is the manuscript technically sound, and do the data support the conclusions?

Reviewer #1: Yes

**********

2. Has the statistical analysis been performed appropriately and rigorously?

Reviewer #1: Yes

**********

3. Have the authors made all data underlying the findings in their manuscript fully available?

The PLOS Data policy

Reviewer #1: Yes

**********

4. Is the manuscript presented in an intelligible fashion and written in standard English?

Reviewer #1: Yes

**********

Reviewer #1: (No Response)

**********

what does this mean?). If published, this will include your full peer review and any attached files.

If you choose “no”, your identity will remain anonymous but your review may still be made public.

**Do you want your identity to be public for this peer review?**  For information about this choice, including consent withdrawal, please see our Privacy Policy

Reviewer #1: **Yes:**  Richard Woods.

**********

[NOTE: If reviewer comments were submitted as an attachment file, they will be attached to this email and accessible via the submission site. Please log into your account, locate the manuscript record, and check for the action link "View Attachments". If this link does not appear, there are no attachment files.]

To ensure your figures meet our technical requirements, please review our figure guidelines: https://journals.plos.org/plosone/s/figures

You may also use PLOS’s free figure tool, NAAS, to help you prepare publication quality figures: https://journals.plos.org/plosone/s/figures#loc-tools-for-figure-preparation.

NAAS will assess whether your figures meet our technical requirements by comparing each figure against our figure specifications.

Attachment Submitted filename: Plos_One_Peer_Review..docx

Attachment Submitted filename: Elizabeth_Newson_Centre_PDA_interview_with_scoring.pdf

# Author comment: Author response to Decision Letter 1

**DOI:** 10.1371/journal.pone.0345827.r002

2 Jun 2026

Response to Reviewers

Key points.

This manuscript should be published, if it acts upon the feedback provided.

There seems to be nothing technically wrong with the manuscript in its present iteration. It is an interesting and novel study. I major concerns over the potential harm this manuscript could do, if published in its present state.

Thank you for this positive feedback.

Observations on the Manuscript.

The study was an international study, in terms of focusing in English speaking Global North, and representing the views of some PDAers. It remains to be seen if this tool represents to views of PDAers from non-English speaking countries, in particularly countries from the Global Majority, which have diverse cultural values and expectations.

We agree with this point and now include this as a study limitation.

It is unclear if tool represents the views of both non-speaking PDAers, and PDAers with co-occurring Intellectual Disability (I could not find any suitable mention on this topic, or indicators for this population of PDAers in the sample).

Thank you for this feedback. We did collect parent-reported developmental delays for the child with PDA. We have now added this information to Table 1.

There is no consensus over how to identify PDA, and so there is diverse variation in the presentations of PDA identified. Some PDA advocates claim PDA is over identified (Who is to say, which approach to PDA has any more merit than another?) Ironically, some PDAers avoid the demands “Pervasive Drive for Autonomy” community, how well are their views captured unlikely to be captured by the methods of this study. Final point, is that some PDAers, such as Damian Milton, and Richard Woods, disagree with the “Pervasive Drive for Autonomy” conceptualisation of PDA, and instead prefer other terms, such as “Rational Demand Avoidance”. This should be reflected in potential limitations of the study, and reflected in suggestions for the tool’s use.

We have now added discussion about the fact that there is no consensus definition or consistent terminology for referencing PDA to the introduction section.

I am unsure if the construct of PDA in this manuscript is the same put forward by Newson, and later knowledge derived from Newson’s work. For instance, The study states its deliberately focused on toileting and hygiene difficulties of PDAers. I accept that this a problem for some PDAers. Toileting and hygiene issues discussed in the manuscript are sufficient that they should have been picked up key previous PDA scholarship before this. These issues should be problematic enough to be brought up by caregivers, and others around the PDAers during the clinical assessment process. The toileting and other issues should be collected as part of a comprehensive assessment for autism, or other Neurodevelopmental category, if only for excluding other explanations for an assessee’s behaviour.

Thank you for requesting this clarification. Our conceptualization builds upon Newson’s work, but it is not fully aligned with it. We have added text in the introduction that highlights the ways in which our conceptualization overlaps and the ways in which our conceptualization differs from Newson’s original framework.

Kildahl et al (2021) noted that Newson’s PDA conceptualisation is a mess, with many assumptions. It was this as Newson and colleagues including any significant features they observed in their PDAers. Searching Newson et al (2003) and supplementary notes using: “clean”, “hygiene”, & “toilet”, only yielded 1 instance, relation to obsessions on “over cleanliness/slovenliness” (Newson et al 2003, 598). The same 3 search terms were used on O’Nions et (2016a) DISCO paper, and its supplementary files and produced 0 results. This result was replicated in O’Nions et al (2014a) EDA-Q development paper, and O’Nions et al (2018) qualitative examination of ASD+PDA. A 22 item semi-structured interview used to assess for PDA from before 2015, does includes mentions of urinating in public in terms embarrassing and socially shocking behaviour. Considering the tools assesses for serious features, including self-injury. If toileting issues were present, they should be included in the tool. Should toileting issues be associated with PDA? Is it reflective of PDA? Are associating toileting and hygiene issues with PDA, likely to contribute to stigma and/ or prejudice experiencing by PDAers?

This is an excellent point. You are correct in that these factors were not a part of Newson’s original framework, and this is one way in which our conceptualization differs from hers. As noted above, we now clarify how our theory compares to Newson’s and provide support for including this additional component in our framework. We are also working on a separate manuscript that further elucidates this component in our framing of PDA.

Another reason to question if how PDA is conceptualised in this manuscript is significantly similar to Newson’s, is its relationship to hyperactivity and conduct problems. Previous research indicates that PDA features are predicted by hyperactivity and conduct problems (e.g., Egan et al 2020, Green et al 2018a pp.6-8). Anna Goodson 2018’s doctoral study indicated that about a third of PDA features are correlated with Executive Functioning issues (which are typically associated with ADHD). The study should have replicated other previous results. I suspect that if the full EDA-Q was used, instead of the EDA-8, at least for the EDA-Q, the predictive relationship between hyperactivity and conduct problems, and PDA, would been replicated. There should be stronger correlations between the tool and the hyperactivity and conduct scales on Strengths and Difficulties Questionnaire (SDQ), if how PDA is conceptualised in the manuscript is significantly similar to previous conceptualisations of PDA. Please can the authors reflect upon and discuss why there is a difference between the tools correlation with hyperactivity and conduct scales, which is different to previous PDA research.

We again agree with your point, and we believe that differences in our framing of PDA may account for these discrepancies. We now raise this issue in the introduction section and note our use of the EDA-8 as a study limitation.

A third factor consider is, the wording used to describe the nature of the demand-avoidance. Newson and most later PDA research, describe the avoidance as “obsessive” in nature, and to “ordinary” demands, not “everyday” demands, such as basic needs described in the manuscript. One can argue that the difference between “ordinary” demands, and “everyday” demands is crucial, with “ordinary” demands, include things like going to work, to change activity at school, which can be perceived as a level of functioning above toileting, and hygiene. It seems reasonable to question, if PDA described in this manuscript has a higher threshold, and thus excludes more cases than other PDA conceptualisations.

As mentioned above, we have reframed the introduction and now explicitly discuss these points.

Beyond these concerns over how representative is the tool, for most PDAers, and it’s similarities to other PDA models. There is substantial concerns over the prejudice and stigma caused by attributing PDA to individuals. Particularly, notable concerns over the use of the “pathological” descriptor with PDA. Elizabeth Newson disliked the term, and others have repeated such views, like Judy Eaton said “pathological” descriptor is derogatory, and instead proposed “Extreme Anxiety” (Eaton 2018). The depth of unease with the “pathological” descriptor is represented by there being about 20 alternative names in print for PDA. The prejudice and stigma originating from language around PDA matter, for example, who would wish to provide an educational placement to child, or young person, described as having an avoidance “ordinary”/ “everyday” demands? There accounts of CYP being attributed with PDA being denied educational placements due to their PDA attributions. The issues surrounding the “pathological” descriptor need to discussed in the manuscript, and its use in the tools name needs to be justified. If it cannot be, the tool’s name should be changed.

We fully agree with these points, but we elected to use “PDA” in this manuscript to align with how it is referenced in the published literature. That said, we have revised the name of our new measure to the Demand Avoidance and Autonomy Questionnaire (DAAQ) to better reflect our conceptual framing of PDA and remove reference to pathological.

I have significant concern over the potential stigma and prejudice this tool can add to PDAers, and Autistics through the addition of the 2 new scales, and nature of the difficulties being described in its items (similar concerns are expressed elsewhere over other PDA tools, due to assessing for problematic features such as, lying, stealing, bullying, and various violent acts etc – see Woods 2024). While the manuscript notes, there is disagreement over if PDA is a “Profile of ASD”, it is often portrayed as such by many of its advocates. There are risks of associating the toileting, hygiene, and other difficulties with autism, can contribute to the stigma and prejudice associated for all Autistics. This matters as DSM-4 autism subtypes of Asperger’s Syndrome, Autistic Disorder, and Pervasive Developmental Disorder-Not Otherwise Specified (PDD-NOS), were partly excluded from the DSM-5, to reduce stigma for all Autistics (Happe 2011; Kapp & Ne’eman 2019). By creating a PDA tool to assess for basic needs such as eating, sleeping, toileting, and hygiene, one can argue that how PDA is conceptualised in this manuscript, that PDA is being modelled as a functioning category. There extant debates over the use of functioning categories in autism, with the use proposed subgroup of “Profound Autism”. The manuscript describes PDA’s difficulties as “profound”. These debates need to be directly acknowledged in the manuscript, and how they relate to how PDA is conceptualised in the manuscript should be discussed, especially relating to prejudice and stigma.

Thank you for this feedback. We appreciate the concerns raised, but we also believe that these difficulties—regardless of whether they are framed as ASD-related—do appear to present in these children. We hope that renaming our measure to be more symptom-specific rather than “syndrome”-specific partially addresses these concerns and helps mitigate potential downstream stigma. That said, whether these difficulties persist into adulthood is an empirical question; accordingly, we now note this in the Discussion in the hope that it further addresses these concerns.

It is a choice to conceptualise PDA as a “Pervasive Drive for Autonomy”, and to aim for PDA to have specific features. Generally, PDA literature acknowledges there are no specific features to PDA. DSM-5 acknowledges that most features represented by diagnostic categories described within lack specific features, and they present as continua within human population. This results in most diagnostic categories overlapping, with other categories in the DSM-5. It should be possible to conceptualise PDA as a Neurodevelopmental disorder at lower diagnostic thresholds, like ADHD, which only needs to be expressed in at least 2 settings (one in social interactions), and be consistently expressed by the age of 12. Some PDA advocates claim the EDA-Q can provide false positive results (see PDA Society’s 2022 document on assessing for PDA). Requiring PDAers to conform to “Pervasive Drive for Autonomy”, risks stripping support packages and possible sense of self understanding, from people who have been attributed with PDA that do not conform to “Pervasive Drive for Autonomy”. Some PDA advocates claim PDA is over diagnosed. It becomes a self-fulfilling prophecy, if one chooses to model PDA with a high exclusion rate. One could argue it, can be seen as an act of gatekeeping.

Although this is not a focus of the present article, one of our broader aims is to encourage further study of this symptom profile, including consideration by the DSM. DSM proposals require a clear rationale and historical context for the change, as well as discussion of potential negative consequences and arguments against the change, and an overview of significant controversies or disagreements among researchers and clinicians. In addition, for a topic to be considered, it is important to document inconsistent clinical terminology (e.g., “demand avoidant profile,” “ASD with extreme anxiety-driven avoidance,” “low tolerance for demands”) and to explain how adopting a more consensus-based term (whether PDA or another label) could standardize language and reduce diagnostic ambiguity. These considerations are central to our work as part of a broader research program aimed at clarifying core symptoms and delineating how this profile overlaps with, and/or is distinct from, autism—prerequisites for evaluating whether it is best conceptualized as a standalone diagnosis or as an autism-related profile.

I cannot support the use of this tool in assessing for PDA for the reasons outlined in the above paragraphs. considering questions over how representative it is of majority of the PDAer population. Questions of it is similar construct, as described in earlier PDA scholarship. Potential harm it can cause from extra prejudice and stigma. EDA-Q which was developed in similar circumstances to the tool in this manuscript, and is advised against using the EDA-Q in PDA assessments. Additionally, we need to be sure that knowledge derived from PDA research is applicable to all PDAers. Please can the authors reflect upon these points, and discuss it in the manuscript.

Thank you for these thoughtful comments. We have now incorporated these points into the manuscript. We reference the ongoing debate in the field and explain why we believe this new measure can help move the literature forward. Specifically, we describe the measure as one step in a longer process to evaluate how well these domains represent PDA as we currently conceptualize it, while also generating data that may identify where our conceptualization is not supported. We also note that we intentionally selected an item-banking approach because it enables the addition or removal of items and subsequent recalibration, allowing earlier versions of the measure to be directly mapped onto later versions. Given the lack of consensus regarding PDA, we position the measure as a research tool: it may yield clinically meaningful information, but it is not intended to function as a diagnostic instrument. We have clarified this in the manuscript as well.

Ironically, the paper notes a lack of use of the EDA-Q in PDA studies, and this study only uses the EDA-8. There is at least one instance of the EDA-Q being referred to as the EDA-8 on L351. It is unclear why the full EDA-Q was not used, when only the EDA-8 was. The full rationale for using the EDA-8, instead of the EDA-Q should be provided in the manuscript. Typos of the EDA-Q, should be changed to the EDA-8, and vice versa.

We now include this rationale as a study limitation and have corrected the discrepancies in the manuscript.

The manuscript should have a more in depth discussion of the EDA-Q, including the limitations observed in the PDA literature (Woods 2020, commenting on Stuart et al 2020, collated some of the critique of EDA-Q). Why not revise the EDA-Q to assess for access basic needs, such as eating, sleeping, toileting, and hygiene? Why not adapt the EDA-Q for self-assessment for under-18s, particularly as it should be less of a demand than a tool with 36 items in this tool? Please can the authors reflect upon, and discuss these points in the manuscript.

Thank you for this feedback. The EDAQ was developed using classical test theory, whereas our tool was developed using item response theory, which offers advantages in administration, adaptability, and measurement precision. We now include additional discussion of the EDAQ’s strengths and limitations, as well as the differences between these development approaches. We hope these additions—along with our expanded discussion of how our theoretical framework differs from the EDAQ—address these points.

In my view it should be interesting if the full 30 candidate items of the EDA-Q was used in this study as it includes items to assess for other reported aspects of PDA, like developmental features and 2 items indicative of social communication issues. For reference these are the 4 items removed in O’Nions et al (2014a, supplementary file):

• Has difficulty reading body language, facial expression

• Slow to process or respond to questions/ comments

• Able to take responsibility for misbehaviour

• Floppy as an infant

Please could the authors reflect upon, and discuss if future research should be conducted investigating potential relationships between the tool, and the 30 candidate items of the EDA-Q?

Thank you for this suggestion. We used the EDAQ-8 to obtain preliminary convergent validity evidence without unduly burdening participants. We agree that future work should compare EDA-Q with the DAAQ, and we now note this as a limitation and a direction for future research in the manuscript.

For purposes of the literature review, off the top of my head, these are the other extant tools used in PDA research/ assessment.

• Autism Diagnostic Observation Schedule-1 PDA items (O’Nions 2013).

• Autism Diagnostic Observation Schedule-2 Suggested Scoring (Eaton & Weaver 2020).

• Autism Spectrum Screening Questionnaire (ASSQ)-Revised Extended Version (ASSQ-REV) (Kopp & Gillberg 2011).

• Coventry Grid Interview plus Pathological Demand Avoidance (CGI-PDA) (Eaton et al 2018).

• Extreme Demand Avoidance-Questionnaire (EDA-Q) (O’Nions et al 2014a).

• Extreme Demand Avoidance Questionnaire for Adults (Egan et al 2018).

• Original 15 Diagnostic Interview for Social and Communication Disorders (DISCO) 15 (Gillberg et al 2015).

• Revised Diagnostic Interview for Social and Communication Disorders (DISCO) PDA 11 items.

• Semi-structured 22 Item semi-structured interview (O’Nions 2013; O’Nions et al 2018). I have scoring for rules for this tool from Elizabeth Newson Centre. If possible, the tool should be attached for authors own reference.

The ASSQ-REV was also used to study children. Haire et al (2024) scoping review for investigating PDA in children and adolescents seems relevant to the manuscript. The manuscript should be updated to reflect this.

Thank you for this feedback. Because our goal is to develop a parent-report questionnaire rather than an interview-based assessment, we have focused our literature review on existing parent-report survey measures (rather than diagnostic interviews). That said, we also note how the DISCO has been used to evaluate a PDA-related behavioral cluster.

Other alternative hypotheses for PDA include, PDA is a form of Attachment Disorder (Christie 2007 editorial; Flackhill et al 2017), & Personality Disorder (Christie 2007 editorial). There are PDA cases diagnosed independent of autism. Liz O’Nions PhD thesis has a few. Chapter 8 has 3 diagnosed under Conduct Problems, and the Qualitative study contains one diagnosed with Attachment disorder and scored 1 on the ADOS. The manuscript’s alternative hypotheses for PDA, should be updated to include as a form of Attachment Disorder, and Personality Disorder.

Thank you. We have added these points as requested.

Final specific comments.

L372-375. Why are Autistics expressing more demand-avoidance features than others?

We do not yet know. We hypothesize that PDA may represent a profile within autism. Alternatively, it may be that demand avoidance is a broader symptom that is exacerbated in neurodivergent individuals due to processing differences and related challenges. Future work is needed to clarify these possibilities. We have added these points to the discussion.

L262+L3673. If autism a co-occurring construct to PDA, then logically PDA cannot be a “Profile of autism” (A+B≠A). Perhaps, the manuscript can be amended to reflect this fallacy in the logic of PDA?

We have revised the text as requested to note that this presentation may reflect a profile within autism, or alternatively, a distinct profile.

L391-393. Originally, PDA was attributed as a standalone category. From around 2015, PDA was mainly attributed as dual ASD+PDA. Perhaps, it a more accurate phrasing is autism with, and without PDA formulations. The “PDA Profile of ASD” lobby tends to describe attributions as “formulations”, not “diagnoses”. This may be different outside of the UK. Please can the authors reflect upon this, and a suitable note to the manuscript on this point.

As mentioned above, we have reframed the text as requested to introduce the possibility that this may be a profile of autism, or that it may be unique and have included discussion around how future work will be needed to better understand this.

Also likely that the caregivers in the sample had predefined notions of PDA could be, and may have biased the study’s results (Kildahl et al 2021). Similar risks of bias, as other PDA studies seem to be present. Please can this be reflected upon, and discussed in the manuscript.

As noted above, we have reframed the text as requested to introduce the possibility that this may reflect a profile within autism, or alternatively, a distinct profile, and we have added discussion highlighting the need for future work to clarify this.

L396-397. Lauren Good’s (2016) Doctoral thesis may be of assistance here.

We now reference her doctoral thesis in the discussion.

PDAers might respond to CAST, comparable to Autistics, but for different reasons than Autistics do (O’Nions et al 2014b, p541). This needs to be reflected upon and discussed in the manuscript.

We agree and have adjusted the text to clarify this point.

Critique of PDA by both Milton and Woods are alluded to in the manuscript through citation of Moore (2020). They should be explicitly recognised as PDAers in the manuscript and cited, like their PDAer peers.

Thank you. We have added these citations.

Attachment Submitted filename: response to reviewer 20260423.docx

# Peer review: Decision Letter 1

**DOI:** 10.1371/journal.pone.0345827.r003

**Authors:** Jenna Scaramanga

27 Jul 2026

Dear Dr. Carlozzi,

Thank you for submitting your manuscript to PLOS ONE. After careful consideration, we feel that it has merit but does not fully meet PLOS ONE’s publication criteria as it currently stands. Therefore, we invite you to submit a revised version of the manuscript that addresses the points raised during the review process.

Please submit your revised manuscript by Sep 07 2026 11:59PM. If you will need more time than this to complete your revisions, please reply to this message or contact the journal office at plosone@plos.org. When you're ready to submit your revision, log on to https://www.editorialmanager.com/pone/ and select the 'Submissions Needing Revision' folder to locate your manuscript file.

- A letter that responds to each point raised by the academic editor and reviewer(s). You should upload this letter as a separate file labeled 'Response to Reviewers'.
- A marked-up copy of your manuscript that highlights changes made to the original version. You should upload this as a separate file labeled 'Revised Manuscript with Track Changes'.
- An unmarked version of your revised paper without tracked changes. You should upload this as a separate file labeled 'Manuscript'.

If you would like to make changes to your financial disclosure, please include your updated statement in your cover letter. Guidelines for resubmitting your figure files are available below the reviewer comments at the end of this letter.

If applicable, we recommend that you deposit your laboratory protocols in protocols.io to enhance the reproducibility of your results. Protocols.io assigns your protocol its own identifier (DOI) so that it can be cited independently in the future. For instructions see: https://journals.plos.org/plosone/s/submission-guidelines#loc-laboratory-protocols. Additionally, PLOS ONE offers an option for publishing peer-reviewed Lab Protocol articles, which describe protocols hosted on protocols.io. Read more information on sharing protocols at https://plos.org/protocols?utm_medium=editorial-email&utm_source=authorletters&utm_campaign=protocols.

As the corresponding author, your ORCID iD is verified in the submission system and will appear in the published article. PLOS supports the use of ORCID, and we encourage all coauthors to register for an ORCID iD and use it as well. Please encourage your coauthors to verify their ORCID iD within the submission system before final acceptance, as unverified ORCID iDs will not appear in the published article. *Only*  the individual author can complete the verification step; PLOS staff *cannot*  verify ORCID iDs on behalf of authors.

We look forward to receiving your revised manuscript.

Kind regards,

Jenna Scaramanga

Staff Editor

PLOS One

Journal Requirements:

If the reviewer comments include a recommendation to cite specific previously published works, please review and evaluate these publications to determine whether they are relevant and should be cited. There is no requirement to cite these works unless the editor has indicated otherwise.

[Note: HTML markup is below. Please do not edit.]

Reviewers' comments:

Reviewer's Responses to Questions

**Comments to the Author**

Reviewer #1: All comments have been addressed

Reviewer #2: All comments have been addressed

**********

2. Is the manuscript technically sound, and do the data support the conclusions?

Reviewer #1: Partly

Reviewer #2: Yes

**********

3. Has the statistical analysis been performed appropriately and rigorously?

Reviewer #1: Yes

Reviewer #2: Yes

**********

4. Have the authors made all data underlying the findings in their manuscript fully available?

The PLOS Data policy

Reviewer #1: Yes

Reviewer #2: Yes

**********

5. Is the manuscript presented in an intelligible fashion and written in standard English?

Reviewer #1: Yes

Reviewer #2: Yes

**********

Reviewer #1: Please see attachment.

Reviewer #2: The revised version of the study is methodologically sound, and I have no major reservations regarding the overall methodological rigour. I would like to raise two observations that I believe warrant further explanation or discussion:

1. Sample composition skew towards female parents. The majority of participants (681) identified as female parents, while the remaining participants represented other categories. Could the authors comment on whether this imbalance may have introduced any confounding influence on the results, and whether this limitation is acknowledged in the manuscript?

2. Factor loading magnitudes in the EFA. Several of the factor loadings in the EFA solution are close to the conventional minimum threshold (≥ .33), rather than reflecting a higher level of loading strength. I would encourage the authors to briefly discuss this in the text — for example, by clarifying the cutoff criterion used, noting any items with weaker or cross-loadings, and commenting on the implications this may have for the robustness of the factor structure.

Addressing these two points would further strengthen the manuscript.

**********

what does this mean?). If published, this will include your full peer review and any attached files.

If you choose “no”, your identity will remain anonymous but your review may still be made public.

**Do you want your identity to be public for this peer review?**  For information about this choice, including consent withdrawal, please see our Privacy Policy

Reviewer #1: **Yes:**  Richard Woods

Reviewer #2: **Yes:**  Sagayaraj Kanagaraj

**********

[NOTE: If reviewer comments were submitted as an attachment file, they will be attached to this email and accessible via the submission site. Please log into your account, locate the manuscript record, and check for the action link "View Attachments". If this link does not appear, there are no attachment files.]

To ensure your figures meet our technical requirements, please review our figure guidelines: https://journals.plos.org/plosone/s/figures

You may also use PLOS’s free figure tool, NAAS, to help you prepare publication quality figures: https://journals.plos.org/plosone/s/figures#loc-tools-for-figure-preparation.

NAAS will assess whether your figures meet our technical requirements by comparing each figure against our figure specifications.

Attachment Submitted filename: June_2026_Plos_One_Peer_Review.docx

# Author comment: Author response to Decision Letter 2

**DOI:** 10.1371/journal.pone.0345827.r004

19 Aug 2026

Response to Reviewers

Reviewer #1:

1) I have searched for it, and I apologise if I missed it. The hypothesis of the significantly higher threshold from avoidance to “ordinary” demands, instead of “everyday” demands (Company and Rostella 2026), needs to be mentioned when discussing how heightened drive for autonomy has become proposed to explain PDA.

Thank you for referencing the Company and Rotella article and noting the shift from “ordinary” to “everyday” demands. We have added text acknowledging Newson’s original use of the phrase “ordinary demands” and how several organizations have extended this framing to encompass “everyday” or “common” demands, including basic self-care activities (i.e., hygiene, eating, sleeping, toileting).

We further emphasize that avoidance of these demands is a cornerstone of our proposed definition of PDA that differentiates the new DAAQ measure from existing measures that do not assess functional impact on basic needs.

We also now include discussion of how PDA has come to be associated with heightened drive for autonomy, and clarify that autonomy-centered PDA accounts propose – but have not established – that ordinary demands may be experienced as disproportionately threatening to autonomy or control.

2) The discussion on a heightened drive for autonomy needs to mention the hypothesis heightened drive for autonomy is due to a rhetorical repositioning of PDA as an ASD profile (Company and Rostella 2026).

We appreciate this point and have added Company and Rostella (2026) to acknowledge the hypothesis that autonomy-centered accounts may represent a rhetorical repositioning of PDA as an autism profile. We also clarified our own position. The autonomy-centered formulation used in developing the DAAQ was not derived from an established empirical literature, which remains limited, but was developed as a hypothesis-generating framework informed by lived-experience accounts, practitioner observation, Self-Determination Theory, and preliminary stakeholder input. We agree that reframing a descriptive behavioral profile as a “drive for autonomy” moves toward an explanatory formulation and therefore requires direct empirical testing. The present study evaluates the DAAQ’s factor structure and preliminary psychometric properties; it does not establish perceived autonomy threat as the cause of demand avoidance or determine whether PDA is an autism profile.

3) Most predictions on PDA populations are based on Newson’s descriptions, such as (Gillberg 2014). This needs to be explicitly stated and considered when justifying the tool’s underpinning model. I apologise, if I missed this, but the similarities, and differences of the tool’s underpinning model, to Newson’s descriptions need to be mentioned for ease of reference.

Thank you for this helpful comment. We agree that Newson’s original clinical description has served as the foundation for much of the subsequent empirical and theoretical work on PDA. We have revised the Introduction to make this lineage more explicit.

4) Company and Rostella (2026) critique of the EDA-Q should be included in the literature review.

This review publication is now cited in several places in the manuscript.

5) The shift using a formulation, instead of a descriptive diagnosis for PDA needs to be mentioned. See the Kieran case study as example. Phil Christie, 5≥ years ago (e.g., see their talks in 2016-2019), in their various conference talks, (accurately) mentioned how psychiatric categories are based on reliability over validity, and proceeded onto reasons for using a diagnosis in research and practice settings. The hypothesis the change to a PDA formulation, from a diagnosis is due to a rhetorical repositioning of PDA as an ASD profile (Company and Rostella 2026). This matters as using a heightened drive for autonomy and equality, is used as an explanation for a person’s behaviours, instead of describing them. Changing PDA from a descriptive category to an explanatory one is MASSIVE change in PDA’s ontology, when there is insufficient evidence to warrant it. This topic needs to be discussed, even if manuscript disagrees with my views on the topic.

The Introduction now explicitly describes the autonomy-centered account as a provisional, hypothesis-generating formulation, not an established explanation, and acknowledges the limited empirical literature regarding PDA mechanisms. The limitations further recognize that moving from a descriptive behavioral profile to an autonomy-centered explanation represents a rhetorical and conceptual reframing that requires empirical evaluation, with Company and Rostella (2026) cited in support of this point. We also clarify that the DAAQ assesses behavioral expression and functional impact rather than the reasons or mechanisms underlying demand avoidance.

6) I appreciate changing the tool’s name. I am struggling to see how the current name is accurate, or helpful. This logic is suitable A+B≠A. I say this because it is called demand avoidance and autonomy questionnaire. Yet, the heightened drive for autonomy and equality is meant to be model underpinning the tool, how it can it be both (i.e., demand-avoidance itself is broader than a heightened drive for autonomy, or a heightened drive for equality. Furthermore, a heightened drive for autonomy, is only meant to be part of the causes of PDA’s demand-avoidance, according to the tool’s underpinning model)? I am not entirely surely that the model, is a model for heightened drive for autonomy and equality. The manuscript describes a drive for autonomy more than a heightened drive for equality. How well is a heightened drive for equality captured in the tool’s items? If the tool significantly assesses for a heightened drive for equality, then the tool’s name should be changed to reflect this. If the tool does not, then conceptual model underlying the tool should be updated to reflect it is not sufficiently capturing a heightened drive for equality, or be this should be noted in the limitations.

Thank you for this comment. We agree that the prior wording may have blurred the distinction between demand avoidance as the behavioral phenomenon being measured, autonomy as the primary theoretical lens guiding item development, and equality/equalizing behavior as a related but not independently scored construct. We have retained the name Demand Avoidance and Autonomy Questionnaire because it reflects the measure’s focus on observable demand-avoidance-related behaviors and an autonomy-centered conceptual framework. However, we revised the manuscript to clarify that the DAAQ does not assess all possible causes of demand avoidance and does not provide a distinct measure of heightened drive for equality. We also softened language implying that autonomy and equality were measured equivalently and added a limitation noting that equalizing behavior is represented only within the broader Behavioral Expression domain. We also now clarify the relationship among demand avoidance, autonomy, and equality.

7) Equality, as a concept is highly subjective. Values behind equality can rapidly change, and are transient; one only has to look at debates around right to vote, and/ or slavery, for different values for equality. If a heightened drive for equality conceptually underpins this tool, then the subjective nature of equality needs to be addressed.

We agree that “equality” is a subjective and culturally embedded concept, and that the manuscript needed to avoid implying a fixed or universal definition of equality. We have revised the text to clarify that equality is not treated as a standalone construct underpinning the DAAQ, nor does the measure attempt to assess values-based beliefs about equality. Specifically, we indicate that this measure assesses perceived control, fairness, or relational balance.

8) I presented an open ended question, for potential causes of Autistics scoring higher for the tool’s 3 subscales, to see if manuscript may consider environmental and cultural factors might contribute to these results. How poorly treated Autistics are by Global North cultures, is a large factor behind the atrocious mental health issues found in the Autistic population, and suicide related problems (Autistic scholars discuss these points, arguably Monique Botha’s work is amongst most important). Most people, who are as distressed and traumatised as many Autistics are, are likely to score highly on at least 1 of the tool domains. Additionally, it seems reasonable to question, if the result is at least partly caused by how PDA is often modelled as a “Profile of ASD”, identified in Autistics, and researched in Autistics, that this can potentially bias the tool towards Autistics. It is worth noting the hypothesis heightened drive for autonomy is due to a rhetorical repositioning of PDA as an ASD profile (Company and Rostella 2026).

Thank you for clarifying this point. We agree that higher DAAQ scores among children with co-occurring autism may reflect multiple influences and should not be interpreted as evidence that PDA is necessarily an autism profile. We revised the Discussion to acknowledge that environmental and cultural factors, including poorly adapted contexts, unmet support needs, stigma, and trauma/minority-stress-related processes, may contribute to elevated scores among autistic children. We also note that because PDA has often been conceptualized and studied in relation to autism, this may have influenced caregiver recognition and item development.

9) Unless I am missing the citations in the paragraph discussing the study’s hyperactivity and conduct issues results, they need to be situation in broader literature on the topic.

Thank you for noting this. We revised the Discussion to situate the observed associations between the DAAQ Behavioral Expression factor and SDQ Hyperactivity and Conduct Problems within the broader literature.

10) I apologise, if I missed it. May the words “prejudice”, and “stigma” be used in the manuscript? Even it they are not used to discuss potential risks of attributing PDA to an individual, some have argued a heightened drive for autonomy, is less stigmatising than using “Pathological Demand Avoidance” term.

Thank you for this suggestion. We agree that the language used to describe PDA/demand avoidance has implications for stigma and potential prejudice and have added this to the Introduction.

11) One of the most replicated results in PDA studies, is how behaviours assessed for by PDA tools decrease as a CYP grows into adulthood. How does this study’s results compare to those results?

Thank you. Given that the current study was cross-sectional and limited to parent reports for children/adolescents ages 4–17 (i.e., it was not designed to evaluate developmental trajectories or compare child/adolescent scores with adult presentations), we have added this as an area of future research.

12) Thank you mentioning the Attachment disorder hypothesis.

Thank you for this feedback.

13) Sample size is an indication of the passion for the PDA out there. Also reflects many other PDA studies.

Thank you for this observation. We agree.

14) L187-189: It is obvious, that researchers are going to continue to hold PDA to typical standards, which are there to protect people (Company and Rostella 2026). An alternative outlook to families feeling unseen etc, is to point to those exposing the vulnerable persons to PDA’s controversies, by prematurely reifying PDA. This is a factor why Green et al (2018), suggest utilising features from co-occurring difficulties to model PDA.

We revised the introduction to address these concerns.

15) L191. “broader autism spectrum presentations,”. This is a contested opinion. Yes, many view PDA to be a “profile of ASD”, but this could be due to a hypothesised rhetorical repositioning of PDA as an ASD profile. At least before 2015, PDA’s literature tended to explicitly note which features make it problematic features contradict autism understandings. That phrasing needs to be changed.

We revised this wording to be more neutral and to avoid implying that PDA is established as a subtype or profile of autism.

16) L214-218. This is conjecture. As pointed out in the first review. There are good grounds to question in PDA’s demand-avoidance is intrinsically to “everyday” demands, such as those associated with toileting etc. Again good reasons to question, if avoidance to demands, like hygiene matters should be associated with PDA. It is possible to gain statistics of use of EDA-Q. The manuscript needs to provide the numbers/ percentages of studies utilising EDA-Q, in Kildahl et al (2021), Company and Rostella (2026), and Haire et al (2024) respective reviews. It might also be worth noting that not all datasets including the EDA-Q have been analysed; from my understanding Help4Psychology have not had their EDA-Q data analysed for potential correlations with any tools they used as part of their assessments, such as the Short Sensory Profile. It might be worth contacting Help4Psychology, if this is still the case, and possibly comment on the topic in the manuscript.

We have revised the text to clarify that the DAAQ assesses functional impact on basic-needs because these domains are frequently reported by families and were part of our stakeholder-informed item-development process, not because we assume that avoidance of eating, sleeping, toileting, or hygiene is uniquely or necessarily specific to PDA. We also removed/softened language suggesting “limited uptake” of the EDA-Q/EDA-8 to avoid making a claim that would require a systematic accounting of measure use across reviews. We did not add analyses of external datasets, as this was beyond the scope of the present measure-development study.

17) L265. Notes purpose of the study was for the tool to capture PDA specific features. Considering the literature generally notes PDA lacks any specific features; this objective needs to be explicitly addressed in the literature review. It is worth stating PDA does not need to have any specific features for it to be included in the psychiatric manuals, as most categories and characteristics operate as continua, and overlapping each other. Most categories in the DSM lack specific causal mechanisms.

Thank you for this point. We agree that the term “PDA-specific” could imply that the literature has established features unique to PDA, which was not our intent. We revised the manuscript to use “PDA-related” throughout.

18) L292. “An initial pool of 67 questions was designed to evaluate the key”, implies those 67 items describe PDA, which as the manuscript notes, may not be the case. Please could it be changed to something like “An initial pool of 67 questions was designed to evaluate the hypothesised key”.

We have made this change as requested.

Reviewer #2:

The revised version of the study is methodologically sound, and I have no major reservations regarding the overall methodological rigour. I would like to raise two observations that I believe warrant further explanation or discussion:

1. Sample composition skew towards female parents. The majority of participants (681) identified as female parents, while the remaining participants represented other categories. Could the authors comment on whether this imbalance may have introduced any confounding influence on the results, and whether this limitation is acknowledged in the manuscript?

Thank you for raising this point. We have revised the Discussion to explicitly acknowledge this limitation and to note that DAAQ scores should be interpreted relative to the predominantly female caregiver calibration sample. We also added language recommending future validation studies with more diverse caregiver samples and multi-informant designs.

2. Factor loading magnitudes in the EFA. Several of the factor loadings in the EFA solution are close to the conventional minimum threshold (≥ .33), rather than reflecting a higher level of loading strength. I would encourage the authors to briefly discuss this in the text — for example, by clarifying the cutoff criterion used, noting any items with weaker or cross-loadings, and commenting on the implications this may have for the robustness of the factor structure.

We now clarify the cutoff criterion used (≥ .30); this criterion is consistent with the early-stage measure-development context and our goal of preserving conceptually important content for subsequent evaluation. We also now explicitly note that several items had relatively modest loadings and that one item showed cross-loading. Importantly, EFA was not used as the sole basis for final item retention. We also added language to the Discussion acknowledging that modest EFA loadings and cross-loadings suggest that some PDA-related behaviors may be heterogeneous and that future replication in independent, more diverse samples is needed.

Attachment Submitted filename: response to reviewer 20260819(2).docx

# Peer review: Decision Letter 2

**DOI:** 10.1371/journal.pone.0345827.r005

**Authors:** Vanessa Carels

14 Sep 2026

Dear Dr. Carlozzi,

Thank you for submitting your manuscript to PLOS One. After careful consideration, we feel that it has merit but does not fully meet PLOS One’s publication criteria as it currently stands. Therefore, we invite you to submit a revised version of the manuscript that addresses the points raised during the review process.

Please submit your revised manuscript by Oct 29 2026 11:59PM. If you will need more time than this to complete your revisions, please reply to this message or contact the journal office at plosone@plos.org. When you're ready to submit your revision, log on to https://www.editorialmanager.com/pone/ and select the 'Submissions Needing Revision' folder to locate your manuscript file.

- A letter that responds to each point raised by the academic editor and reviewer(s). You should upload this letter as a separate file labeled 'Response to Reviewers'.
- A marked-up copy of your manuscript that highlights changes made to the original version. You should upload this as a separate file labeled 'Revised Manuscript with Track Changes'.
- An unmarked version of your revised paper without tracked changes. You should upload this as a separate file labeled 'Manuscript'.

If you would like to make changes to your financial disclosure, please include your updated statement in your cover letter. Guidelines for resubmitting your figure files are available below the reviewer comments at the end of this letter.

If applicable, we recommend that you deposit your laboratory protocols in protocols.io to enhance the reproducibility of your results. Protocols.io assigns your protocol its own identifier (DOI) so that it can be cited independently in the future. For instructions see: https://journals.plos.org/plosone/s/submission-guidelines#loc-laboratory-protocols. Additionally, PLOS One offers an option for publishing peer-reviewed Lab Protocol articles, which describe protocols hosted on protocols.io. Read more information on sharing protocols at https://plos.org/protocols?utm_medium=editorial-email&utm_source=authorletters&utm_campaign=protocols.

As the corresponding author, your ORCID iD is verified in the submission system and will appear in the published article. PLOS supports the use of ORCID, and we encourage all coauthors to register for an ORCID iD and use it as well. Please encourage your coauthors to verify their ORCID iD within the submission system before final acceptance, as unverified ORCID iDs will not appear in the published article. *Only*  the individual author can complete the verification step; PLOS staff *cannot*  verify ORCID iDs on behalf of authors.

We look forward to receiving your revised manuscript.

Kind regards,

Vanessa Carels

Staff Editor

PLOS One

**Journal Requirements:**

If the reviewer comments include a recommendation to cite specific previously published works, please review and evaluate these publications to determine whether they are relevant and should be cited. There is no requirement to cite these works unless the editor has indicated otherwise.

[Note: HTML markup is below. Please do not edit.]

Reviewers' comments:

Reviewer's Responses to Questions

**Comments to the Author**

Reviewer #1: (No Response)

Reviewer #2: All comments have been addressed

**********

2. Is the manuscript technically sound, and do the data support the conclusions?

Reviewer #1: Yes

Reviewer #2: Yes

**********

3. Has the statistical analysis been performed appropriately and rigorously?

Reviewer #1: Yes

Reviewer #2: Yes

**********

4. Have the authors made all data underlying the findings in their manuscript fully available?

The PLOS Data policy

Reviewer #1: Yes

Reviewer #2: Yes

**********

5. Is the manuscript presented in an intelligible fashion and written in standard English?

Reviewer #1: Yes

Reviewer #2: Yes

**********

Reviewer #1: SEPTEMBER 2026 PLOS ONE PEER REVIEW.

The manuscript is significantly strengthened over previous iterations. I appreciate the time and effort put in to do this. I am not entirely sure the manuscript feedback was necessarily acted upon.

There are a couple of points of I think the manuscript should address.

I am not entirely sure that Newson’s portrayal of “ordinary” demands reflects that in the paper. Newson’s account also seems focus on the impact of the demand-avoidance and other behaviours on the caregivers, and others around the PDAer. I think it is worth quoting this part from the first peer review.:

“I am unsure if the construct of PDA in this manuscript is the same put forward by Newson, and later knowledge derived from Newson’s work. For instance, The study states its deliberately focused on toileting and hygiene difficulties of PDAers. I accept that this a problem for some PDAers. Toileting and hygiene issues discussed in the manuscript are sufficient that they should have been picked up key previous PDA scholarship before this. These issues should be problematic enough to be brought up by caregivers, and others around the PDAers during the clinical assessment process. The toileting and other issues should be collected as part of a comprehensive assessment for autism, or other Neurodevelopmental category, if only for excluding other explanations for an assessee’s behaviour.

Kildahl et al (2021) noted that Newson’s PDA conceptualisation is a mess, with many assumptions. It was this as Newson and colleagues including any significant features they observed in their PDAers. Searching Newson et al (2003) and supplementary notes using: “clean”, “hygiene”, & “toilet”, only yielded 1 instance, relation to obsessions on “over cleanliness/slovenliness” (Newson et al 2003, 598). The same 3 search terms were used on O’Nions et (2016a) DISCO paper, and its supplementary files and produced 0 results. This result was replicated in O’Nions et al (2014a) EDA-Q development paper, and O’Nions et al (2018) qualitative examination of ASD+PDA. A 22 item semi-structured interview used to assess for PDA from before 2015, does includes mentions of urinating in public in terms embarrassing and socially shocking behaviour. Considering the tools assesses for serious features, including self-injury. If toileting issues were present, they should be included in the tool. Should toileting issues be associated with PDA? Is it reflective of PDA? Are associating toileting and hygiene issues with PDA, likely to contribute to stigma and/ or prejudice experiencing by PDAers?”

Even, if this how “ordinary” demands are accurately portrayed in the manuscript, others would view avoidance of “ordinary” demands to be avoidance typical Global North demands, such as in the Keiran case study from Soppitt (2021), or as argued by Critical Autism Studies scholars. In particular Allison Moore argues a person is more likely to be attributed with PDA, when the contravene cultural norms associated with autism, childhood, and female gender. This is reflected in how some have suggested PDA might be a female presentation of autism (Eaton 2018; Gould and Ashton-Smith 2011). Furthermore, there is wide variation in how PDA is identified in research and clinical settings, with some “PDA Profile of ASD” advocates claiming PDA is over identified, e.g., PDA Society. There is no consensus that “ordinary” demands as described in PDA, is what is meant as representative of PDA. Newson meant “ordinary” demands as described in the manuscript, the tool should not need to be developed, as those features would already be assessed for previous PDA tools. The manuscript needs to address how broader and diverse interprets what “ordinary” demands means.

The underlying model for the tool needs to address how demand-avoidance is a transactional process, and that it should it be possible for a person to develop PDA after early infancy, such as being distressed and/ or traumatised into expressed demand-avoidance behaviours for sufficient period of time, that these behaviours generalise (Kildahl et al 2021). Furthermore, the model needs to account for one of most replicated results in PDA studies in CYP, that PDA behaviours tend to significantly decrease a CYP as the CYP approaches adulthood (this rate is tends to substantially higher than seen in autism, and in some studies exceeding the rates observed in ADHD). Stuart et al (2020) hypotheses this apparent feature of PDA is due to PDAers developing better coping mechanisms. This replicated feature raises reasonable doubts that PDA is intrinsically pervasive, or developmental in nature. This feature needs to be accounted for in the manuscript’s model underlying the tool.

L328. “Interested parents of children with a suspected PDA profile”, the change in wording to add “Profile” might be pertinent. It is a choice to frame PDA as a “Profile”, it suggests a level of preconceived notions over what PDA could be and how it is modelled. This is addressed as a potential limitation.

Reviewer #2: The authors have satisfactorily addressed the comments and concerns raised by the reviewers. The revisions have improved the clarity, methodological presentation, interpretation of the findings, and overall quality of the manuscript. The manuscript is suitable for publication in its revised form.

**********

what does this mean?). If published, this will include your full peer review and any attached files.

If you choose “no”, your identity will remain anonymous but your review may still be made public.

**Do you want your identity to be public for this peer review?**  For information about this choice, including consent withdrawal, please see our Privacy Policy

Reviewer #1: **Yes:**  Richard Woods

Reviewer #2: **Yes:**  Sagayaraj Kanagaraj

**********

[NOTE: If reviewer comments were submitted as an attachment file, they will be attached to this email and accessible via the submission site. Please log into your account, locate the manuscript record, and check for the action link "View Attachments". If this link does not appear, there are no attachment files.]

To ensure your figures meet our technical requirements, please review our figure guidelines: https://journals.plos.org/plosone/s/figures

You may also use PLOS’s free figure tool, NAAS, to help you prepare publication quality figures: https://journals.plos.org/plosone/s/figures#loc-tools-for-figure-preparation.

NAAS will assess whether your figures meet our technical requirements by comparing each figure against our figure specifications.

# Author comment: Author response to Decision Letter 3

**DOI:** 10.1371/journal.pone.0345827.r006

30 Sep 2026

Response to Reviewers

Reviewer #1:

1) The manuscript is significantly strengthened over previous iterations. I appreciate the time and effort put in to do this.

Thank you for this positive feedback.

2) I am not entirely sure the manuscript feedback was necessarily acted upon. There are a couple of points of I think the manuscript should address. I am not entirely sure that Newson’s portrayal of “ordinary” demands reflects that in the paper. Newson’s account also seems focus on the impact of the demand-avoidance and other behaviours on the caregivers, and others around the PDAer.

Thank you for raising this point. We revised the Introduction to more precisely represent Newson’s original account. Specifically, we now clarify that Newson used “ordinary demands” to encompass what she described as “normal expectations,” ordinary group demands in nursery or school, educational demands, organizational rules, and adult-directed tasks. We also acknowledge her emphasis on the impact of these behaviors on parents and teachers. Finally, we distinguish Newson’s account from our observation that what is considered an ordinary or developmentally typical demand may vary across families, educational settings, social contexts, and cultures. The manuscript now states:

“Newson characterized PDA as ‘a distinct behavioral profile involving obsessional avoidance of the ordinary demands of life, coupled with a degree of sociability that allowed social manipulation as a primary avoidance strategy.’[3] Her account used ‘ordinary demands’ to encompass ‘normal expectations,’ ordinary group demands in nursery or school, educational demands, organizational rules, and adult-directed tasks. Newson also emphasized the impact of these behaviors on parents and teachers, describing the children as particularly difficult for them to manage and teach. However, Newson did not provide a discrete or culturally neutral operational definition of what makes a demand ‘ordinary.’ What is regarded as an ordinary or developmentally typical expectation may vary across families, educational settings, social contexts, and cultures.[10]”

3) I think it is worth quoting this part from the first peer review.:

“I am unsure if the construct of PDA in this manuscript is the same put forward by Newson, and later knowledge derived from Newson’s work. For instance, The study states its deliberately focused on toileting and hygiene difficulties of PDAers. I accept that this a problem for some PDAers. Toileting and hygiene issues discussed in the manuscript are sufficient that they should have been picked up key previous PDA scholarship before this. These issues should be problematic enough to be brought up by caregivers, and others around the PDAers during the clinical assessment process. The toileting and other issues should be collected as part of a comprehensive assessment for autism, or other Neurodevelopmental category, if only for excluding other explanations for an assessee’s behaviour.”

We clarified that hygiene and toileting are not established as defining or PDA-specific features. Although these domains were not prominent in Newson’s original account or systematically assessed in prior PDA measures, stakeholder input identified them as potentially important functional difficulties among some children with marked demand avoidance. We also note that they may have multiple developmental, medical, sensory, psychological, or contextual explanations and require comprehensive assessment. The manuscript now states:

“Hygiene and toileting difficulties were not prominent features of Newson’s original account and are not systematically assessed in prior PDA instruments. Nevertheless, stakeholder input identified these as potentially important functional difficulties among some children with marked demand avoidance. They may have multiple developmental, medical, sensory, psychological, or contextual explanations and should not be assumed to be core or specific features of PDA.”

When introducing the DAAQ, we further characterize these domains as “potentially associated functional difficulties involving basic needs … that emerged through stakeholder input.”

4) Kildahl et al (2021) noted that Newson’s PDA conceptualisation is a mess, with many assumptions. It was this as Newson and colleagues including any significant features they observed in their PDAers. Searching Newson et al (2003) and supplementary notes using: “clean”, “hygiene”, & “toilet”, only yielded 1 instance, relation to obsessions on “over cleanliness/slovenliness” (Newson et al 2003, 598). The same 3 search terms were used on O’Nions et (2016a) DISCO paper, and its supplementary files and produced 0 results. This result was replicated in O’Nions et al (2014a) EDA-Q development paper, and O’Nions et al (2018) qualitative examination of ASD+PDA. A 22 item semi-structured interview used to assess for PDA from before 2015, does includes mentions of urinating in public in terms embarrassing and socially shocking behaviour. Considering the tools assesses for serious features, including self-injury. If toileting issues were present, they should be included in the tool. Should toileting issues be associated with PDA? Is it reflective of PDA? Are associating toileting and hygiene issues with PDA, likely to contribute to stigma and/ or prejudice experiencing by PDAers?”

As noted above, we now clarify that existing literature has not established hygiene and toileting as core or PDA-specific features. Their omission from earlier measures does not preclude assessing them as potentially associated functional difficulties identified through stakeholder input. We also emphasize that these difficulties may have multiple explanations, require contextual interpretation, and should not be used diagnostically. We agree that attributing intimate functional difficulties to PDA without appropriate context could contribute to stigma; accordingly, we emphasize contextual and non-diagnostic interpretation.

5) Even, if this how “ordinary” demands are accurately portrayed in the manuscript, others would view avoidance of “ordinary” demands to be avoidance typical Global North demands, such as in the Keiran case study from Soppitt (2021), or as argued by Critical Autism Studies scholars. In particular Allison Moore argues a person is more likely to be attributed with PDA, when the contravene cultural norms associated with autism, childhood, and female gender. This is reflected in how some have suggested PDA might be a female presentation of autism (Eaton 2018; Gould and Ashton-Smith 2011). Furthermore, there is wide variation in how PDA is identified in research and clinical settings, with some “PDA Profile of ASD” advocates claiming PDA is over identified, e.g., PDA Society. There is no consensus that “ordinary” demands as described in PDA, is what is meant as representative of PDA. Newson meant “ordinary” demands as described in the manuscript, the tool should not need to be developed, as those features would already be assessed for previous PDA tools. The manuscript needs to address how broader and diverse interprets what “ordinary” demands means.

We agree that “ordinary demands” is neither standardized nor culturally neutral, and we revised the manuscript to acknowledge variation across families, developmental and educational contexts, and cultures. We also clarified in the study-purpose paragraph that the DAAQ does not assume that such demands are objectively ordinary or benign. However, we respectfully disagree that overlap with Newson’s terminology removes the need for a new measure. The DAAQ expands stakeholder-informed content coverage and applies contemporary psychometric methods not used in developing earlier measures.

6) The underlying model for the tool needs to address how demand-avoidance is a transactional process, and that it should it be possible for a person to develop PDA after early infancy, such as being distressed and/ or traumatised into expressed demand-avoidance behaviours for sufficient period of time, that these behaviours generalise (Kildahl et al 2021). Furthermore, the model needs to account for one of most replicated results in PDA studies in CYP, that PDA behaviours tend to significantly decrease a CYP as the CYP approaches adulthood (this rate is tends to substantially higher than seen in autism, and in some studies exceeding the rates observed in ADHD). Stuart et al (2020) hypotheses this apparent feature of PDA is due to PDAers developing better coping mechanisms. This replicated feature raises reasonable doubts that PDA is intrinsically pervasive, or developmental in nature. This feature needs to be accounted for in the manuscript’s model underlying the tool.

We agree that demand avoidance may be transactional and context-dependent, and we revised the conceptual model accordingly. The manuscript now states:

“This model is intended to generate testable hypotheses about one possible pathway to demand avoidance, not to imply that autonomy threat is its sole cause or that these processes are unique to PDA. Demand avoidance may also be transactional and context-dependent, emerging through interactions among the individual, the nature and delivery of demands, prior experiences, relationships, and environmental responses. Other neurodevelopmental and clinical presentations (e.g., autism, attention deficit hyperactivity disorder, obsessive-compulsive disorder, bipolar disorder, oppositional defiant disorder, and trauma-related conditions) may also involve a strong drive for autonomy or demand avoidance arising through sensory, cognitive, emotional, interpersonal, or contextual pathways. The present cross-sectional study cannot determine when demand avoidance develops, whether it generalizes over time, or whether it represents a stable developmental trait.”

Although sustained distress or trauma may be one pathway, current evidence does not support adopting a single causal model. We also expanded the Limitations:

“Longitudinal research is needed to examine whether and how demand-avoidance behaviors change as children approach adulthood. Although some prior work has reported decreases for some individuals, Newson’s follow-up sample showed heterogeneous trajectories, with avoidance decreasing in some participants, remaining stable in others, and increasing in a smaller number[3, 33]. The present cross-sectional study cannot establish that the measured behaviors are developmentally stable or intrinsically pervasive.”

7) L328. “Interested parents of children with a suspected PDA profile”, the change in wording to add “Profile” might be pertinent. It is a choice to frame PDA as a “Profile”, it suggests a level of preconceived notions over what PDA could be and how it is modelled. This is addressed as a potential limitation.

We agree that “suspected PDA profile” reflects a particular framing. This terminology was used in recruitment and by the participating community; it was not intended to establish PDA as a validated profile, autism subtype, or diagnosis. We clarified this in the Methods:

“The term ‘suspected PDA profile’ reflected the terminology used in recruitment and by the community from which the sample was drawn. Its use should not be interpreted as establishing PDA as a validated diagnostic profile or as resolving competing conceptualizations of demand avoidance.”

We also acknowledge in the Limitations that this terminology may have influenced participation and caregivers’ interpretation and reporting of their children’s behavior.

Reviewer #2:

1) The authors have satisfactorily addressed the comments and concerns raised by the reviewers. The revisions have improved the clarity, methodological presentation, interpretation of the findings, and overall quality of the manuscript. The manuscript is suitable for publication in its revised form.

Thank you for this positive feedback.

Attachment Submitted filename: response to reviewer 20260928.docx

# Peer review: Decision Letter 3

**DOI:** 10.1371/journal.pone.0345827.r007

**Authors:** Eugenio Llorens

21 Oct 2026

The Demand Avoidance and Autonomy Questionnaire (DAAQ): Development of a new measure of demand avoidance

PONE-D-25-64938R3

Dear Dr. Carlozzi,

We’re pleased to inform you that your manuscript has been judged scientifically suitable for publication and will be formally accepted for publication once it meets all outstanding technical requirements.

Within one week, you’ll receive an e-mail detailing the required amendments. When these have been addressed, you’ll receive a formal acceptance letter and your manuscript will be scheduled for publication.

An invoice will be generated when your article is formally accepted. Please note, if your institution has a publishing partnership with PLOS and your article meets the relevant criteria, all or part of your publication costs will be covered. Please make sure your user information is up-to-date by logging into Editorial Manager at Editorial Manager® and clicking the ‘Update My Information' link at the top of the page. For questions related to billing, please contact billing support.

If your institution or institutions have a press office, please notify them about your upcoming paper to help maximize its impact. If they’ll be preparing press materials, please inform our press team as soon as possible -- no later than 48 hours after receiving the formal acceptance. Your manuscript will remain under strict press embargo until 2 pm Eastern Time on the date of publication. For more information, please contact onepress@plos.org.

Kind regards,

Eugenio Llorens

Academic Editor

PLOS One

Additional Editor Comments (optional):

Reviewers' comments:

Reviewer's Responses to Questions

**Comments to the Author**

Reviewer #1: All comments have been addressed

Reviewer #2: All comments have been addressed

**********

2. Is the manuscript technically sound, and do the data support the conclusions?

Reviewer #1: Yes

Reviewer #2: Yes

**********

3. Has the statistical analysis been performed appropriately and rigorously?

Reviewer #1: Yes

Reviewer #2: Yes

**********

4. Have the authors made all data underlying the findings in their manuscript fully available?

The PLOS Data policy

Reviewer #1: Yes

Reviewer #2: Yes

**********

5. Is the manuscript presented in an intelligible fashion and written in standard English?

Reviewer #1: Yes

Reviewer #2: Yes

**********

Reviewer #1: OCTOBER 2026 PLOS ONE PEER REVIEW.

Thank you to the authors for acting on the feedback in the previous review. I am grateful to the authors for acting on the feedback. The manuscript seems suitable for publication.

Notes.

126-130. This is a hypothesis and needs being tested. If this argument is being made, the opposite outlook should be stated that, that framing PDA in terms of a heightened drive for autonomy is intrinsically an ableist outlook. Some Critical Autism Studies scholars have contended that framing PDA in this manner highlights how “Persistent Drive for Autonomy” is another functioning category, mainly aimed at speaking Autistics (the focus on issues with daily living suggest there is merit to that perspective). As previously mentioned, most/ all persons benefit from enabling a person’s autonomy, so framing PDA as a heightened “Drive for Autonomy” risks reifying, that non-PDAers have a lesser, or no “Drive for Autonomy”, through the spreading of harmful stereotypes, which could predictably lead to non-PDAers being denied strategies to work with non-PDAers autonomy. That hypothesis also needs to be tested.

L151-158. Indeed, the language used in Newson’s and most later PDA studies, at least in the 2010s may seem to indicate that avoidance to “ordinary” demands centres on non-compliance with others demands. This is supported by Newson’s focus on impact of demand-avoidance on others, and is represented in tools to assess for PDA features. A pertinent example is from Revised 11 PDA DISCO items in O’Nions et al (2016) supplementary files:

“Lack of co-operation (LACKCOP)

Does A strongly resist attempts to make her/him join in, learn new things, or to change behaviour; screams, has temper tantrums, scratches, bites, kicks or passively resists, or uses distracting techniques?”

Reviewer #2: The revisions have improved the manuscript’s clarity, methodological presentation, interpretation of findings, and overall quality.

**********

what does this mean?). If published, this will include your full peer review and any attached files.

If you choose “no”, your identity will remain anonymous but your review may still be made public.

**Do you want your identity to be public for this peer review?**  For information about this choice, including consent withdrawal, please see our Privacy Policy

Reviewer #1: **Yes:**  Richard Woods

Reviewer #2: No

**********

# Editor report: Acceptance letter

**DOI:** 10.1371/journal.pone.0345827.r008

**Authors:** Eugenio Llorens

PONE-D-25-64938R3

PLOS One

Dear Dr. Carlozzi,

I'm pleased to inform you that your manuscript has been deemed suitable for publication in PLOS One. Congratulations! Your manuscript is now being handed over to our production team.

At this stage, our production department will prepare your paper for publication. This includes ensuring the following:

* All references, tables, and figures are properly cited

* All relevant supporting information is included in the manuscript submission,

* There are no issues that prevent the paper from being properly typeset

You will receive further instructions from the production team, including instructions on how to review your proof when it is ready. Please keep in mind that we are working through a large volume of accepted articles, so please give us a few days to review your paper and let you know the next and final steps.

Lastly, if your institution or institutions have a press office, please let them know about your upcoming paper now to help maximize its impact. If they'll be preparing press materials, please inform our press team within the next 48 hours. Your manuscript will remain under strict press embargo until 2 pm Eastern Time on the date of publication. For more information, please contact onepress@plos.org.

You will receive an invoice from PLOS for your publication fee after your manuscript has reached the completed accept phase. If you receive an email requesting payment before acceptance or for any other service, this may be a phishing scheme. Learn how to identify phishing emails and protect your accounts at https://explore.plos.org/phishing.

If we can help with anything else, please email us at customercare@plos.org.

Thank you for submitting your work to PLOS One and supporting open access.

Kind regards,

PLOS One Editorial Office Staff

on behalf of

Dr. Eugenio Llorens

Academic Editor

PLOS One
